# Text mining occupations from the mental health electronic health record: a natural language processing approach using records from the Clinical Record Interactive Search (CRIS) platform in south London, UK

**DOI:** 10.1136/bmjopen-2020-042274

**Published:** 2021-03-25

**Authors:** Natasha Chilman, Xingyi Song, Angus Roberts, Esther Tolani, Robert Stewart, Zoe Chui, Karen Birnie, Lisa Harber-Aschan, Billy Gazard, David Chandran, Jyoti Sanyal, Stephani Hatch, Anna Kolliakou, Jayati Das-Munshi

**Affiliations:** 1 Institute of Psychiatry, Psychology and Neuroscience, King’s College London, London, UK; 2 Department of Computer Science, University of Sheffield, Sheffield, UK; 3 South London and Maudsley NHS Foundation Trust, London, UK; 4 King’s College Hospital NHS Trust, London, UK; 5 Economic and Social Research Council (ESRC) Centre for Society and Mental Health, King’s College London, London, UK

**Keywords:** mental health, health informatics, epidemiology, adult psychiatry

## Abstract

**Objectives:**

We set out to develop, evaluate and implement a novel application using natural language processing to text mine occupations from the free-text of psychiatric clinical notes.

**Design:**

Development and validation of a natural language processing application using General Architecture for Text Engineering software to extract occupations from de-identified clinical records.

**Setting and participants:**

Electronic health records from a large secondary mental healthcare provider in south London, accessed through the Clinical Record Interactive Search platform. The text mining application was run over the free-text fields in the electronic health records of 341 720 patients (all aged ≥16 years).

**Outcomes:**

Precision and recall estimates of the application performance; occupation retrieval using the application compared with structured fields; most common patient occupations; and analysis of key sociodemographic and clinical indicators for occupation recording.

**Results:**

Using the structured fields alone, only 14% of patients had occupation recorded. By implementing the text mining application in addition to the structured fields, occupations were identified in 57% of patients. The application performed on gold-standard human-annotated clinical text at a precision level of 0.79 and recall level of 0.77. The most common patient occupations recorded were ‘student’ and ‘unemployed’. Patients with more service contact were more likely to have an occupation recorded, as were patients of a male gender, older age and those living in areas of lower deprivation.

**Conclusion:**

This is the first time a natural language processing application has been used to successfully derive patient-level occupations from the free-text of electronic mental health records, performing with good levels of precision and recall, and applied at scale. This may be used to inform clinical studies relating to the broader social determinants of health using electronic health records.

**Strengths and limitations of this study**

- The application was developed on a sizeable corpus of training and test data from a large routine dataset, which was applied at scale over the record, providing us with insights into the occupations of patients using secondary mental health services.
- The application was thoroughly evaluated using gold-standard and cross-checking strategies.
- The application was developed and tested in a single site electronic health record system in the UK—the application will require validation on other similar systems before using them.
- The application does not identify the temporality of occupations; it is unclear whether the extracted occupations are currently or previously held by the patient.
- The application cannot yet identify where a patient holds a health or social care occupation as these occupations could not be ascertained with confidence.

## Introduction

Occupation and mental illness are highly inter-related. There are long-standing concerns that unemployment rates are considerably higher for people with mental illness,^1 2^ and work participation has been described as among the most important factors for recovery by clinicians and service users alike.^3 4^ People with mental illnesses may also undertake precarious, poorly paid work which could have further negative impacts on mental health.^5^ Moreover, occupation is a fundamental individual-level indicator of socioeconomic position as it is predictive of material resources and is indicative of wider class interactions.^6^ Recent systematic reviews have called for large and detailed longitudinal studies to investigate predictors of occupational functioning, and to examine how and when occupation is associated with clinical outcomes in mental health cohorts, as this is currently poorly understood.^7 8^

Research using electronic health records (EHRs) allows for the large-scale collection of sociodemographic and clinical information which would otherwise be logistically challenging to collect using traditional epidemiological approaches.^9^ However, EHR research has major limitations including that information relating to occupation is either not recorded routinely or is poorly captured within standard EHR systems.^10^ As there are no existing methods, to our knowledge, to reliably extract occupations from the psychiatric EHR, this is a problematic barrier for desirable research where occupation is an indicator of socioeconomic status and in research examining the relationships between occupation, mental illness and recovery.

Patient information can be recorded in the structured fields of the EHR, where the clinician records categorical or numerical data. In many psychiatric EHR systems, patient information is recorded in narrative text sections of the record, known as the ‘free-text’ fields, for example in notes describing patient contact.^11^ Information recorded in this way is harder to extract. Clinicians may only record the patient’s occupation in such free-text fields and not the structured fields, making it more complicated, time-consuming and labour intensive to identify the patient’s occupation.^10^ Natural language processing (NLP) methods have the potential to overcome this obstacle by applying algorithms to extract relevant textual information. NLP methods have previously been used successfully for text mining from mental health EHRs, for example, to identify smoking status and symptoms of severe mental illness,^12–16^ and other types of clinical records.^17 18^ NLP methods are also being applied in large-scale industrial and occupational research.^19–21^

This paper traces the development of a novel application using NLP methods to extract patient occupations from the free-text of EHRs from a large mental health Trust in south London, UK. We then provide profile information on the most frequently extracted occupations for patients using secondary mental health services, and clinical and sociodemographic factors associated with recorded occupation data compared with missing occupation data.

## Materials and methods

### Setting

Data for the development of the application were obtained from the South London and Maudsley (SLaM) Biomedical Research Centre (BRC) case register: a repository of de-identified clinical data from the EHRs of individuals receiving care from SLaM secondary mental health services. SLaM covers a socially and ethnically diverse inner-city area of approximately 1.3 million people.^22^ The register contains over 350 000 de-identified patient records which are available for research purposes through the Clinical Record Interactive Search (CRIS) platform. CRIS was developed at SLaM in 2008 and similar resources have subsequently been implemented at several other mental health Trusts in the UK. The present application was developed over the years 2017–2019 and was implemented in January 2020.

### Datasets

**Figure 1:**
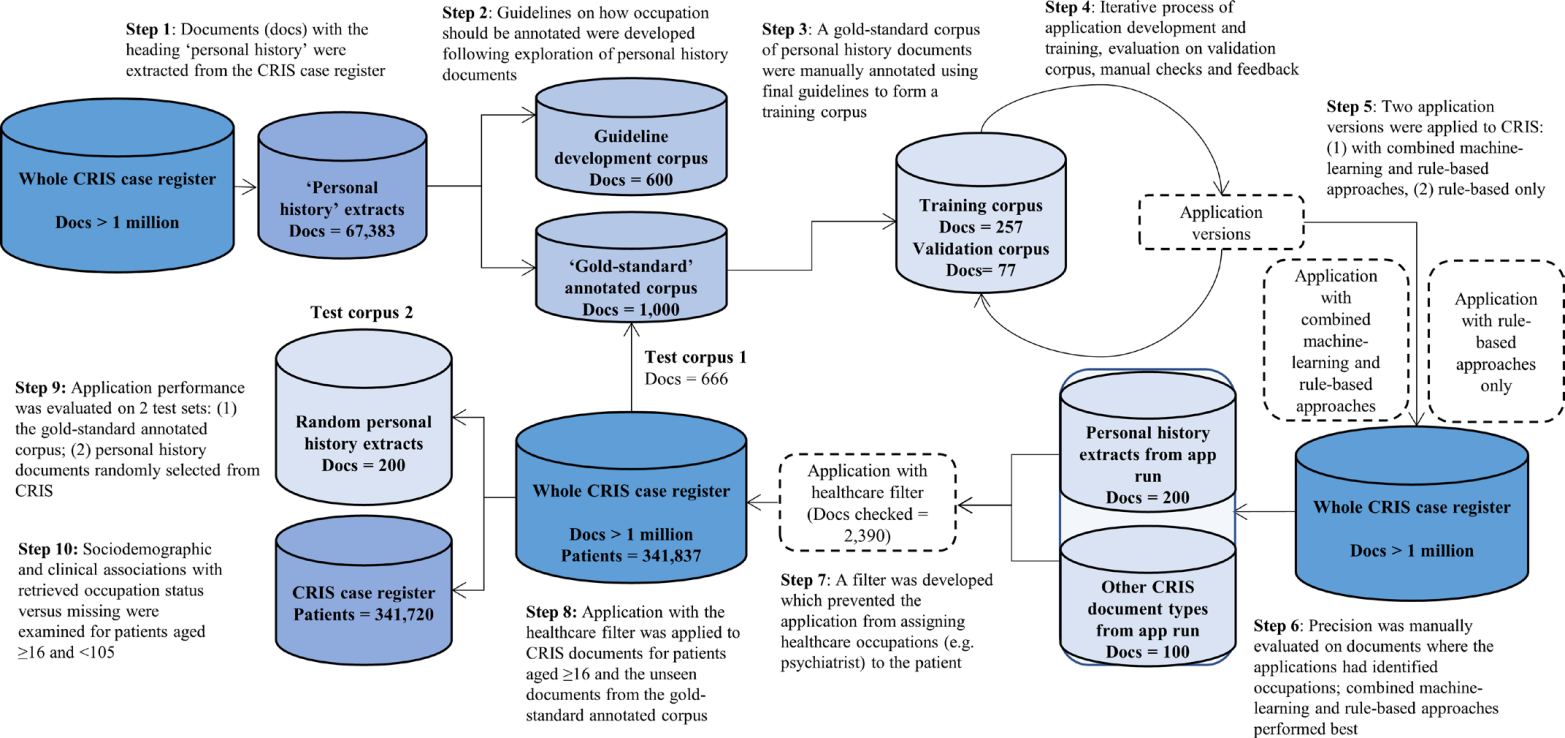
A step-by-step illustration of the methods used for the occupation application development and evaluation, with the number and types of documents used at each step. CRIS, Clinical Record Interactive Search.

Figure 1 describes how the CRIS-derived dataset was used for cycles of application development and evaluation, and summarises the key steps taken. Age restrictions were implemented throughout document selection: free-text documents were only extracted where the patient was aged 16 years and above at time of document extraction. There were no date restrictions. Free-text documents were retrieved from several different sections in this EHR, for example, sections for clinical risk assessments and separate sections for discharge summaries. Further detail on the types of documents used at each stage of application development can be found in online supplemental file 1.

10.1136/bmjopen-2020-042274.supp1 Supplementary data

### Developing, evaluating and implementing the application

#### Manually annotating occupation in the free-text

Personal history sections of psychiatric assessments typically describe the patient’s occupation, as well as education and family history. Personal history sections of documents were extracted from the free-text fields of records at the document level using an NLP application (precision=0.78, recall=0.88) developed by DC (N=67 383). Typically these extracts were derived from documents of the ‘attachments’ type, which is a word-processed document such as a letter to or from the patient’s primary care physician; and ‘events’, which are short pieces of text used to record some detail of a clinical encounter.

Occupations were identified in personal history documents by an interdisciplinary team of trained researchers, including clinicians, bioinformaticians and mental health researchers. In common with the NLP community, we refer to this task of marking mentions of occupation text as annotation. A set of occupation annotation guidelines was developed through an iterative process of manual annotation practice, team discussions and agreed annotation rulemaking (online supplemental file 2). These guidelines specified when and how an occupation should be identified, annotated and extracted from the text. An occupation annotation was defined as having two parts. First, the *occupation* itself was annotated. This could be an occupation title, for example, a ‘builder’; or an occupation description, for example, ‘construction’. Second, the occupation *relation* was specified: who the occupation belongs to, for example, the patient or their family member. Temporality, including when or how long a patient has held an occupation, was not annotated as the text often did not state this consistently. In total, 600 personal history documents were manually annotated to practise annotating occupation from text and develop the annotation guidelines (ET, AK, SM, KB, ZC, AR). Once the guidelines were developed, a set of 1000 personal history documents were manually annotated on the General Architecture for Text Engineering (GATE) platform^23^ using the guidelines to create a gold standard, where 200 were double annotated to evaluate inter-annotator reliability.

10.1136/bmjopen-2020-042274.supp2 Supplementary data

#### Application development

Out of the 1000 gold-standard annotated personal history documents, 334 documents were reserved for application development. The application was developed by XS on the GATE platform,^23^ a widely used NLP framework with over 40 000 downloads per version and a history of use in the UK National Health Service (NHS), among other sectors.^17^ The application was trained on 257 of the gold-standard annotated documents. To check the performance of the application throughout development, precision and recall metrics were estimated using a customised performance tool developed by XS on GATE on a validation set of 77 gold-standard annotated documents, with a total of 405 occupation annotations. Precision was the proportion of occupations correctly annotated, to all occupations annotated (whether correct or incorrect). Recall was the proportion of occupations correctly annotated, to all occupations that could have been correctly annotated. The application outputs were manually checked by the Clinical Informatics Interface and Network Lead at the National Institute for Health Research BRC (AK). Any problems identified were addressed in each version of the application. An iterative process of application development, training, evaluation of performance using GATE and manual checks was repeated 10 times, at which point the application reached a good level of performance on the validation set.

#### Machine-learning approach testing

**Figure 2:**
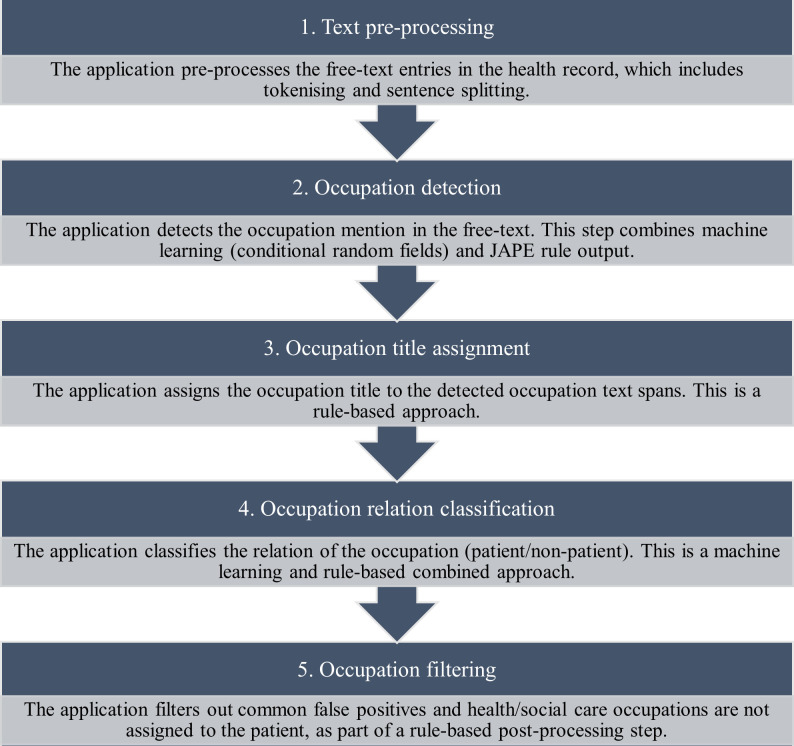
The process undertaken by the occupation application when text mining occupations from the clinical free-text field.

Two early versions of the application were developed for testing over unannotated documents in the CRIS case register: one version used combined machine-learning and rule-based approaches, and the second version used rule-based approaches only. This was due to a concern that the application had therein been developed on limited training data, and the trained model may not generalise well on free-text other than personal history documents, which could lead to a loss in precision when implemented over the EHR. Specifically, the machine-learning approaches involved a trained conditional random field classifier to identify occupation mentions in the text, and a support-vector machine-based classifier to identify the occupation relation. Figure 2 illustrates how the machine-learning and rule-based approaches were used in combination; this is described in further technical detail in online supplemental file 3.

10.1136/bmjopen-2020-042274.supp3 Supplementary data

Two researchers (NC, AK) manually calculated precision performance for both versions of the application on 100 personal history documents (in domain testing data) and 100 other free-text document types (out domain test data) which had at least one occupation extraction and were previously unseen by the application in development. While both application versions performed well when text mining occupations from these test sets (precision ≥0.79, further detail in online supplemental file 3), the application with machine-learning approaches performed at the highest level of precision when assigning the occupation relation. The research team concluded from this testing phase that the application with combined machine-learning and rule-based approaches was most appropriate, as this pipeline performed best at assigning the occupation relation.

#### The healthcare occupation filter

The evaluation of the application performance over CRIS documents revealed that the most common false positives were extractions where the healthcare professional involved in the patient’s care was incorrectly annotated as the patient’s occupation (96% of annotations manually checked were health/social care occupations). To deal with this issue, health and social care occupations were added to a filter. The application then implemented a rules-based step where the filtered healthcare occupations were prevented from being annotated as belonging to the patient. Occupations added to this filter included variations on terms for psychiatrists and doctors, therapists, nurses and social workers, following the checking of 2390 documents to confirm that these were common false positives.

#### Application implementation and testing

The final version of the text mining application with the healthcare filter applied was run over 10 free-text fields, including those where personal history sections were found, in the records of all patients on the CRIS case register aged 16 years and above. The fields included sections of the record such as discharge summaries, attachments, events and risk assessments (more detail in online supplemental file 1). The application was evaluated on a total of 866 documents: 666 gold-standard annotated personal history documents (test corpus 1), and 200 previously unannotated random personal history documents from the CRIS dataset at the time of the application run (test corpus 2). Test corpus 1 was evaluated on GATE, and test corpus 2 was manually checked for occupations and then cross-referenced with the application output. The performance metrics considered the precision and recall level for the annotations made by the application, where both the occupation annotation and the relation classification needed to be accurate to be considered a ‘true positive’. It was not feasible in this study to randomly select non-personal history documents for evaluation as patient occupations were rarely mentioned in the record compared with other information (eg, medication). As the application extracted an annotation entitled ‘other’, 200 of these annotations were manually checked for precision to further investigate these instances where the application was unable to assign an occupation title.

The EHR in the present study contains a structured field to record occupation: the ‘Employment-ID’. This was explored on the CRIS platform using SQL queries. The proportion of completed ‘Employment-IDs’ from the records of all patients over the age of 16 years in January 2020 was extracted. The text mining application was simultaneously run over clinical records through CRIS, and the extracted patient occupations were converted into an SQL table. Sociodemographic, clinical and service contact data were also extracted from the structured fields of records using SQL queries. Data were exported to and analysed in STATA V.15 to examine predictors of occupational data extraction using logistic regression models. This included the patient’s age at time of occupation extraction, gender, marital status, ethnicity, Index of Multiple Deprivation (IMD) score and primary diagnosis. Indicators of service contact included number of events in the record, number of face-to-face events in the record, number of spaces in the free-text fields of the record (as a proxy for word count), number of active days under SLaM services and number of inpatient bed-days. These variables were transformed into categories, for example, IMD scores were categorised into quartiles of local neighbourhood deprivation. Where data were missing for the extracted variables, this was coded as a ‘not known’ category for each variable.

Logistic regression models examined crude associations between the sociodemographic, clinical and service contact variables (predictors), and the recording of at least one patient occupation (outcome) from either the structured or free-text fields. The null hypothesis was that none of the predictors would be associated with occupation recording. First, models were adjusted for amount of contact the patient had with services. Fully adjusted models accounted for all other sociodemographic and clinical variables. Across all models, likelihood ratio tests were conducted to test the overall association between the variable and occupation recording. The aim of this analysis was to ascertain the characteristics of patients who had occupation recorded in their health record.

### Patient and public involvement

The proposal for this study was reviewed and approved by the patient-led CRIS oversight committee prior to the commencement of the project. No other consultations were made with patients or the public during the process of the study.

## Results

### Annotating occupation

When double annotating 200 personal history documents, two annotators reached a Cohen’s kappa agreement^24^ of 0.77 for occupation title annotations and 0.72 for occupation relation annotations. Disagreements between annotators included instances where sentences posed unclear or vague references to occupation: for example, in the sentence, ‘she did several things, such as cleaning, cooking’, it was not clear whether these were domestic tasks or occupation descriptions, demonstrating the complexity of annotating occupation from text. Nonetheless, the Cohen’s kappa agreement suggested that occupation could be annotated reasonably consistently across annotators using the annotation guidelines.

### Application development

The application reached a precision level of 0.88 and a recall level of 0.90 on the validation set of documents (N=77). The developed application process with combined rule-based and machine-learning approaches is described in figure 2.

### Application performance

When applied to the gold-standard annotated personal history documents (test corpus 1) on GATE, the application performed at a precision level of 0.79 and a recall level of 0.77. Two-hundred personal history documents were manually checked for occupations and then cross-referenced with the application output (test corpus 2): when considering patient occupations only, the application reached a precision level of 0.77 and recall level of 0.79. An extraction of ‘other’ as an occupational category was excluded from subsequent analysis, as the check of 200 annotations showed that this annotation only reached a precision level of 0.23 and often referenced job-seeking or non-work behaviours, for example, ‘working on his anxiety’.

### Application implementation

**Figure 3:**
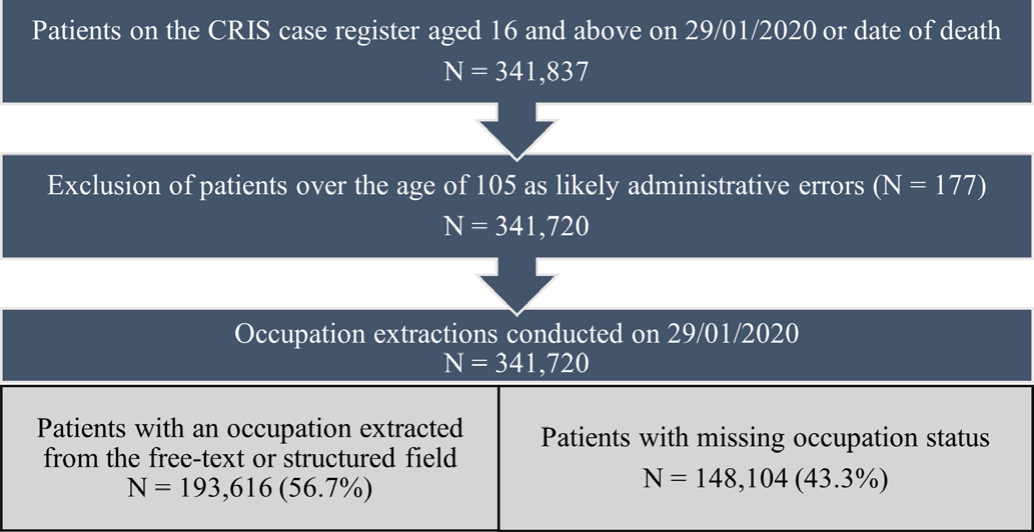
The study population selection and extraction results from text mining occupations from the Clinical Record Interactive Search (CRIS) case register.

Figure 3 shows the study population selection process for the implementation of the application over the CRIS case register, leading to an overall sample size of 341 720 patients.

### Descriptives

The demographics of the study population at the time of occupation extraction are described in table 1, as well as patient diagnostic categories and two indicators of the amount of service contact the patient has had: the number of ‘events’ entries added to the EHR and number of inpatient bed-days. The three other extracted indicators for service contact (number of ‘face-to-face events’, total active days under SLaM mental health services and number of spaces in the text in the record) were excluded from analysis due to collinearity with the ‘events’ variable.

**Table 1:** Sociodemographic and clinical features of the Clinical Record Interactive Search case register*

	Number of patients (%) (total N=341 720)
Age	
16–29	84 181 (24.63)
30–49	123 216 (36.06)
50–69	79 880 (23.38)
70–89	43 852 (12.83)
90+	10 591 (3.10)
Gender	
Male	166 480 (48.72)
Female	175 007 (51.21)
Other/not known	233 (0.07)
Ethnicity	
White British	136 289 (39.88)
Irish	5182 (1.70)
Black Caribbean	34 229 (10.02)
Black African	15 654 (4.58)
Indian	4345 (1.27)
Pakistani	1852 (0.54)
Bangladeshi	1088 (0.32)
Chinese	1124 (0.33)
Other Asian	5500 (1.61)
Other ethnic group	19 650 (5.75)
Other white	22 076 (6.46)
Mixed	1879 (0.55)
Not known	92 222 (26.99)
Marital status	
Married/civil partnership/cohabiting	46 617 (13.64)
Divorced/separated/civil partnership dissolved	17 309 (5.07)
Widowed	15 758 (4.61)
Single	141 111 (41.29)
Not known	120 925 (35.39)
Local quartiles of neighbourhood deprivation	
Least deprived	79 537 (23.28)
3rd quartile	80 049 (23.43)
2nd quartile	79 767 (23.34)
Most deprived	79 829 (23.36)
Address not known	22 538 (6.60)
Primary diagnosis	
F30–F39: mood (affective) disorders	37 796 (11.06)
F00–F09: organic, including symptomatic, mental disorders	29 801 (8.72)
F10–F19: mental and behavioural disorders due to psychoactive substance misuse	27 870 (8.16)
F20–F29: schizophrenia, schizotypal and delusional disorders	18 253 (5.34)
F40–F49: neurotic, stress-related and somatoform disorders	31 962 (9.35)
F50–F59: behavioural syndromes associated with physiological disturbances and physical factors	9166 (2.68)
F60–F69: disorders of adult personality and behaviour	6605 (1.93)
F70–F79: mental retardation	2732 (0.80)
F80–F89: disorders of psychological development	5874 (1.72)
F90–F98: behavioural and emotional disorders with onset usually occurring in childhood and adolescence	12 028 (3.52)
Other diagnosis	83 847 (24.54)
Not known	75 786 (22.18)
Quartiles of ‘events’ entered into the health record	
No events	50 673 (14.83)
Least events (1–3)	86 818 (25.41)
2nd quartile (4–10)	62 804 (18.38)
3rd quartile (11–40)	68 774 (20.13)
Most events (41+)	72 651 (21.26)
Inpatient bed-days	
No inpatient admissions	311 099 (91.04)
Low (1–2)	1937 (0.50)
Moderate (3–31)	10 587 (3,10)
High (32+)	18 337 (5.37)

*At the time of the occupation application run (29 January 2020).

### Occupation extractions

The structured field for employment was populated for 46 705 (13.7%) patients. Prior to the implementation of the healthcare filter, 81.5% patients had at least one patient-occupation extraction. When using the final version of application to extract occupations from the free-text fields with the healthcare filter applied, this recalled at least one patient-related occupation for 184 521 patients (54.0%). By combining structured field and text mined occupations, patient-related occupations were retrieved for 193 616 patients (56.7%).

The structured field for occupation included 13 categories for occupational status, for example, ‘unemployed’ or ‘paid employment’. In contrast, the text mining application retrieved 72 955 different patient-related occupation types. In total, there were 3 957 959 patient-related occupation extractions. Multiple occupation types were often extracted per patient (median=4, IQR=6).

The top five extracted occupations across the total sample of 341 720 patients were: student (n=98 719, 28.9%), unemployed (n=97 809, 28.6%), carer (n=61 893, 18.1%), self-employed (n=36 506, 10.8%) and retired (n=33 518, 9.8%). The less frequent extractions tended to be more specific occupation types, for example, ‘retail worker’ and ‘banker’. The application also extracted undocumented ways of making money, including ‘drug dealer’ and ‘sex worker’.

### Associations with occupation recording

Patients were split into binary groups: those who had an occupation recorded either in the structured field or free-text (n=193 616, 56.7%), and patients who did not have occupation recorded, that is, missing occupational data (n=148 104, 43.3%). Logistic regressions were used to examine sociodemographic, clinical and service contact associations with recorded occupations (table 2).

**Table 2:** Results from crude and multivariable logistic regression analyses examining predictors of occupation recording from the Clinical Record Interactive Search case register*

	N (%) with at least one occupation retrieved by structured field/text mining extractions	OR (95% CI)	aOR† (95% CI)	aOR‡ (95% CI)
Age				
16–29	41 653 (49.48)	Reference	Reference	Reference
30–49	68 422 (55.53)	1.27 (1.25 to 1.30)	1.56 (1.53 to 1.59)	1.72 (1.68 to 1.75)
50–69	49 289 (61.70)	1.65 (1.61 to 1.68)	1.98 (1.93 to 2.02)	2.19 (2.14 to 2.25)
70–89	27 175 (61.97)	1.66 (1.63 to 1.70)	1.71 (1.67 to 1.76)	1.60 (1.54 to 1.65)
90+	7077 (66.82)	2.06 (1.97 to 2.15)	2.14 (2.04 to 2.24)	2.00 (1.89 to 2.11)
Gender				
Male	96 141 (57.75)	Reference	Reference	Reference
Female	97 443 (55.68)	0.92 (0.91 to 0.93)	0.88 (0.87 to 0.90)	0.87 (0.85 to 0.88)
Other/not known	32 (13.73)	0.12 (0.08 to 0.17)	0.10 (0.07 to 0.15)	0.16 (0.10 to 0.24)
Ethnicity				
White British	91 575 (67.19)	Reference	Reference	Reference
Irish	4303 (74.04)	1.39 (1.31 to 1.48)	1.24 (1.17 to 1.33)	1.23 (1.15 to 1.31)
Black Caribbean	24 753 (72.32)	1.28 (1.24 to 1.31)	0.99 (0.96 to 1.02)	1.06 (1.03 to 1.09)
Black African	11 341 (72.45)	1.28 (1.24 to 1.33)	1.07 (1.03 to 1.11)	1.12 (1.07 to 1.17)
Indian	2876 (66.19)	0.96 (0.90 to 1.02)	0.91 (0.85 to 0.97)	0.91 (0.85 to 0.98)
Pakistani	1185 (63.98)	0.87 (0.79 to 0.95)	0.81 (0.73 to 0.90)	0.82 (0.74 to 0.91)
Bangladeshi	719 (66.08)	0.95 (0.84 to 1.08)	0.90 (0.78 to 1.03)	0.94 (0.82 to 1.08)
Chinese	690 (61.39)	0.78 (0.69 to 0.88)	0.73 (0.65 to 0.84)	0.81 (0.71 to 0.92)
Other Asian	3543 (64.42)	0.88 (0.84 to 0.94)	0.82 (0.78 to 0.87)	0.85 (0.80 to 0.91)
Other ethnic group	11 768 (59.89)	0.73 (0.71 to 0.75)	0.77 (0.75 to 0.80)	0.75 (0.72 to 0.77)
Other white	14 610 (66.18)	0.96 (0.93 to 0.98)	0.94 (0.91 to 0.97)	0.97 (0.94 to 1.00)
Mixed race	1197 (63.70)	0.86 (0.78 to 0.94)	0.68 (0.61 to 0.75)	0.78 (0.70 to 0.87)
Not known	25 056 (27.17)	0.18 (0.18 to 0.19)	0.31 (0.31 to 0.32)	0.50 (0.49 to 0.51)
Marital status				
Married/civil partnership/cohabiting	31 037 (66.58)	Reference	Reference	Reference
Divorced/separated/civil partnership dissolved	13 346 (77.10)	1.69 (1.62 to 1.76)	1.47 (1.40 to 1.53)	1.41 (1.35 to 1.47)
Widowed	11 309 (71.77)	1.28 (1.23 to 1.33)	1.05 (1.00 to 1.09)	1.05 (1.01 to 1.10)
Single	98 841 (70.04)	1.17 (1.15 to 1.20)	1.02 (1.00 to 1.05)	1.24 (1.21 to 1.27)
Not known	39 083 (32.32)	0.24 (0.23 to 0.25)	0.33 (0.32 to 0.33)	0.49 (0.47 to 0.50)
Local quartiles of neighbourhood deprivation				
Least deprived	48 155 (60.54)		Reference	Reference
3rd quartile	47 583 (59.44)	0.96 (0.94 to 0.97)	0.97 (0.95 to 0.99)	0.96 (0.94 to 0.99)
2nd quartile	45 842 (57.47)	0.88 (0.86 to 0.90)	0.94 (0.91 to 0.96)	0.93 (0.91 to 0.95)
Most deprived	41 800 (52.36)	0.72 (0.70 to 0.73)	0.89 (0.87 to 0.91)	0.88 (0.86 to 0.90)
Address not known	10 236 (45.42)	0.54 (0.53 to 0.56)	0.70 (0.67 to 0.72)	0.77 (0.74 to 0.80)
Diagnosis				
F30–F39: mood (affective) disorders	27 057 (71.59)	Reference	Reference	Reference
F00–F09: organic, including symptomatic, mental disorders	20 269 (68.01)	0.84 (0.82 to 0.87)	0.91 (0.88 to 0.94)	0.71 (0.68 to 0.74)
F10–F19: mental and behavioural disorders due to psychoactive substance misuse	18 150 (65.12)	0.74 (0.72 to 0.77)	0.71 (0.68 to 0.73)	0.47 (0.45 to 0.49)
F20–F29: schizophrenia, schizotypal and delusional disorders	14 645 (80.23)	1.61 (1.54 to 1.68)	0.87 (0.83 to 0.91)	0.78 (0.74 to 0.82)
F40–F49: neurotic, stress-related and somatoform disorders	19 920 (62.32)	0.66 (0.64 to 0.68)	0.75 (0.72 to 0.77)	0.76 (0.73 to 0.79)
F50–F59: behavioural syndromes associated with physiological disturbances and physical factors	5287 (57.68)	0.54 (0.52 to 0.57)	0.65 (0.62 to 0.68)	0.68 (0.64 to 0.72)
F60–F69: disorders of adult personality and behaviour	4739 (71.75)	1.01 (0.95 to 1.07)	0.68 (0.64 to 0.73)	0.77 (0.72 to 0.82)
F70–F79: mental retardation	2277 (83.35)	1.99 (1.79 to 2.20)	1.81 (1.63 to 2.03)	1.69 (1.51 to 1.90)
F80–F89: disorders of psychological development	4377 (74.78)	1.16 (1.09 to 1.24)	1.22 (1.14 to 1.30)	1.78 (1.66 to 1.92)
F90–F98: behavioural and emotional disorders with onset usually occurring in childhood and adolescence	8754 (72.78)	1.06 (1.01 to 1.11)	1.25 (1.19 to 1.32)	1.84 (1.74 to 1.93)
Other diagnosis	43 787 (52.22)	0.43 (0.42 to 0.45)	(0.68 to 0.72)	0.76 (0.73 to 0.78)
Not known	24 354 (32.14)	0.19 (0.18 to 0.19)	0.44 (0.43 to 0.45)	0.66 (0.64 to 0.68)
Quartiles of ‘events’ entered into the health record				
No events	12 012 (23.70)	Reference	Reference	Reference
Least events	35 009 (40.32)	2.17 (2.12 to 2.23)	2.18 (2.13 to 2.23)	1.75 (1.70 to 1.79)
2nd quartile	34 368 (54.72)	3.89 (3.79 to 3.99)	3.89 (3.79 to 3.99)	2.79 (2.71 to 2.87)
3rd quartile	49 237 (71.59)	8.11 (7.90 to 8.33)	8.06 (7.85 to 8.28)	5.01 (4.86 to 5.16)
Most events	62 990 (86.70)	20.98 (20.37 to 21.60)	18.89 (18.29 to 19.50)	9.77 (9.43 to 10.1)
Inpatient bed-days				
No inpatient admissions	167 213 (53.75)	Reference	Reference	Reference
Low (1–2)	1408 (82.97)	4.19 (3.69 to 4.76)	1.87 (1.64 to 2.14)	1.68 (1.47 to 1.93)
Moderate (3–31)	8714 (82.31)	4 (3.81 to 4.21)	1.06 (1.00 to 1.11)	1.01 (0.95 to 1.07)
High (32+)	16 281 (88.79)	6.81 (6.51 to 7.14)	1.57 (1.49 to 1.66)	1.32 (1.25 to 1.39)

*All variables listed in this table had a strong association with the outcome variable (p<0.0001), assessed by likelihood ratio tests.

†Adjusted for service contact variables (number of events and inpatient bed-days).

‡Adjusted for all other variables in the table.

aOR, adjusted OR.

Across all models, all predictors were strongly associated with a recording of occupation even after fully adjusting for all other variables (likelihood ratio tests p<0.0001). When key sociodemographic data were missing from the record, the odds of occupational data being recorded decreased: for example, where the marital status of the patient was ‘not known’, the fully adjusted OR for a recording of an occupation was 0.49 (95% CI 0.47 to 0.50) compared with patients who were recorded as married/in a civil partnership/cohabiting. Female patients were significantly less likely to have occupation recorded compared with male patients, and older patients were most likely to have occupational data recorded compared with the youngest patients. Compared with patients of white British ethnicity, patients of Irish, black Caribbean or black African ethnicity were more likely to have an occupation recorded; while Indian, Pakistani, Chinese, mixed race or patients recorded as being from ‘other’ Asian or ethnic groups were less likely to have occupation recorded. The odds of having occupation recorded were significantly lower for patients who were living in the most deprived local areas compared with the most affluent areas. Generally, patients with a primary diagnosis of an affective disorder had a higher odds of an occupation extraction than patients with other diagnoses, including organic disorders. In the crude logistic regression models, patients diagnosed with schizophrenia, schizotypal or delusional disorders were more likely to have occupation extracted (OR 1.61, 95% CI 1.54 to 1.68). However, once adjusting for amount of contact with services, these patients were significantly less likely to have occupation extracted compared with patients with affective disorders (adjusted OR 0.87, 95% CI 0.83 to 0.91).

## Discussion

Annotating and extracting occupation from the free-text fields in clinical records are challenging tasks. We have developed a tool to text mine patient occupations with a good degree of confidence from a mental health EHR, and applied this at scale over a large EHR in south London. An important finding was that we could retrieve over double the number of patient occupations using text mining methodology than when using pre-existing structured fields alone. We could also access a much wider diversity of occupation types: this further detail on occupations held by patients opens up the possibility for the translation of occupations onto social class schema, which would not have been possible with the limited structured field categories. The most prevalent patient occupations were ‘student’ and ‘unemployed’. There were differences between patients who had occupation recorded and patients whose occupation data remained missing: patients with occupations recorded were more likely to be of an older age, male, divorced/separated, living in areas of lower deprivation and have more contact with mental health services. Across ethnic minority groups, there were mixed findings relating to the recording of occupation. Compared with white British patients, Irish, black Caribbean and black African patients were slightly more likely to have a recording of occupation, whereas all other ethnic minority groups were less likely to have a recording. Although it is possible that some of the demographic associations with the recording of occupation in the case notes were impacted by residual confounding in adjusted models, these findings may also indicate disparities relating to how occupations are assessed and recorded in the clinical record and should be explored in future work, particularly given the strong correlation of employment with recovery within the context of mental disorders.

This study broadly supports the work of other studies which indicate that clinicians mostly describe occupation in the free-text of EHR systems, when these are available, rather than structured fields.^10^ This study is the first of its kind to text mine patient occupations from a mental healthcare EHRs. There have been several previous efforts to extract patient occupations from other healthcare free-text notes. Occupations have been text mined from general medical clinical text; however, in these studies the algorithms reached low levels of performance, largely due to a lack of training data.^25 26^ Dehghan and colleagues text mined occupation from the clinical records of patients with cancer in the UK, reaching similar precision and recall levels to the present study.^27^ However, none of these applications distinguished between text mining occupations belonging to the patient and other relations, had the scope of applying and testing the text mining methodology at scale across the EHR or examined associations with extracted versus missing occupational data. The present application therefore represents significant progress in our ability to text mine patient occupations from the EHR and furthers our understanding of what this may mean in practice.

We found that text mining greatly increased our retrieval of patient occupations in this psychiatry EHR database. Psychiatric notes may be more detailed than other types of healthcare text (for example, in general medicine) when describing the patient’s occupation, as this often forms part of psychiatric history taking and assessment. We found that a sizeable proportion of patients over CRIS have at some point been a student or unemployed. A separate NLP application being developed using CRIS data (by author JS) will be able to interrogate this student group further by extracting the patient’s level of educational attainment, which will complement the present application. There is also scope to explore older groups of patients who are students but are also working using this methodology. Our finding that unemployment was a dominant occupational category is consistent with previous research, in that unemployment levels are elevated particularly for those with severe mental illnesses compared with the general population.^1 2^ It may also be the case that some patients in this group are formally unemployed but are working in more informal, undocumented ways to make money. This application identified some informal occupations, which provides interesting avenues for further research.

One limitation of our approach is that we could not distinguish the temporality of occupations—whether they were currently or previously held by the patient. While developing the annotation guidelines, we found that the text was unlikely to be sufficient to assess temporality, as it was often not explicitly stated when the patient started or left an occupation, or how long they have held a position for. Multiple occupations were often extracted for a single patient, adding to the complexity. While there is work ongoing to use NLP to detect temporality in psychiatric healthcare text,^28^ this remains a challenge and is a potential avenue for further work that is beyond the scope of this study. As this application was developed at a single site in the UK, the generalisability of the application may be reduced, first to text in the English language and second to this catchment area. As it was not possible to assign health and social care occupations to patients with reasonable confidence, we will also be missing patients who hold these occupations; however, we are planning further work to develop this aspect of the application. Notwithstanding these limitations, this application was developed through an extensive process of training and testing using a large corpus leading to the application of text mining algorithms for occupation at scale. This methodology is already revealing the kinds of occupations held by patients using secondary mental health services.

The development of this application has numerous implications. First, this application will be valuable in allowing researchers to examine relationships between occupation and health in large psychiatric case registers. For example, work is currently underway using this application to investigate predictors of unemployment in a cohort of patients with severe mental illness.^29^ As CRIS-like systems are in use over several sites in the UK, there is the scope to test and implement this application in other mental healthcare providers using similar EHR platforms. This application could also have potential practical implications including identifying unemployed patients to target interventions such as Individual Placement and Support and retrieving occupational distributions for audits and organisational monitoring in NHS mental health Trusts. Lastly, this application may have implications beyond mental health research and text, notably in research on industrial injuries, although this requires further testing.

There is room for further progress in this application as the NLP field further develops, including identifying the temporality of occupations and improving relation classification for health and social care occupations. We plan to develop methodology to ascertain the occupational social class of patients, using the large diversity of occupations extracted, to further inform health inequalities research specific to mental health. Future studies implementing this application in other CRIS systems may be able to investigate the transferability of the application to other NHS sites in the UK that serve different patient populations. Overall, we hope that this approach will prove useful in addressing our understanding of the interactions between occupation and health in those with mental illness.

## Supplementary Material

Reviewer comments

Author's manuscript
