# Supplementary material for: Text mining occupations from the mental health electronic health record: a natural language processing approach using records from the Clinical Record Interactive Search (CRIS) platform in south London, UK
Source: BMJ Open. 2021 Mar 25;11(3):e042274. doi: 10.1136/bmjopen-2020-042274 (PMC7996661; doi:10.1136/bmjopen-2020-042274)
Supplement: Supplementary data [file bmjopen-2020-042274supp001.pdf]

Supplementary File 1: Descriptions of the datasets used in the development, testing and implementation of the occupation application

**Application Development and Testing Datasets**			
	**Type of document**	**Document count**	**No. of Occupation Annotations (manual)**
Validation corpus	Personal history	77<br>(+256 documents used in training)	405
Testing corpus 1: with vs without machine-learning comparison	Personal history + other CRIS documents	200	521
Testing corpus 2: gold-standard annotated documents	Personal history	666	3,429
Testing corpus 4: Unannotated documents	Personal history	200	442
**Application Implementation Dataset**			
	Type of document	**Patient count**	**No. Of Occupation Extractions (application)**
CRIS case register of patient records aged >=16	Attachments	341,720	21,321,757 (all relations)
	Events		
	Correspondence		
	Discharge Notification Summaries		
	History		

	Mental State Formulations		
	Presenting Circumstances		
	Risk Events		
	Social Situation		
	Ward Progress Notes		

Table 1: Descriptions of the datasets used in the development, testing and implementation of the occupation application
