# Supplementary material for: Text mining occupations from the mental health electronic health record: a natural language processing approach using records from the Clinical Record Interactive Search (CRIS) platform in south London, UK
Source: BMJ Open. 2021 Mar 25;11(3):e042274. doi: 10.1136/bmjopen-2020-042274 (PMC7996661; doi:10.1136/bmjopen-2020-042274)
Supplement: Supplementary data [file bmjopen-2020-042274supp002.pdf]

OCCUPATION ANNOTATION GUIDELINES

Authors:

Background – Natasha Chilman & Esther Tolani

Annotation rules – Esther Tolani, Angus Roberts, Zoe Chui, Karen Birnie, Lisa Harber-Aschan, Billy Gazard, Anna Kolliakou & Jayati Das-Munshi

General Tips – Esther Tolani

Appendices – Natasha Chilman & Anna Kolliakou

With thanks to Shirlee MaCrimmon for annotation support.

Supplementary File 2 to accompany the BMJ Open paper, "Text-mining occupations from the mental health electronic health record: a natural language processing approach using the Clinical Record Interactive Search (CRIS) platform to identify patient occupations in south London, UK."

Background 2
Setting 2
Development 2
Annotation rules 5
Annotation rules 6
Occupation 8
Subject of Occupation 13
Exclusion Criteria 15
General Tips 16
References 17
Appendix 1 17
A timeline of actions leading to guideline and application development 17
Appendix 2 22
Annotation Guidelines Version 1 22

Background

The CRIS-occupation-application has been developed to enable researchers to extract occupations from the Clinical Record Interactive Search (CRIS) case register. When using the occupation application, it is important to consider how it has been trained and tested to allow for appropriate use of the application and accurate interpretation of results. These guidelines provide clear and transparent rules which specify how occupations should be annotated manually in free-text EHRs, which then informed the development of the occupation application, and a gold-standard against which the application was evaluated against.

Setting

These occupation annotation guidelines were developed over the years 2017-2020 for use on psychiatric clinical texted accessed through the Clinical Record Interactive Search (CRIS) application. CRIS is a large de-identified case register of electronic health records, comprising of the Electronic Patient Health Journal notes used in South London and Maudsley NHS Trust (SLaM). SLaM is the largest unit mental health provider of secondary services in Europe, serving 1.3 million people across the London boroughs of Lambeth, Southwark, Lewisham and Croydon. The SLaM CRIS case register stores over 350,000 patient records to date, and encompasses a range of secondary mental health services (including inpatient and community mental health services) [1]. Whilst this annotation guideline was written following the exploration of CRIS text extracts, we also recommend that the guidelines can be used as a starting point when extracting occupations from other CRIS systems and psychiatric Electronic Health Records (EHRs) in the UK.

It is important to remember that EHRs are a secondary routine data resource in research, they are used primarily for a practical purpose by clinicians to document patient-level information. This context should be kept in mind when considering the complexity of annotating occupations.

Development

Here we summarise the actions taken to develop the guidelines and describe how the guidelines have changed over time. This is also detailed further in the development timeline (Appendix 1).

These guidelines were based on the ‘personal history’ sections of the free-text entries. When clinicians use ‘personal history’ as a header in the free-text fields in CRIS, the text which follows typically includes information on the patient’s upbringing and family life,

education and – most importantly for our interests – occupation. Personal history sections were chosen as the best place to start when examining how occupation is described in the free-text fields in CRIS. An application previously developed by Dr David Chandran in the Biomedical Research Centre was used to extract personal history documents from CRIS to develop and test the annotation guidelines. A 'document' is a single section of a free-text field in CRIS, for example a letter attachment or event progress note. One patient may have more than one personal history section in their record.

Initial guidelines were drawn from the exploration of 100 personal history documents and team discussions. From the first draft, the occupation annotation guidelines were developed based on the premise that when an occupation is annotated in the free-text, two components must be specified: the occupation (feature) and subject of the occupation (relation). Occupation is a complex concept and can be written as a job title (e.g. a waiter) or a description of a work activity (e.g. serving tables).

The guidelines were developed through an iterative process of document annotation, team discussions and rule development (Appendix 1). 600 personal history documents were annotated throughout this process which informed and tested the sufficiency of the guidelines to instruct occupation annotation. Out of these 600 documents, 250 personal history documents were double annotated. Inter-annotator agreements were calculated throughout the guideline development stages to assess whether the guidelines were sufficient for occupation to be annotated consistently (Appendix 1). By November 2017, 200 further personal history documents were double-annotated with good inter-rater reliability between two manual annotators, with a Cohen's Kappa statistic of 77% for occupation and 72% for relation. This is considered a good level of agreement. 800 documents were then annotated by a single annotator using the latest guideline and together these formed the 1000 document gold-standard annotated document corpus. This corpus was later used for application development (forming the training corpus).

To demonstrate how the guidelines have changed over time, please see Appendix 2 which shows the Guidelines Version 1 (GV1). When compared to the current guidelines, a significant level of detail has been added since the initial draft. For example, there is now a section the beginning of the guidelines stating which parts of a sentence describing occupation should be annotated. Whilst re-drafting the guidelines, an 'additional information' column was added to give further detail on how the annotation rules work, which researchers found helpful when completing annotations. The later drafts of the guidelines also add a 'blank' annotation rule: if the occupation title can be inferred by the text itself then the occupation feature should be left empty, and the relation was determined by the sentence structure. This was important when later evaluating the application, as a bespoke GATE evaluation package was used to take this rule into account. All changes that were made to the annotation guideline throughout the development process were agreed within the research team.

The following guideline is the final annotation guideline document. Whilst there were some small formatting changes made during application development (Appendix 1), the rules in this guideline were used when annotating the 1000 gold-standard training and testing corpus for the application.

Annotation rules

Esther Tolani, Zoe Chui, Karen Birnie, Angus Roberts, Anna Kolliakou, Jayati Das-Munshi, Robert Stewart

These guidelines outline the process for annotating occupation status in GATE. The term(s) highlighted should be the word(s) in the free text which indicate(s) the occupation of an individual. After reading the free text, annotations should be made on the word(s) which is (are) related to an employment status or an occupation: job or profession. For all cases, each annotation will have the following features: **occupation and subject of occupation (relation).**

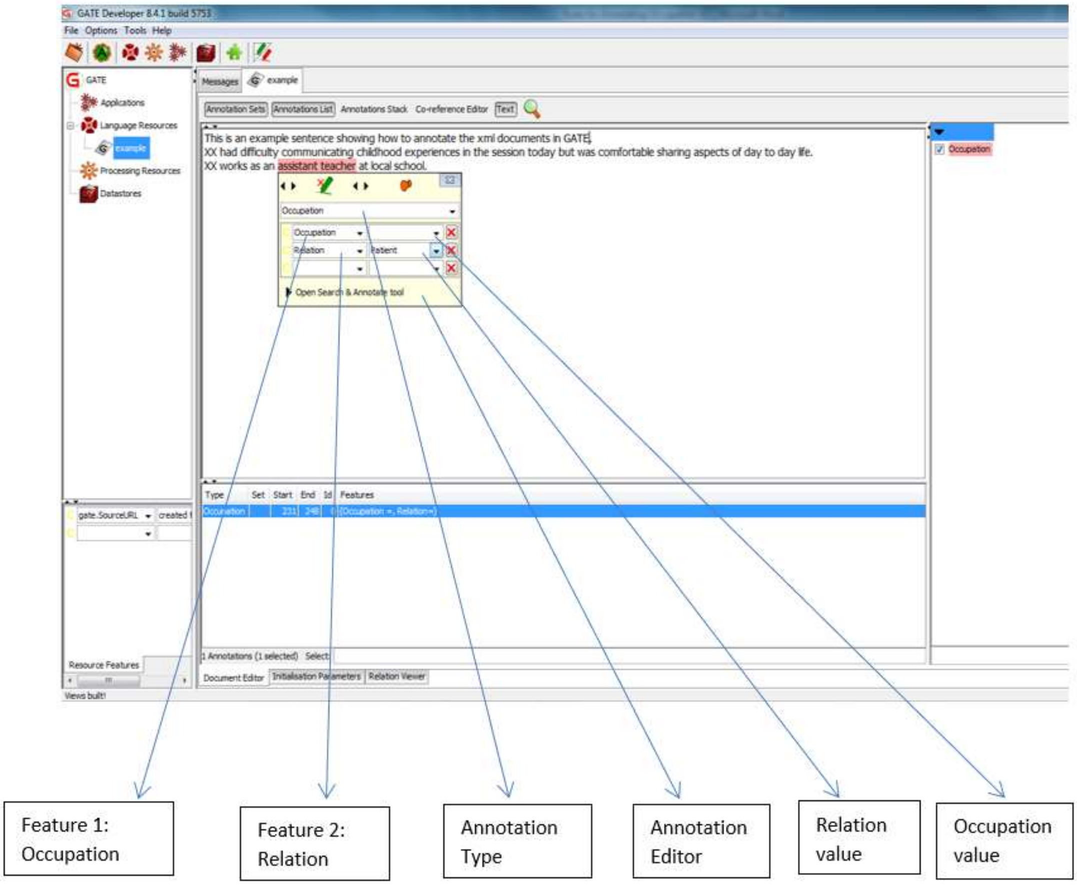

Figure 1: A labelled example illustrating how occupation is annotated in GATE software

Sentence Structure of Annotation

The annotation should be made on adjectives, nouns and verbs in the sentence.

- Title of Occupation

Titles of occupations are always nouns.

Chilman N, *et al. BMJ Open* 2021; 11:e042274. doi: 10.1136/bmjopen-2020-042274

Adjectives should only be annotated when they are part of the occupation type or necessary for describing the occupation e.g. assistant manager, senior consultant. The annotation value is left empty when occupation can be inferred from the exact annotated text.
Example:
XX worked as an assistant teacher – occupation value: empty.
She is a mental health nurse – occupation value: empty
Annotate the adjective and noun.

- **Description of Occupation**
  A) Description of occupation consists of verbs referring to work activities.

Annotate text following:
1) Works for/in/as/at…

Works for real estate - occupation value: estate agent
Works for British Gas - occupation value: British Gas worker
Works for investment bank – occupation value: investment bank worker

2) Job/Role involves, has to do with, includes…

Job involves cleaning houses – occupation value: house cleaner
Role involves writing, teaching – occupation value: writer, teacher

3) Verbs indicating membership

Joined the navy – occupation value: navy officer
Example:
XX worked joined the army after moved to the UK – occupation value: army officer.
Annotate the verb and noun because the noun or verb alone does not describe the occupation sufficiently.

Annotation rules

An occupation or description of work should be annotated regardless of whether it is current or past. However, text indicating whether occupation or description of work is current or past is not required for the annotation unless it offers information on the stability/transience of the occupation.

Examples:
XX is not working at the moment – occupation value: unemployed
XXX has been working as a chef for 3 years- occupation value: chef

XXX worked briefly or worked for a few months or worked every summer – occupation value: other

Do not annotate:

- Punctuation
  - e.g. full stops, semi-colons…
- Adverbs
  - e.g. happily, works hard…
- Articles in front of occupation
  - e.g. the, as, an, a…
- Conjunctions
  - e.g. and, but, if…

*[**UNLESS** these are articles and conjunctions in a double annotation as further below]*

- Adjectives when describing a quality assigned to a job
  - e.g. experienced teacher, qualified electrician
- Verbs that precede title of occupation
  - e.g. became, moved to, promoted to, went to, decided to, etc.
- Text around title of occupation describing place of work **unless** text around title of occupation refers to a field or sector
  - e.g. assistant manager for a phone company – value empty
  - e.g. assistant manager in sales – value fill Sales Assistant Manager
- Time frames or duration of work
  - e.g. worked for 5 years, was a chef in 1995, has worked, is not working

*[**UNLESS** it offers information on the stability/transience of the occupation ie worked briefly or worked for a few months or worked every summer]*

Double annotation:

In the case of two joint occupation descriptions, annotate the same text twice and give a different value each time.

Examples:
Annotate once: he worked in a clothes shop and a kitchen – occupation value: retail worker
Annotate twice: he worked in a clothes shop and a kitchen – occupation value: other - kitchen

Please use this double annotation as sparingly as possible and not when clearly stated occupations or different occupations/work descriptions are joined as below.

Examples:
He worked as a chef and cleaner – two annotations with blank values

He worked on building sites and roofing - two annotations with first value 'labourer' and second 'labourer' or 'other'.

Long job descriptions:
Sometimes clinical record notes are written in a rich and speech-like manner. In cases like this, it is best to annotate a longer piece of text then risk leaving out valuable information.

Examples:
She has worked for only 1 and half year in her life in a wine bar 28yrs ago – occupation value: other- bar

He used to work every summer with his brother at a car wash – occupation value: other- car wash

Occupation

For the occupation value, a title for the work described should be entered. If no title can be created from a work description, 'other' should be entered in the occupation value. In addition, if the title is identical to the work described (job can be inferred from the annotated text), the occupation should be left empty.

**Rules for annotating Employment Status**				
Rule	Rule Description	Example	Occupation Value	Additional Information
Description of job is given, without job title	Annotate with closely related description	Daily role involves operating the machines	Machine operator	
Multiple occupations	All occupations mentioned in the free text (personal history) should be annotated even if they are or appear to be repetitions of an occupation already mentioned within the same history	Chef, 7.5-tonne truck driver<br><br>Worked as kitchen assistant…he helped in a kitchen for 6 months<br><br>She was a teacher…enjoyed her work as a teacher	[blank]<br>[blank]<br><br>Kitchen assistant<br>Other-kitchen<br><br>[blank]<br>[blank]	For chef, truck driver, kitchen assistant and teacher the occupation value should be left empty because the work descriptions are identical to the title that should be given. For 'helped in a kitchen' the occupation value should be 'other'
Related occupations	Annotate all occupations which are mentioned which are	Worked as a social worker and later became a manager	[blank]<br>[blank]	The occupation value should be left empty because the work

	associated with the progress of the same job			descriptions social worker and manager identical to the titles that should be given
Place, sector or employer is mentioned without occupation	Annotate the company or sector	XX works for the council<br>He has been with his present boss for a while	Council worker<br><br>Other	Annotate the company, sector or employer
Loose description of job role which cannot be titled	Annotate the reference to odd jobs which have relevance	XX does various jobs which include, tiling, plumbing…	Tiler, plumber	Annotate the word referring to the odd job

The section below outlines how to annotate the alternative employment statuses: student, retired, self-employed, unemployed, carer, homemaker and other.

**Rules for annotating Student Status**				
Rule	Rule Description	Example	Occupation Value	Additional Information
Student (full time/part time)	Annotate term student or a description of full time/ part time study. Include training/vocational courses.	XX is currently studying XX at university<br><br>He trained as a bricklayer<br><br>He trained in art<br><br>She attended university<br><br>Has a degree in Physics<br><br>He did a Masters in Psychology<br><br>Left University in 1995	Student<br><br>Student<br>[blank]<br><br>Student<br><br>Student<br><br>Student<br><br>Student<br><br>Student<br><br>Student	Two annotations are made to capture student status and occupation value of empty for bricklayer<br><br>'Trained' is annotated by itself whereas 'attended', 'did' 'degree' or 'undertook' need extra information annotated because out of context they wouldn't be sufficient by themselves

Chilman N, *et al. BMJ Open* 2021; 11:e042274. doi: 10.1136/bmjopen-2020-042274

		Graduated with a degree in maths<br><br>He undertook the early career researcher training scheme	Student	
**Rules for annotating Retired Status**				
Retirement	Annotate the term retired or description of retirement	Worked until retirement	Retired	
**Rules for annotating Self-Employed Status**				
Self-employed without job description	Annotate the term or description of self-employed	Patient is self-employed<br><br>He owns a number of properties and shops	[blank]<br><br>Self-employed	The occupation value should be left empty because the job title self-employed is stated<br><br>The occupation value should be self-employed. One annotation.
Self-employed with job description or business/property owner	Annotate the term or description of self-employed and job description	Patient is a self-employed builder<br><br>He owns a number of properties and shops	[blank] [blank]<br><br>Self-employed	These should be two separate annotations (self-employed and builder). Occupation values should be left empty because the job titles self-employed and builder are stated<br><br>One annotation

**Rules for annotating Other Employment Status**	

Difficult to define or job/ job role not stated<br>Simple reference to work	Annotate the verb 'work' or the noun 'job'	Works occasionally on weekends<br>Has had a few other jobs<br>He worked there for 4 years and then left<br>He worked in 1995<br>He worked hard all his life<br>He worked briefly when younger<br>He had a satisfactory job<br>She had numerous jobs<br>He has a creative job<br>She has had three other jobs<br>He did about 8 jobs	Other	Annotate the verb work by itself unless followed by an adverb providing more information about the work itself ie occasionally, the number of jobs ie numerous or the quality ie hard, creative
Sector is not mentioned	Statements not referring to a specific sector or industry should not be annotated	XX moved to the private sector	Other-private	
Army/Navy occupations	Annotate relevant word/ phrase	XX joined the army	Army officer	Always annotate as army or navy officer
Job or occupation relating to shops	Annotate relevant word/ phrase	XX worked part-time in WHSmith	Retail worker	Always annotate as retail worker
Sector or place of work is mentioned but unclear what job the subject undertook	Annotate relevant word/phrase	XX joined his brother in construction<br><br>XX worked in a kitchen	Other-construction	It is not clear what job in construction the patient did so occupation value is given 'other'
**Rules for annotating Unemployed Status**				
Unemployment	Annotate the term unemployed or the description of unemployment	XX has not worked for several years<br>XXX does not work anymore	Unemployed	Unemployment is usually stated in various ways. If the word unemployed is

Chilman N, *et al. BMJ Open* 2021; 11:e042274. doi: 10.1136/bmjopen-2020-042274

		XXX lost his job<br>XXX ran out of work a year ago<br>XXX is currently not working<br>XXX got sacked<br>XXX was made redundant<br>XXX stopped working<br>XXX cannot remember the last job she had<br>Last job was about 5 years ago<br><br>XXX is unemployed	[blank]	annotated, the annotation value should be blank
**Rules for annotating Homemaker Status**				
Housewife<br>househusband	Annotate the term that states that an individual is a homemaker	Mother was a housewife…	[blank]	The occupation value should be left empty because housewife status is stated
**Rules for annotating Carer Status**				
Carer	Annotate the term carer or the description of care role	XX is a carer for elderly mother<br><br>XXX was a carer	Carer<br><br>[blank]	Annotation value of carer should be entered if text annotated includes who the person cared for is. In the second case, where this is not stated, the occupation value is left empty because carer status is stated
**Rules for annotating Volunteer**				
Volunteer	Annotate the noun volunteer or the verb volunteering	XX volunteered with the	Volunteer	

		council once a week		
**Rules for annotating National Service**				
National Service	Annotate the noun national service and the verb preceding	He joined national service<br><br>He did his national service<br><br>He finished national service	Other – national service	
**Rules for annotating illegal activities**				
Prostitution	Annotate relevant word/phrase	XX was working as a prostitute	Sex-worker	Always annotate as sex-worker
Jobs of questionable status/legality	Text referring to income generating jobs that might not be legal	He was a brothel owner<br><br>She made money from dealing drugs	Other- brothel<br><br>Other – drug dealing	Other plus an indication of place or type of work

Subject of Occupation

The relation value should state who the occupation refers to/who carries out the job described. In most cases, the occupation belongs to the patient. The occupation can also belong to the parent/carer of the patient, spouse, relative or other.

**Rules for annotating Subject of Occupation**				
Rule	Rule Description	Example	Relation Value	Additional Information
Patient	The occupation annotated should belong to the patient	Patient was a butcher for XX years	Patient	
Parent/ Carer / Guardian	The occupation annotated should belong to the father or mother of the patient.	Father works as a mechanic	Father	
Spouse	The occupation annotated should belong to the spouse	Husband works for the government doing research	Spouse	The occupation of the spouse should still be recorded even if the text suggests they are no longer together
Relative	The occupation annotated belongs to a	XX's brother discussed the issues	Brother	Relations include: sibling,

Chilman N, *et al. BMJ Open* 2021; 11:e042274. doi: 10.1136/bmjopen-2020-042274

	family member of the patient who is not the parent/carer or spouse	faced being XX's carer and working as a shop assistant…		cousin, aunt, uncle, niece, child, nephew, and grandchild
Girlfriend/Boyfriend/ Partner	The occupation annotated should belong to the patient's girlfriend/boyfriend	XX's girlfriend was a carer for the elderly	Girlfriend	
Other	The occupation annotated does not belong to the patient or patient's relative	The nurse came round to see XX	Other	

Chilman N, *et al. BMJ Open* 2021; 11:e042274. doi: 10.1136/bmjopen-2020-042274

Exclusion Criteria

Rule	Rule Description	Example	Value	Additional information
Future Plans	Future plans to work should not be annotated	XX plans to start role	No annotation	
Hypothetical statements	Text referring to hypothetical scenarios or worries about losing job	XX said he would have left his job if he thought he couldn't cope<br><br>XX was worried he was going to get sacked<br><br>She would have quit if they hadn't given her a raise	No annotation<br><br>No annotation<br><br>No annotation	
When 'work' is used as an adjective<br><br>Describing quality of work without explicitly stating having one		She had great work ethics<br><br>Her work performance deteriorated<br><br>She didn't like her work colleague<br><br>Her job was really good<br><br>He didn't enjoy working there	No annotation	

General Tips: THINK LIKE AN OCCUPATION MACHINE!

1) The machine doesn't have any context

We annotate personal history segments which, if rich, give us a good idea of an individual's story. The machine does not have that reference and if, for example, we annotate 'stopped' in "he worked for 5 years and then stopped" as 'unemployed' we are essentially teaching it to recognise the word 'stopped' as referring to unemployment. Imagine what will happen when we run this application all over CRIS! Ask, if unsure - does the machine understand the annotation I have assigned regardless of context? What will happen if it learns to recognise it as such in another context?

2) The machine loves more of the same

You come across a personal history segment that has 'worked' 3 times, 'labourer' 2 times, 'jobs' 4 times and 2 'sacked'. The machine doesn't know that these have been repeated as it has no context. Also, the more 'labourers' it gets fed, the more it will learn to unequivocally recognise them automatically in any context. Annotate them all!

3) The machine is as smart as you

If you feel you are spending too long making annotation decisions or find a rule that is making your annotations inconsistent, the machine will think the same. Ask questions no matter how silly they seem!

References

1. Perera, G., et al., *Cohort profile of the South London and Maudsley NHS Foundation Trust Biomedical Research Centre (SLaM BRC) case register: current status and recent enhancement of an electronic mental health record-derived data resource.* BMJ open, 2016. **6**(3): p. e008721.

Appendix 1

A timeline of actions leading to guideline and application development

Natasha Chilman, Anna Kolliakou

Date	Action	Outcome
July 2017	Preliminary meeting with research team<br><br>Esther annotated 100 personal history extracts with no guidelines<br><br>Feedback on annotations and guideline ideas discussed by research team	Development of GV1 (Guidelines Version 1)
August 2017	Anna M annotated 50 of the above 100 extracts using GV1, recommended changes	Development of GV2
August 2017	Comments given by research team on GV2	Development of…<br>GV2.1<br>GV2.2<br>GV2.3<br>GV2.4
September 2017	1,262 personal history documents extracted:<br>- Esther annotated 500 using GV2.4<br>- Shirlee double-annotated 200 of these using GV2.	Inter-annotator agreement for 200 double-annotated documents: Cohen's Kappa calculated by GATE = 72% for occupation, 87% for relation<br><br>Development of GV2.5
October-November 2017	40 case examples were written by Anna K and annotated by Anna K, Lisa, Billy, Shirlee and Angus.	Collective agreements made on rules<br><br>Started development of GV2.6
November 2017	Above case examples were given to Karen and Zoe who annotated according to GV2.5	Collective agreements made on rules

		Finished development of GV2.6
November-December 2017	1000 new personal history documents extracted:<br>- Karen annotated all 1000 using GV2.6<br>- Zoe double-annotated 200 of these using GV2.6	Inter-annotator agreement for 200 double-annotated documents:<br>Cohen's Kappa =<br>77% for occupation<br>72% for relation<br><br>In total, 1000 documents = 'gold-standard' annotated corpus
March 2018	GV2.6 was finalised and so was re-named GV3. The 1000 annotated documents were stratified by gender, length of extract, and occupation feature type (labelled as 'other' vs 'non-other' – see guideline for more detail).	334/1000 stratified annotated extracts were sent to Xingyi (University of Sheffield) to develop the application: 257 were used as a training set, 77 were used as a validation set.
April 2018	Application version 1 (AV1) created by Xingyi, sent to Anna K who manually checked application output on the 77 test corpus and precision, recall and F-measures were calculated by GATE evaluation package, feedback provided to Xingyi on application areas for improvement.	Development of AV2
April 2018	As above: AV2 ran on 77 documents, manually checked and measures calculated by GATE evaluation package, feedback provided.	Development of AV3
June 2018	As above: AV3 ran on 77 documents, manually checked and measures calculated by GATE evaluation package, feedback provided.	Development of AV3.1
August 2018	AV3.1 included three different application versions, all ran on 77 documents, manually checked and measures calculated by GATE evaluation package, feedback provided.	Development of AV3.2
November 2018	AV3.2 (one version) was run on 77 documents. The GATE evaluation package was under-estimating the performance of the application, as it classified that if an occupation feature was 'blank' then it was not labelled correctly. Please see guideline for instructions on use of 'blank' feature annotations. These type of annotations came up often in the text.	Development of AV3.3

Chilman N, et al. BMJ Open 2021; 11:e042274. doi: 10.1136/bmjopen-2020-042274

	A new evaluation package ('revised' GATE evaluation) was created which correctly identified 'blank' annotations as a hit. This increased the F-measure and was felt to more accurately reflect the application performance when checking the output manually.<br><br>A small formatting change was made to the guideline, creating GV4, but there was no change in rule content.<br><br>Further feedback sent to Xingyi.	
November-December 2018	AV3.3 was run on the 77 documents, manually checked and F-measures calculated by revised GATE evaluation package, feedback sent to Xingyi.<br><br>A decision was made that the 77 documents needed to be re-annotated which was completed by Anna K in December 2018.	AV3.3 was updated
January-February 2019	Updated AV3.3 was run on both newly annotated 77 documents and previously annotated 77 documents. Barely any difference found in impact on F measure (a very small increase: old annotations F=0.890, new annotations F=0.896).<br><br>Updated AV3.3 run on newly annotated 77 documents, manually checked and F measures calculated by revised GATE evaluation package, feedback sent to Xingyi.	Development of AV3.4
April 2019	As the application was performing reasonably well on the 77 personal history documents, AV3.4 was run on the whole of CRIS. Anna K eyeballed the output and sent feedback to Xingyi for areas for improvement.	AV3.4 was updated to two versions: AV3.4(with machine learning) and AV3.4Revised (without machine learning)
June-July 2019	Both AV3.4 and AV3.4Revised were run on whole CRIS. Anna and Natasha manually checked 200 random personal-history-only documents, and 100 random CRIS documents. Areas for application improvement were sent to Xingyi.	Development of AV4
August 2019	AV4(ML) and AV4(Revised) were run on the whole CRIS. Training corpus of 77 documents was used to evaluate application on GATE. Anna and Natasha manually checked 200 random personal history-only documents, and 100 random CRIS documents (test corpus).	Results from performance of both applications on training corpus and test corpus is available in Supplementary File 3. Application reached good levels of performance

		(precision and recall all >0.79 on a test corpus). The machine learning application performed slightly better so this was chosen over the rule-based approach. However occupation ownership remained an issue, where many of the occupations retrieved belonged to people other than the patient e.g. clinicians. The application did not consistently annotate the relation of the occupation correctly, for example often 'psychiatrist' was annotated as belonging to the patient.
September-November 2019	Following occupation ownership issues identified in the manual evaluation, team meetings were held and it was decided to add an occupation 'filter' to the application. This is a list of occupations which have the most common incorrect relations (e.g. psychiatrist, social worker) – where the application incorrectly annotates the occupation as belonging to the patient. The occupations included in the filter will be assigned a 'other' relation, rather than 'patient' relation. This will mean that we can be more confident that the occupation extracted belongs to the patient. The team reflected that we may miss a small number of true positives this way (e.g. psychiatrists who are patients), but the risk of retrieving incorrect patient occupations is greater, plus healthcare professionals often go to different occupational services for mental health support so are less likely to be included in this sample of electronic health records.<br><br>Method:<br>- Natasha extracted occupations with ≥100 annotations across CRIS. She then sorted these occupations into 3 categories: those which should definitely be added to the filter (e.g. psychiatrist), those which she was not sure about (e.g. interpreter) and those not to add to the filter (e.g. construction).<br>- Out of those which she was not sure about, Natasha checked between 10-40 documents for the number of true positives retrieved by the application	AV4 with machine learning was updated by Xingyi to include the occupation filter, where the occupations on the filter list were assigned the relation 'other' rather than patient.

	(where the occupation was annotated correctly as belonging to the patient). During this process Natasha checked a total of 2,390 documents.<br>- Jay and Anna then went through this list to make collective decisions with Natasha on the unsure occupations. The filter list of occupations was then sent to research team for approval, then sent to Xingyi to add to the app.	
January-February 2020	The application was run over the whole of CRIS with the health/social care occupation filter applied.	Natasha firstly checked accuracy of 400 annotations made by the application: 200 from personal history documents only (precision all annotations = 96.00%, precision patient annotations only = 97%), and 200 annotations over other CRIS document types (precision all annotations = 93.00%; precision patient annotations only = 66%). Of the last estimate, many false positives were for occupation annotations for 'other'.
February 2020	Natasha checked 200 'other' occupation annotations to test the accuracy of this annotation and whether it should be excluded.	Precision for 'other' annotations only reached 23.5%. The false positives for this annotation seemed to fit 3 categories: text about job-seeking (e.g. looking for work), text about working on health/personal goals (e.g. working on his anxiety) or other incorrect annotations (e.g. blood work).
March 2020	Natasha looked at recall and precision more closely. Jyoti ran the application over the personal history table in gate (with extracts accessed via Dave Chandran's personal history app). Natasha selected 200 random documents from this personal history table, annotated them according to this occupation annotation guideline (excluding 'other' annotations), and then checking to see whether the app had identified these occupations (recall) or had identified any false positives (precision). As patient occupations are only mentioned rarely in the clinical record, it was not feasible to do a recall/precision check on all other types CRIS documents, therefore personal history	When looking at all occupation relation annotations, the app had a precision level of 90.04 and recall level of 85.77. When looking at patient relation only annotations, the application reached precision of 77.33 and recall of 79.37.

Chilman N, *et al. BMJ Open* 2021; 11:e042274. doi: 10.1136/bmjopen-2020-042274

	documents are chosen as a targeted and feasible document to check.	

Appendix 2

Annotation Guidelines Version 1

Date: 04/08/2017

This guideline outlines the process for annotating occupation status in GATE. The term highlighted should be the word(s) in the free text which indicates the occupation of an individual, as described in the personal history of the patient. After reading the free text, annotations should be made on the word(s) which are related to an employment status or an occupation: job or profession. For all cases, each annotation will have the following features: **occupation and subject of occupation.** The exclusion criteria outline when no annotations should be made.

**Rules for annotating Occupation Status**			
Rule	Rule Description	Example	Actual Annotation
Multiple occupations	All occupations mentioned in the free text (personal history) should be annotated	Chef, 7.5-tonne truck driver	Occupation: chef, truck driver
Working role is given, without occupation mentioned	Annotate with closely related description	Daily role involves operating the machines	Occupation: production worker/machine operator
Related occupations	Annotate all occupations which are mentioned which are associated with the progress of the same job	Worked as a social worker and later became a manager	Occupation: social worker, social work manager
Place or sector is mentioned without occupation	Annotate the company or sector	XX works for the council	Occupation: council worker
Loose description of job role which cannot be titled	Annotate the reference to odd jobs which have relevance	XX does various jobs which include, tiling, plumbing…	Occupation: Tiler and Plumber

**Rules for annotating Student Status**			
Student (full time/part time)	Annotate term student or a description of full time/ part time study	XX is currently studying XX at university	Occupation: student
**Rules for annotating Retired Status**			
Retirement	Annotate the term retired or description of retirement	Worked until retirement	Occupation: retired
**Rules for annotating Self-Employed Status**			
Self-employed without job description	Annotate the term or description of self-employed	Patient is self-employed	Occupation: self-employed
Self-employed with job description	Annotate the term or description of self-employed and job description	Patient is a self-employed builder	Occupation: self-employed, builder
**Rules for annotating Other Occupation Status**			
Difficult to define or job/role not stated	Annotate relevant phrase	Works occasionally on weekends	Occupation: other
**Rules for annotating Unemployed Status**			
Unemployment	Annotate the term unemployed or the description of unemployment	XX has not worked for several years	Occupation: unemployed

Chilman N, *et al. BMJ Open* 2021; 11:e042274. doi: 10.1136/bmjopen-2020-042274
