# Supplementary material for: Text mining occupations from the mental health electronic health record: a natural language processing approach using records from the Clinical Record Interactive Search (CRIS) platform in south London, UK
Source: BMJ Open. 2021 Mar 25;11(3):e042274. doi: 10.1136/bmjopen-2020-042274 (PMC7996661; doi:10.1136/bmjopen-2020-042274)
Supplement: Supplementary data [file bmjopen-2020-042274supp003.pdf]

Supplementary File 3: Machine learning and rule-based approaches to text-mine occupations from the electronic health record

The Occupation Application Pipeline

The occupation extraction application works by implementing 5 steps: 1) Text pre-processing, 2) Occupation mention detection, 3) Occupation title assignment, 4) Occupation relation extraction and 5) Occupation filtering. The pipeline of the application is demonstrated in Figure 1.

For a free-text input, we pre-process the input document through: (1) an English Tokeniser, (2) GATE's Morphological Analyser (lemmatise and tokens), (3) a sentence splitter (as the occupation extraction is conducted at sentence level), (4) a POS tagger (where we obtain part-of-speech for each token, and the part-of-speeches are used as features in later rule and machine-learning modules), and (5) ANNIE Name Entity Transducer (the default Name Entity Transducer embedded in the GATE system; these entities are used as features in later rule and machine learning modules).

After text pre-processing, we detect occupation mentions in the free-text by using: (1) a Conditional Random Field algorithm-based machine learning approach, and (2) a JAPE rule based approach. We combine the results from both approaches to increase the recall level. A rule-based title assignment module is applied to assign the occupation titles (e.g. builder, doctor, etc) for extracted occupation mentions.

When identifying who the occupation belongs to ('relation' extraction), we first extract the relation phrases (e.g. patient, mother, etc) from the surrounding context of the occupation mention. We then use a rule-based and machine-learning (support vector machine)-based classifier to classify the occupation relation. In this application we prefer rule-based relation classifier output to the machine-learning output when available – the machine-learning relationship is only used when there is no output from the rules.

The final step of the pipeline is occupation filtering, which is a rule-based approach to filter out common false positives and health/social care occupations.

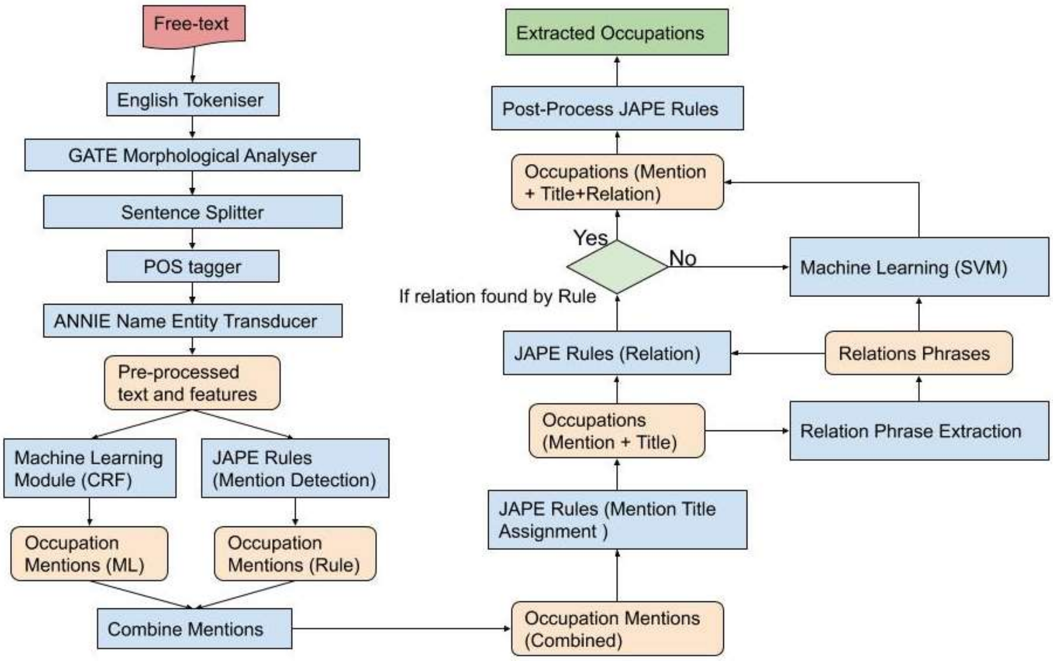

Figure 1: The pipeline of the occupation application. [1]

[1] The red box represents the input text, blue boxes represent NLP modules, light orange boxes represent the intermediate output from the NLP modules and the green box represents the extracted occupation.

Comparing combined machine-learning and rule-based approaches with rule-based only approaches

During testing we evaluated two versions of the application: one with machine-learning and rule-based combined approaches, and one with rule-based approaches only (without machine-learning). In the application version with rule-based approaches only, all machine-learning components in the occupation application pipeline (Figure 1) were removed.

The two versions of the applications were run over free-text documents in the case register of electronic health records. Where an occupation was identified by at least one of the application versions, we extracted 100 documents which included sections of text entitled 'personal history' and 100 documents which did not include a 'personal history' section (e.g. other 'Events' or 'Attachments'). One document may have multiple occupation annotations – all of which were evaluated. Where an occupation was annotated correctly this was counted as a true positive for occupation precision; where who the occupation belonged to was annotated correctly this was counted as a true positive for occupation relation; and where both were correct this was counted as an overall true positive for precision (table 1).

Both applications performed similarly, however the application with machine learning performed best on both personal history and other document types when assigning the occupation 'relation' (relation precision=0.91 on personal history documents). As the authors wanted to maximise precision regarding who the occupation belonged to (particularly for the patient), this application version was chosen for further developments.

Documents	Application version	Precision	Occupation precision	Relation precision
100 personal history	With Machine-Learning	0.92	0.96	0.91
	Without Machine-Learning	0.95	0.96	0.85
100 other CRIS document types	With Machine-Learning	0.79	1	0.68
	Without Machine-Learning	0.94	1	0.58

Table 1: Evaluation of occupation applications on the test corpus of documents where the applications had identified an occupation, calculated manually.
**Precision = true positive annotations/all annotations*
*** Occupation precision = true positive occupation titles/all occupation titles*
****Relation precision = true positive relation assignments/all relation assignments*
