# Supplementary material for: Text mining occupations from the mental health electronic health record: a natural language processing approach using records from the Clinical Record Interactive Search (CRIS) platform in south London, UK
Source: BMJ Open. 2021 Mar 25;11(3):e042274. doi: 10.1136/bmjopen-2020-042274 (PMC7996661; doi:10.1136/bmjopen-2020-042274)
Supplement: Author's manuscript [file bmjopen-2020-042274.draft_revisions.pdf]

BMJ Open

BMJ Open is committed to open peer review. As part of this commitment we make the peer review history of every article we publish publicly available.

When an article is published we post the peer reviewers' comments and the authors' responses online. We also post the versions of the paper that were used during peer review. These are the versions that the peer review comments apply to.

The versions of the paper that follow are the versions that were submitted during the peer review process. They are not the versions of record or the final published versions. They should not be cited or distributed as the published version of this manuscript.

BMJ Open is an open access journal and the full, final, typeset and author-corrected version of record of the manuscript is available on our site with no access controls, subscription charges or pay-per-view fees (http://bmjopen.bmj.com).

If you have any questions on BMJ Open's open peer review process please email info.bmjopen@bmj.com

BMJ Open

Text-mining occupations from the mental health electronic health record: a natural language processing approach using records from the Clinical Record Interactive Search (CRIS) platform in South London, UK.

Journal:	*BMJ Open*
Manuscript ID	bmjopen-2020-042274
Article Type:	Original research
Date Submitted by the Author:	01-Jul-2020

Complete List of Authors:	Chilman, Natasha; King's College London, Department of Psychological Medicine; Insitute of Psychiatry, Psychology & Neuroscience<br>Song, Xingyi; The University of Sheffield<br>Roberts, Angus; King's College London, Institute of Psychiatry, Psychology and Neuroscience<br>Tolani, Esther; King's College London, Institute of Psychiatry, Psychology & Neuroscience<br>Stewart, Robert; King's College London, Institute of Psychiatry; South London and Maudsley NHS Foundation Trust<br>Chui, Zoe; King's College London, Institute of Psychiatry, Psychology & Neuroscience<br>Birnie, Karen; King's College London; King's College Hospital NHS Foundation Trust<br>Harber-Aschan, Lisa; King's College London<br>Gazard, Billy; King's College London<br>Chandran, David; South London and Maudsley NHS Foundation Trust, NIHR Biomedical Research Centre<br>Jyoti, Jyoti; South London and Maudsley NHS Foundation Trust, NIHR Biomedical Research Centre<br>HATCH, STEPHANI; King's College London, Institute of Psychiatry, Psychology and Neuroscience<br>Kolliakou, Anna; King's College London, Institute of Psychiatry, Psychology & Neuroscience<br>Das-Munshi, Jayati; King's College London, Department of Psychological Medicine, Institute of Psychiatry, Psychology & Neuroscience

Keywords:	MENTAL HEALTH, Health informatics < BIOTECHNOLOGY & BIOINFORMATICS, EPIDEMIOLOGY, Adult psychiatry < PSYCHIATRY

SCHOLARONE™ Manuscripts

I, the Submitting Author has the right to grant and does grant on behalf of all authors of the Work (as defined in the below author licence), an exclusive licence and/or a non-exclusive licence for contributions from authors who are: i) UK Crown employees; ii) where BMJ has agreed a CC-BY licence shall apply, and/or iii) in accordance with the terms applicable for US Federal Government officers or employees acting as part of their official duties; on a worldwide, perpetual, irrevocable, royalty-free basis to BMJ Publishing Group Ltd ("BMJ") its licensees and where the relevant Journal is co-owned by BMJ to the co-owners of the Journal, to publish the Work in this journal and any other BMJ products and to exploit all rights, as set out in our licence.

The Submitting Author accepts and understands that any supply made under these terms is made by BMJ to the Submitting Author unless you are acting as an employee on behalf of your employer or a postgraduate student of an affiliated institution which is paying any applicable article publishing charge ("APC") for Open Access articles. Where the Submitting Author wishes to make the Work available on an Open Access basis (and intends to pay the relevant APC), the terms of reuse of such Open Access shall be governed by a Creative Commons licence – details of these licences and which Creative Commons licence will apply to this Work are set out in our licence referred to above.

Other than as permitted in any relevant BMJ Author's Self Archiving Policies, I confirm this Work has not been accepted for publication elsewhere, is not being considered for publication elsewhere and does not duplicate material already published. I confirm all authors consent to publication of this Work and authorise the granting of this licence.

For peer review only

Text-mining occupations from the mental health electronic health record: a natural language processing approach using records from the Clinical Record Interactive Search (CRIS) platform in South London, UK.

Natasha Chilman[1*], Xingyi Song[3], Angus Roberts[1], Esther Tolani[1], Robert Stewart[1,2] Zoe Chui[1], Karen Birnie[1, 5], Lisa Harber-Aschan[1], Billy Gazard[1], David Chandran[2], Jyoti Sanyal[2], Stephani L Hatch[1,4], Anna Kolliakou[1, **], Jayati Das-Munshi[1,2, 4**]

***Joint senior author*

***Corresponding author contact information:**

Natasha Chilman, East Wing 3.16, Department of Psychological Medicine, Institute of Psychiatry, Psychology & Neuroscience, Kings College London, DeCrespigny Park, London, SE5 8AF. Telephone: +44 796968 8554. Email: natasha.chilman@kcl.ac.uk

Author details:

[1] Institute of Psychiatry, Psychology and Neuroscience, King's College London, London, UK. [2] South London and Maudsley NHS Foundation Trust, London, UK. [3] University of Sheffield, Sheffield, UK. [4]Economic and Social Research Council (ESRC) Centre for Society and Mental Health, King's College London, UK. [5]King's College Hospital NHS Trust, London, UK.

ORCID identifiers:

Natasha Chilman 0000-0002-9661-5098

Xingyi Song 0000-0002-4188-6974

Angus Roberts 0000-0002-4570-9801

Esther Tolani 0000-0002-7415-0859

Robert Stewart 0000-0002-4435-6397

Zoe Chui 0000-0001-6844-6779

Karen Birnie 0000-0003-4123-1676

Lisa Harber-Aschan 0000-0002-6464-485

Billy Gazard 0000-0002-7562-539

For peer review only - http://bmjopen.bmj.com/site/about/guidelines.xhtml

David Chandran 0000-0002-0123-666X

Jyoti Sanyal – N/A

Stephani Hatch 0000-0001-9103-2427

Anna Kolliakou 0000-0003-1234-4129

Jayati Das-Munshi 0000-0002-3913-6859

Word Count

3,999

Keywords

Mental Health

Health informatics

Epidemiology

Adult psychiatry

For peer review only

ABSTRACT

Objectives

We set out to develop, evaluate, and implement a novel application using natural language processing to text-mine occupations from the free-text of psychiatric clinical notes.

Design

Development and validation of a natural language processing application using General Architecture for Text Engineering (GATE) software to extract occupations from de-identified clinical records.

Setting & Participants

Electronic health records from a large secondary mental health provider in south London, accessed through the Clinical Record Interactive Search (CRIS) platform. The text-mining application was run over the free-text fields of the electronic health records of 341,720 patients (all aged ≥16).

Outcomes

Precision and recall estimates of the application performance; occupation retrieval using the application compared to structured fields; most common patient occupations; and analysis of key sociodemographic and clinical indicators for occupation recording.

Results

Using the structured fields alone, only 14% of patients had occupation recorded. By implementing the text-mining application in addition to the structured fields, occupations were identified in 57% of patients. The application performed on gold-standard human-annotated clinical text at a precision level of 0.79 and recall level of 0.77. The most common patient occupations recorded were ‘student’, and ‘unemployed’. Patients with more service contact were more likely to have an occupation recorded, as were patients of a male gender, older age, and those living in areas of lower deprivation.

Conclusion

This is the first time a natural language processing application has been used to successfully derive patient-level occupation from the free-text of electronic mental health records, performing with good levels of precision and recall, and applied at scale. This may be used to inform clinical studies relating to the broader social determinants of health using electronic health records.

For peer review only - http://bmjopen.bmj.com/site/about/guidelines.xhtml

ARTICLE SUMMARY

Strengths and Limitations

- The application was developed on a sizeable corpus of training and test data from a large routine dataset, which was applied at scale over the record, providing us with insights into the occupations of patients using secondary mental health services.
- The application was rigorously evaluated using gold-standard and cross-checking strategies.
- The application was developed and tested in a single site electronic health record system in the UK – the application will require validation on other similar systems before use with them.
- The application does not identify the temporality of occupations; it is unclear whether the extracted occupations are currently or previously held by the patient.
- Health and social care occupations were prevented from being assigned to the patient as these could not be ascertained with confidence, therefore the application cannot yet identify where a patient holds a health/social care occupation.

For peer review only - http://bmjopen.bmj.com/site/about/guidelines.xhtml

INTRODUCTION

Occupation and mental illness are highly interrelated. There are long-standing concerns that unemployment rates are considerably higher for people with mental illness [1, 2], and work participation has been described as among the most important factors for recovery by clinicians and service users alike [3, 4]. People with mental illnesses may also undertake precarious poorly paid work which could have further negative impacts on mental health [5]. Moreover, occupation is a fundamental individual-level indicator of socio-economic position as it is predictive of financial income and material resources and is indicative of wider class interactions [10]. Recent systematic reviews have called for large and detailed longitudinal studies to investigate predictors of occupational functioning and to examine how and when occupation is associated with clinical outcomes in mental health cohorts, as this is currently poorly understood [6, 7].

Research using electronic health records (EHRs) allows for large-scale collection of sociodemographic and clinical information that would otherwise be logistically challenging to collect using traditional epidemiological approaches [8]. However, EHR research has major limitations including that information relating to occupation is either not recorded routinely or is poorly captured within standard EHR systems [9]. As there are no existing methods, to our knowledge, to reliably extract occupations from the psychiatric EHR, this is a problematic barrier for desirable research where occupation is an indicator of socioeconomic status and in research examining the relationships between occupation, mental illness and recovery.

Patient information can be recorded in the structured fields of the EHR, where the clinician records categorical or numerical data. In many psychiatric EHR systems, patient information is recorded in narrative text sections of the record, known as the 'free-text' fields, for example in notes describing patient contact [11]. Information recorded in this way is harder to extract. Clinicians may only record the patient's occupation in such free-text fields and not the structured fields, making it more complicated, time consuming and labour intensive to identify the patient's occupation [9]. Natural language processing (NLP) methods have the potential to overcome this obstacle by developing and applying algorithms to extract the relevant textual information. NLP methods have previously been used successfully for text-mining from mental health EHRs, for example to identify smoking status and symptoms of severe mental illness [12-16]. This paper traces the development of a novel application using NLP methods to extract patient occupations from the free-text of EHRs from a large mental health Trust in south London, UK. We then provide profile information on the most frequently extracted occupations for patients using secondary mental health services, and clinical and sociodemographic factors associated with recorded occupation data compared to missing occupation data.

For peer review only - http://bmjopen.bmj.com/site/about/guidelines.xhtml

MATERIALS AND METHODS

Setting

Data for the development of the application were obtained from the South London and Maudsley (SLaM) Biomedical Research Centre (BRC) Case Register: a repository of de-identified clinical data from the EHRs of individuals receiving care from SLaM secondary mental health services. SLaM covers a socially and ethnically diverse inner-city area of approximately 1.3 million people [17]. The register contains over 350,000 de-identified patient records which are available for research purposes through the Clinical Record Interactive Search (CRIS) platform. CRIS was developed at SLaM in 2008 and similar resources have subsequently been implemented at several other mental health Trusts in the UK. The present application was developed over the years 2017-2019 and was implemented in January 2020.

Datasets

Figure 1 describes how the CRIS-derived dataset was used for cycles of application development and evaluation, and summarises the key steps taken. Age restrictions were implemented throughout document selection: free-text documents were only extracted where the patient was aged 16 and above at time of document extraction. There were no date restrictions. Free-text documents were retrieved from several different sections in this EHR, for example documents specifically for clinical risks assessments and separate sections for discharge summaries. Further detail on the types of documents used at each stage of application development can be found in supplementary file 1.

Developing, Evaluating and Implementing the Application

Manually annotating occupation in the free-text (Figure 1, steps 1-3)

Personal history sections of psychiatric assessments typically describe the patient's occupation as well as education and family history. Personal history sections of documents were therefore extracted from the free-text fields of records at a document level using an NLP application (which was shown to have a precision rate of 78%) developed by DC (N=67,383). Typically these documents were derived from documents of the 'attachments' type, which is a word-processed document such as a letter to or from the patient's primary care physician; and 'events', which are short pieces of text used to record some detail of a clinical encounter.

Occupations were identified in personal history documents by an interdisciplinary team of trained researchers, including clinicians, bioinformaticians and mental health researchers. In common with the NLP community, we refer to this task of marking mentions of occupation text as annotation. A set of occupation annotation guidelines were developed through an iterative process of manual annotation practice, team discussions and agreed annotation rulemaking (supplementary file 2). These guidelines

For peer review only - http://bmjopen.bmj.com/site/about/guidelines.xhtml

specified when and how an occupation should be identified, annotated and extracted from the text. An occupation annotation was defined as having two parts. Firstly, the *occupation* itself was annotated. This could be an occupation title, for example a 'builder'; or an occupation description, for example 'construction'. Secondly, the occupation *relation* was specified: who the occupation belongs to, for example the patient or their family member. In total, 600 personal history documents were manually annotated to develop the annotation guidelines (ET, AK, SM, KB, ZC, AR). Once the guidelines were developed, a further set of 1000 personal history documents were manually annotated on the General Architecture for Text Engineering (GATE) platform [18] using the guidelines, where 200 were double annotated to evaluate inter-annotator reliability.

Application development (Figure 1, step 4)

Out of the 1000 gold-standard annotated personal history documents, 77 documents with a total of 405 occupation annotations were used as a training set for the application. The application was developed by XS on the GATE platform [18]. To check the performance of the application throughout development, precision and recall metrics were estimated using a customized performance tool developed by XS on GATE. Precision was the proportion of occupations correctly annotated, to all occupations annotated (whether correct or incorrect). Recall was the proportion of occupations correctly annotated, to all occupations that could have been correctly annotated. The application outputs were manually checked by the Clinical Informatics Interface and Network Lead at the NIHR Biomedical Research Centre (AK). Any problems identified were addressed in each version of the application. An iterative process of application development, evaluation of performance using GATE and manual checks was repeated 10 times until the application reached a good level of performance on the training set.

Machine-learning approach testing (Figure 1, steps 5-6)

Two early versions of the application were developed for testing over unannotated documents in the CRIS case register: one version used combined machine-learning and rule-based approaches, and the second version used rule-based approaches only. This was due to a concern that the application had therein been developed on limited training data, and the trained model may not generalise well on the free-text other than personal history documents, which could lead to a loss in precision when implemented over the EHR. Specifically, the machine-learning approaches involved a trained conditional random field classifier. Two researchers (NC, AK) manually calculated precision performance for both versions of the application on 100 personal history documents (in domain testing data) and 100 other free-text document types (out domain test data) which had at least one occupation extraction and were previously unseen by the application in development. Whilst both application versions performed well when text-mining occupations (precision $\geq$0.79, supplementary file 3), the application with machine-learning approaches performed at the highest level of precision when

For peer review only - http://bmjopen.bmj.com/site/about/guidelines.xhtml

assigning the occupation relation - i.e. who the occupation was held by. The research team concluded from this testing phase that the NLP application with combined machine-learning and rule-based approaches was most appropriate for the task of text-mining patient occupations.

The healthcare occupation filter (Figure 1, step 7)

The evaluation of the application performance over CRIS documents revealed that the most common false positives included extractions where the healthcare professional involved in the patient's care was incorrectly annotated as the patient's occupation (96% of annotations manually checked were health/social care occupations). To deal with this issue, health and social care occupations were added to a filter. The application then implemented a rules-based step where the app was programmed so that filtered healthcare occupations were not annotated as belonging to the patient. Occupations added to this filter included variations on terms for psychiatrists and doctors, therapists, nurses, and social workers, following the checking of 2,390 documents to confirm that these were common false positives.

Application implementation and testing (Figure 1, steps 8-10)

The final version of the text-mining application with the healthcare filter applied was run over 10 free-text fields, including those where personal history sections were found, in the records of all patients on the CRIS case register aged 16 and above. The fields included sections of the record such as discharge summaries, attachments, events and risk assessments (more detail in supplementary file 1). The application was evaluated on 2 testing sets: 666 gold-standard annotated personal history documents (test corpus 1), and 200 previously unannotated random personal history documents from the CRIS dataset at the time of the application run (test corpus 2). Test corpus 1 was evaluated on GATE, and test corpus 2 was manually checked for occupations and then cross-referenced with the application output. The performance metrics considered the precision and recall level for the annotations made by the application, where both the occupation annotation and the relation classification needed to be accurate to be considered a 'true positive'. It was not feasible in this study to randomly select non-personal-history documents for evaluation as patient occupations were rarely mentioned in the record compared to other information (e.g. medication). As the application extracted an annotation entitled 'other', 200 of these annotations were manually checked for precision to further investigate these instances where the application was unable to assign an occupation title.

The EHR in the present study contains a structured field that is used to record occupation, called the 'Employment_ID'. This was explored on the CRIS platform using SQL queries. The proportion of completed Employment-IDs from the records of all patients over the age of 16 in January 2020 was extracted. The NLP application was simultaneously run over clinical records through CRIS, and the extracted patient occupations were converted into an SQL table. Sociodemographic, clinical and service contact data was also extracted from the structured fields of records using SQL and data was then exported to and analysed in STATA-15 to examine predictors of occupational data extraction using

For peer review only - http://bmjopen.bmj.com/site/about/guidelines.xhtml

logistic regression models. This included the patients age at time of occupation extraction, gender, marital status, ethnicity, index of multiple deprivation (IMD) score and primary diagnosis. Indicators of service contact included number of events in the record, number of face-to-face events in the record, number of spaces in the free-text fields of the record (as a proxy for word count), number of active days under SLaM services, and number of inpatient bed days. These variables were transformed into categories, for example IMD scores were categorised into quartiles of local neighbourhood deprivation. Where data was missing for the extracted variables, this was coded as a 'Not Known' category for each variable.

Logistic regression models examined crude associations between the sociodemographic, clinical, and service contact variables (predictors) and the recording of at least one patient occupation (outcome) from either the structured or free-text fields. The null hypothesis was that none of the predictors would be associated with likelihood of occupation recording. Models were firstly adjusted for amount of contact the patient had with services. Fully adjusted models then accounted for all other sociodemographic and clinical variables. Across all models, likelihood ratio tests were conducted to test the overall association between the variable and occupation recording. The aim of this analysis was to ascertain the characteristics of patients who had occupation recorded in their health record.

For peer review only

RESULTS

Annotating Occupation

When double-annotating 200 personal history documents, two annotators reached a Cohen's kappa agreement of 0.77 for occupation title annotations and 0.72 for occupation relation annotations. Disagreements between annotators included instances where sentences posed unclear or vague references to occupation: for example, in the sentence, "she did several things, such as cleaning, cooking", it was not clear whether these were domestic tasks or occupation-descriptions, demonstrating the complexity of annotating occupation from text. Nonetheless, the Cohen's kappa agreement suggested that occupation could be annotated reasonably consistently across annotators using the annotation guidelines.

Application Development

The application reached a precision level of 0.88 and a recall level of 0.90 on the 'training set' of documents (N=77). The developed application process with combined rule-based and machine learning approaches is described in Figure 2.

Application Performance

When applied to the gold-standard annotated personal history documents (test corpus 1) on GATE, the application performed at a precision level of 0.79 and a recall level of 0.77. Out of the 200 personal history documents which were manually checked for occupations and then cross-referenced with the application output (test corpus 2), when focusing on patient occupations only, the application reached a precision level of 0.77 and recall level of 0.79. An extraction of 'other' as an occupational category was excluded from subsequent analysis, as the check of 200 annotations showed that this annotation only reached a precision level of 0.23 and often referenced job-seeking or non-work behaviours, for example 'working on his anxiety'.

Application Implementation

Figure 2 shows the study population selection process for the implementation of the application over the CRIS case register, leading to an overall sample size of 341,720 patients.

Descriptives

Demographics of the study population at time of occupation extraction is described in Table 1, as well as patient diagnostic categories and two indicators of the amount of service contact the patient has had: the number of 'events' entries added to the EHR, and number of inpatient bed days. The three other extracted indicators for service contact (number of 'face-to-face events', total active days under SLaM mental health services, and number of spaces in the text in the record) were excluded from analysis due to collinearity with the 'events' variable.

For peer review only - http://bmjopen.bmj.com/site/about/guidelines.xhtml

Occupation Extractions

The structured field for employment was populated for 46,705 (13.7%) patients. Prior to the implementation of the healthcare filter, 81.5% patients had at least one patient-occupation extraction. When using the final version of application to extract occupations from the free-text fields with the healthcare filter applied, this recalled at least one patient-related occupation for 184,521 patients (54.0%). By combining structured field and extracted occupations, patient-related occupations were retrieved for 193,616 patients (56.7%) over the dataset.

The structured field for occupation included 13 categories for occupational status, for example 'unemployed' or 'paid employment'. In contrast, the text-mining application retrieved 72,955 different patient-related occupation types. In total there were 3,957,959 patient-related occupation extractions. Multiple occupation types were often extracted per patient (median=4, inter-quartile-range=6).

The top 5 extracted occupations across the total sample of 341,720 patients were: student (98,719 patients had this extraction at some point in the record, 28.9%), unemployed (97,809 patients, 28.6%), carer (61,893 patients, 18.1%), self-employed (36,506, 10.8%) and retired (33,518 patients, 9.8%). The less frequent extractions tended to be more specific occupation types, for example, 'retail worker', and 'banker'. The application also extracted 'undocumented' ways of making money, including 'drug dealer' and 'sex worker'.

Associations with Occupation Recording

Patients were split into two binary categories: those who had an occupation recorded either in the structured field or free-text (n=193,616, 56.7%), and patients who did not have occupation recorded, i.e. missing occupational data (n=148,104, 43.4%). Logistic regressions were used to examine sociodemographic, clinical, and service contact associations with recorded occupations (Table 2).

Across all models, all predictors were strongly associated with a recording of occupation even after fully adjusting for all other variables (likelihood ratio tests p<.0001). When key sociodemographic data was missing from the record, the odds of occupational data being recorded decreased: for example, where the marital status of the patient was 'Not Known', the fully-adjusted odds ratio for a recording of an occupation was 0.49 (95% CI 0.47-0.50) compared to patients who were recorded as married/in a civil partnership/cohabiting. Female patients were significantly less likely to have an occupation extracted compared to male patients, and older patients were most likely to have occupational data recorded compared to the youngest patients. Compared to patients of White British ethnicity, patients of Irish, Black Caribbean, or Black African ethnicity were more likely to have an occupation recorded; whilst Indian, Pakistani, Chinese, Mixed Race or recorded as being from 'other' Asian or ethnic groups were less likely to have occupation recorded. The odds of having occupation recorded were significantly lower for patients who were living in the most deprived local areas compared to the most affluent areas. Generally, patients with

For peer review only - http://bmjopen.bmj.com/site/about/guidelines.xhtml

a primary diagnosis of an affective disorder had a higher odds of an occupation extraction than patients with other diagnoses, including organic disorders. In the crude logistic regression models, patients diagnosed with schizophrenia, schizotypal or delusional disorders were more likely to have occupation extracted (OR 1.61, P5% CI 1.54-1.68). However, once adjusting for amount of contact with services, these patients were significantly less likely to have occupation extracted compared to patients with affective disorders (adjusted OR 0.87, 95% CI 0.83-0.91).

For peer review only

For peer review only - http://bmjopen.bmj.com/site/about/guidelines.xhtml

DISCUSSION

Annotating and extracting occupation from the free-text in clinical records is a challenging task. We have developed a cutting-edge methodology to extract patient occupations with a good degree of confidence from a mental health EHR, and applied this at scale over a large EHR in south London. An important finding was that we could retrieve over double the number of patient occupations using our methodology than when using pre-existing structured fields alone. We could also access a much wider diversity of occupation types: this further detail on occupations held by patients opens up the possibility for the translation of occupations onto a social class schema which would not have been possible with the limited structured field categories. The most prevalent patient occupations were 'student' and 'unemployed'. There were differences between patients where an occupation was recorded and patients where occupation data remained missing: patients with occupations recorded were more likely to be of an older age, male, divorced/separated, living in areas of lower deprivation, and had more contact with mental health services. Across ethnic minority groups, there were mixed findings relating to the recording of occupation. Compared to White British patients, Irish, Black Caribbean and Black African patients were slightly more likely to have a recording of occupation, whereas all other ethnic minority groups were less likely to have a recording. Although it is possible that some of the demographic associations with the recording of occupation in the case notes were impacted upon by residual confounding in adjusted models, these findings may also indicate disparities relating to how occupations are assessed and recorded in the clinical record and should be explored in future work, particularly given the strong correlation of employment with recovery, within the context of mental disorders.

This study broadly supports the work of other studies which indicate that clinicians mostly describe occupation in the free-text of EHR systems, when these are available, rather than structured fields [9]. This study is the first of its kind to text-mine patient occupations from a mental healthcare EHR. There have been several previous efforts to extract patient occupations from other healthcare free-text notes. Occupations have been text-mined from general medical clinical text; however, in these studies the algorithms reached low levels of performance, largely due to a lack of training data [20, 21]. Dehghan and colleagues' text-mined occupation from the clinical records of cancer patients in the UK, reaching similar precision and recall levels to the present study [22]. However, none of these applications distinguished between text-mining occupations belonging to the patient and other relations, had the scope of applying and testing the text-mining methodology at scale across the EHR, or examined associations with extracted versus missing occupational data. The present application therefore represents significant progress in our ability to text-mine patient occupations from the EHR and furthers our understanding of what this may mean in practice.

We found that text-mining greatly increased our retrieval of patient occupations in this psychiatry EHR database. Psychiatric notes may be more detailed than other types of healthcare text (for example, in

For peer review only - http://bmjopen.bmj.com/site/about/guidelines.xhtml

general medicine) when describing the patient's occupation, as this often forms part of psychiatric history taking and assessment. We found that a sizeable proportion of patients over CRIS have at some point been a student or unemployed. A separate NLP application being developed using CRIS data (by author JS) will be able to interrogate this student group further by extracting the patient's level of educational attainment, which will complement the present application. There is also scope to explore older groups of patients who are students but are also working using this methodology. Our finding that unemployment was a dominant occupational category is consistent with the finding that unemployment levels are elevated particularly for those with severe mental illnesses compared to the general population [1, 2]. It may also be the case that patients in this group are formally unemployed but are working in more informal, undocumented ways to make money. This application identified some informal occupations, which is an interesting avenue for further research.

One limitation of our approach is that we could not distinguish the temporality of occupations – whether they were currently or previously held by the patient. Multiple occupations were often extracted for a single patient, adding to the complexity. Whilst there is work ongoing to use NLP to detect temporality in psychiatric healthcare text [19], this remains a challenge. As this application was developed at a single site in the UK, the generalisability of the application may be reduced, firstly to English language and secondly to this catchment area. As it was not possible to assign health and social care occupations to patients with reasonable confidence, we will also be missing patients who hold these occupations; however, we are planning further work to develop this aspect of the application further. Notwithstanding these limitations, this application was developed through an extensive process of training and testing using a large corpus leading to the application of text-mining algorithms for occupation at scale. This methodology is already revealing the kinds of occupations held by patients using secondary mental health services.

The development of this methodology has numerous implications. Firstly, this application will be valuable in allowing researchers to examine relationships between occupation and health in large psychiatric case registers. For example, work is currently underway using this application to investigate predictors of unemployment in a cohort of patients with severe mental illness [23]. As CRIS-like systems are in use over several sites in the UK, there is the scope to test and implement this application in other mental healthcare providers using similar EHR platforms. This application could also have potential practical implications including identifying unemployed patients to target interventions such as Individual Placement and Support (IPS) and retrieving occupational distributions for audits and organisational monitoring in NHS mental health Trusts.

There is room for further progress in this application as the NLP field further develops, including identifying the temporality of occupations and improving relation classification for health and social care occupations. We plan to develop methodology to ascertain the occupational social class of patients,

For peer review only - http://bmjopen.bmj.com/site/about/guidelines.xhtml

using the large diversity of occupations extracted, to further inform health inequalities research specific to mental health. Future studies implementing this application in other CRIS systems may be able to investigate the transferability of the application to other NHS sites in the UK that serve different patient populations. Overall, we hope that this approach will prove useful in forwarding our understanding of the interactions between occupation and health in those with mental illness.

For peer review only

For peer review only - http://bmjopen.bmj.com/site/about/guidelines.xhtml

ORIGINAL PROTOCOL

N/A

FUNDING STATEMENT

This work was supported by the Clinical Record Interactive Search system which is funded by the National Institute for Health Research (NIHR) Biomedical Research Centre at South London and Maudsley National Health Service (NHS) Foundation Trust and King's College London and a joint infrastructure grant from Guy's and St Thomas' Charity and the Maudsley Charity. Specific application development was supported by the Health Foundation, in a grant held by JD.

JD is funded by the Health Foundation working together with the Academy of Medical Sciences, for a Clinician Scientist Fellowship and by the ESRC in relation to the SEP-MD study (ES/S002715/1) and part supported by the ESRC Centre for Society and Mental Health at King's College London (ESRC Reference: ES/S012567/1).

RS is part-funded by: i) the National Institute for Health Research (NIHR) Biomedical Research Centre at the South London and Maudsley NHS Foundation Trust and King's College London; ii) a Medical Research Council (MRC) Mental Health Data Pathfinder Award to King's College London; iii) an NIHR Senior Investigator Award; iv) the National Institute for Health Research (NIHR) Applied Research Collaboration South London (NIHR ARC South London) at King's College Hospital NHS Foundation Trust.

SH is part-funded by the National Institute for Health Research (NIHR) Biomedical Research Centre at the South London and Maudsley NHS Foundation Trust and King's College London; ESRC Centre for Society and Mental Health at King's College London (ESRC Reference: ES/S012567/1); and the Wellcome Trust (203380Z16Z).

The funders had no role in the study design, data collection and analysis, decision to publish, or preparation of the manuscript. The views expressed are those of the author(s) and not necessarily those of the NHS, the NIHR, the MRC, the Department of Health, the ESRC or King's College London.

COMPETING INTERESTS STATEMENT

All authors have confirmed that they have no competing interests to declare.

CHECKLIST/FLOW DIAGRAM FOR REPORTING STATEMENT

For peer review only - http://bmjopen.bmj.com/site/about/guidelines.xhtml

Figure 3 is a flow-diagram illustrating the cohort selection process, in line with the RECORD Statement (https://journals.plos.org/plosmedicine/article%3Fid=10.1371/journal.pmed.1001885).

ETHICS

The SLaM Case Register has been approved as a source of de-identified data for secondary analyses (Oxford Research Ethics Committee C, reference 18/SC/0372).

DATA SHARING STATEMENT

We are unable to place test data in the public domain because these comprise patient information, but document IDs used in application development and testing have been archived and researchers may apply for approval to access these or other CRIS data. This application is also being put into production for researchers to use in the Biomedical Research Centre. More information can be found at http://brc.slam.nhs.uk/about/core-facilities/cris.

PATIENT AND PUBLIC INVOLVEMENT

This study proposal was reviewed and approved by the patient-led CRIS oversight committee prior to the commencement of the project. No other consultations were made with patients or the public during the process of the study.

AUTHOR CONTRIBUTIONS

The study was conceived by JD, AK, RS, AR, SH, BG and LHA. Personal history sections of documents were extracted using an application developed by DC. Manual annotations to develop the annotation guidelines and produce the test and training data were conducted by AK, AR, ET, ZC and KB, and SM (acknowledgements). The application was developed by XS, with feedback from AK, NC, JD, RS, AR, and SH. The application was implemented over the EHR by DC and JS. The application was evaluated by AK and NC. The missing data analysis was conducted by NC and JD. The paper draft was led by NC, JD and AK; and was critically reviewed and edited by all authors (AK, XS, AR, ET, RS, ZC, KB, DC, JS, BG, LHA, SH).

ACKNOLWEDGEMENTS

For peer review only - http://bmjopen.bmj.com/site/about/guidelines.xhtml

We appreciated the technical support from informatics personnel in the NIHR Maudsley Biomedical Research Centre and the University of Sheffield. We would also like to thank Shirlee MacCrimmon for her assistance with annotations during the annotation guideline development process.

REFERENCES

1. Luciano, A. and E. Meara, *Employment status of people with mental illness: national survey data from 2009 and 2010.* Psychiatric Services, 2014. **65**(10): p. 1201-1209.
2. Marwaha, S., et al., *Rates and correlates of employment in people with schizophrenia in the UK, France and Germany.* The British Journal of Psychiatry, 2007. **191**(1): p. 30-37.
3. Dunn, E.C., N.J. Wewiorski, and E.S. Rogers, *The meaning and importance of employment to people in recovery from serious mental illness: results of a qualitative study.* Psychiatric rehabilitation journal, 2008. **32**(1): p. 59.
4. Marwaha, S. and S. Johnson, *Schizophrenia and employment.* Social psychiatry and psychiatric epidemiology, 2004. **39**(5): p. 337-349.
5. Moscone, F., E. Tosetti, and G. Vittadini, *The impact of precarious employment on mental health: The case of Italy.* Social Science & Medicine, 2016. **158**: p. 86-95.
6. Luciano, A., G.R. Bond, and R.E. Drake, *Does employment alter the course and outcome of schizophrenia and other severe mental illnesses? A systematic review of longitudinal research.* Schizophrenia research, 2014. **159**(2-3): p. 312-321.
7. Gilbert, E. and S. Marwaha, *Predictors of employment in bipolar disorder: a systematic review.* Journal of Affective Disorders, 2013. **145**(2): p. 156-164.
8. Schofield, P. and J. Das-Munshi, *Big data: what it can and cannot achieve.* BJPsych Advances, 2018. **24**(4): p. 237-244.
9. Aldekhyyel, R., et al. *Content and quality of free-text occupation documentation in the electronic health record*. in *AMIA Annual Symposium Proceedings*. 2016. American Medical Informatics Association.
10. Connelly, R., V. Gayle, and P.S. Lambert, *A review of occupation-based social classifications for social survey research.* Methodological Innovations, 2016. **9**: p. 2059799116638003.
11. Lovis, C., R.H. Baud, and P. Planche, *Power of expression in the electronic patient record: structured data or narrative text?* International Journal of Medical Informatics, 2000. **58**: p. 101-110.
12. Wu, C.-Y., et al., *Evaluation of smoking status identification using electronic health records and open-text information in a large mental health case register.* PloS one, 2013. **8**(9): p. e74262.
13. Jackson, R.G., et al., *Natural language processing to extract symptoms of severe mental illness from clinical text: the Clinical Record Interactive Search Comprehensive Data Extraction (CRIS-CODE) project.* BMJ open, 2017. **7**(1): p. e012012.
14. Iqbal, E., et al., *Identification of adverse drug events from free text electronic patient records and information in a large mental health case register.* PloS one, 2015. **10**(8): p. e0134208.
15. Chandran, D., et al., *Use of Natural Language Processing to identify Obsessive Compulsive Symptoms in patients with schizophrenia, schizoaffective disorder or bipolar disorder.* Scientific reports, 2019. **9**(1): p. 1-7.
16. Fernandes, A.C., et al., *Identifying suicide ideation and suicidal attempts in a psychiatric clinical research database using natural language processing.* Scientific reports, 2018. **8**(1): p. 1-10.
17. Perera, G., et al., *Cohort profile of the South London and Maudsley NHS Foundation Trust Biomedical Research Centre (SLaM BRC) case register: current status and recent enhancement of an electronic mental health record-derived data resource.* BMJ open, 2016. **6**(3): p. e008721.

For peer review only - http://bmjopen.bmj.com/site/about/guidelines.xhtml

18. Cunningham, H., et al., *Getting more out of biomedical documents with GATE's full lifecycle open source text analytics.* PLoS computational biology, 2013. **9**(2): p. e1002854.
19. Viani, N., et al. *Time Expressions in Mental Health Records for Symptom Onset Extraction*. in *Proceedings of the Ninth International Workshop on Health Text Mining and Information Analysis*. 2018.
20. Hollister, B.M., et al. *Development and performance of text-mining algorithms to extract socioeconomic status from de-identified electronic health records*. in *PACIFIC SYMPOSIUM ON BIOCOMPUTING 2017*. 2017. World Scientific.
21. Yang, H. and J.M. Garibaldi, *Automatic detection of protected health information from clinic narratives.* Journal of biomedical informatics, 2015. **58**: p. S30-S38.
22. Dehghan, A., et al. *Identification of occupation mentions in clinical narratives*. in *International Conference on Applications of Natural Language to Information Systems*. 2016. Springer.
23. Chilman, N.G.M., & Das-Munshi, J., *Sociodemographic predictors of unemployment in patients with severe mental illness: an electronic health record cohort study.* . Retrieved from osf.io/rx7zs, 2020.

For peer review only

Figure 1 A step-by-step illustration of the methods used for the occupation application development and evaluation, with the number and types of documents used at each step.

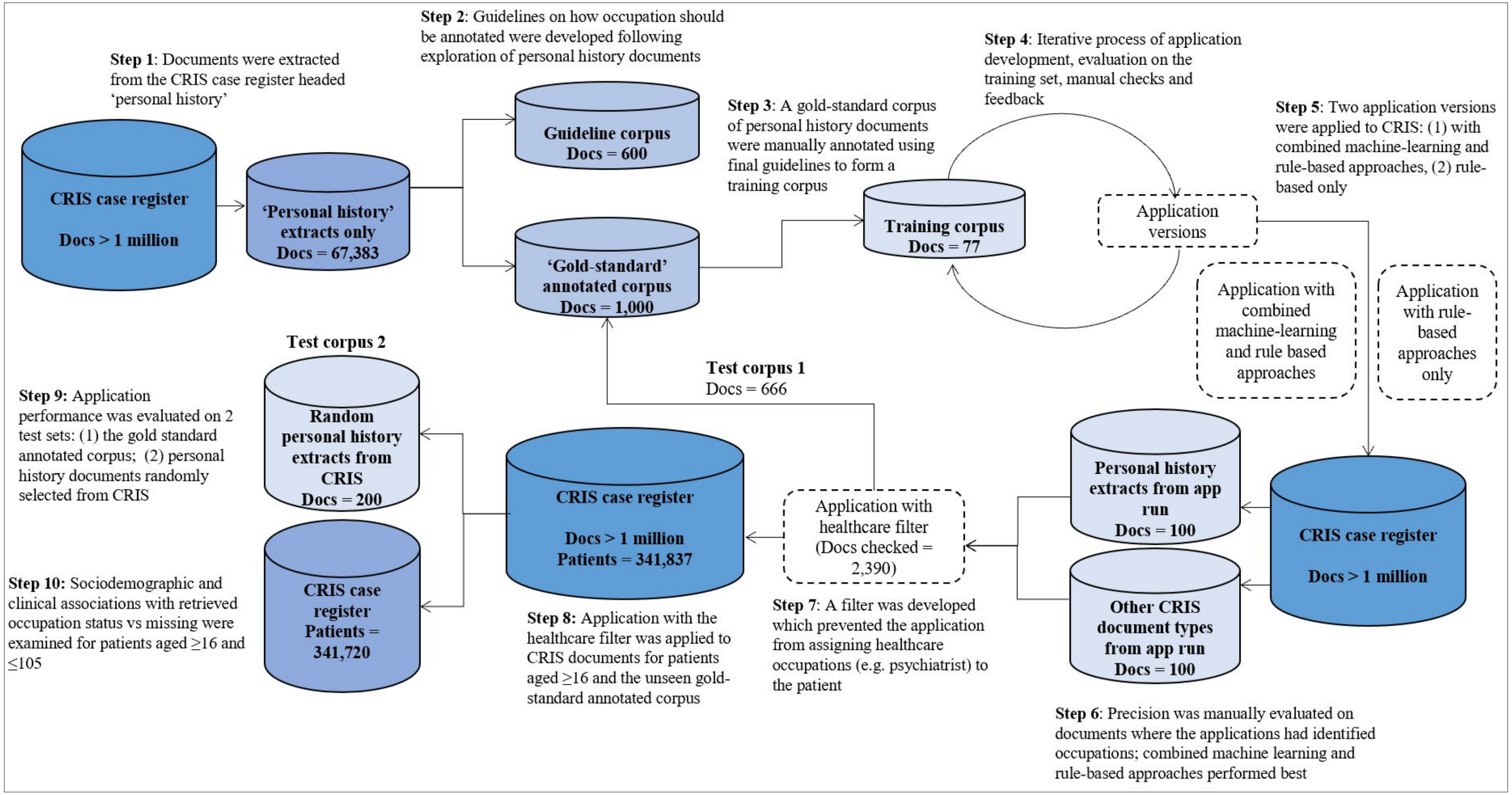

Figure 2 The process undertaken by the application when extracting occupations from the free-text in the Clinical Record Interactive Search (CRIS) case register.

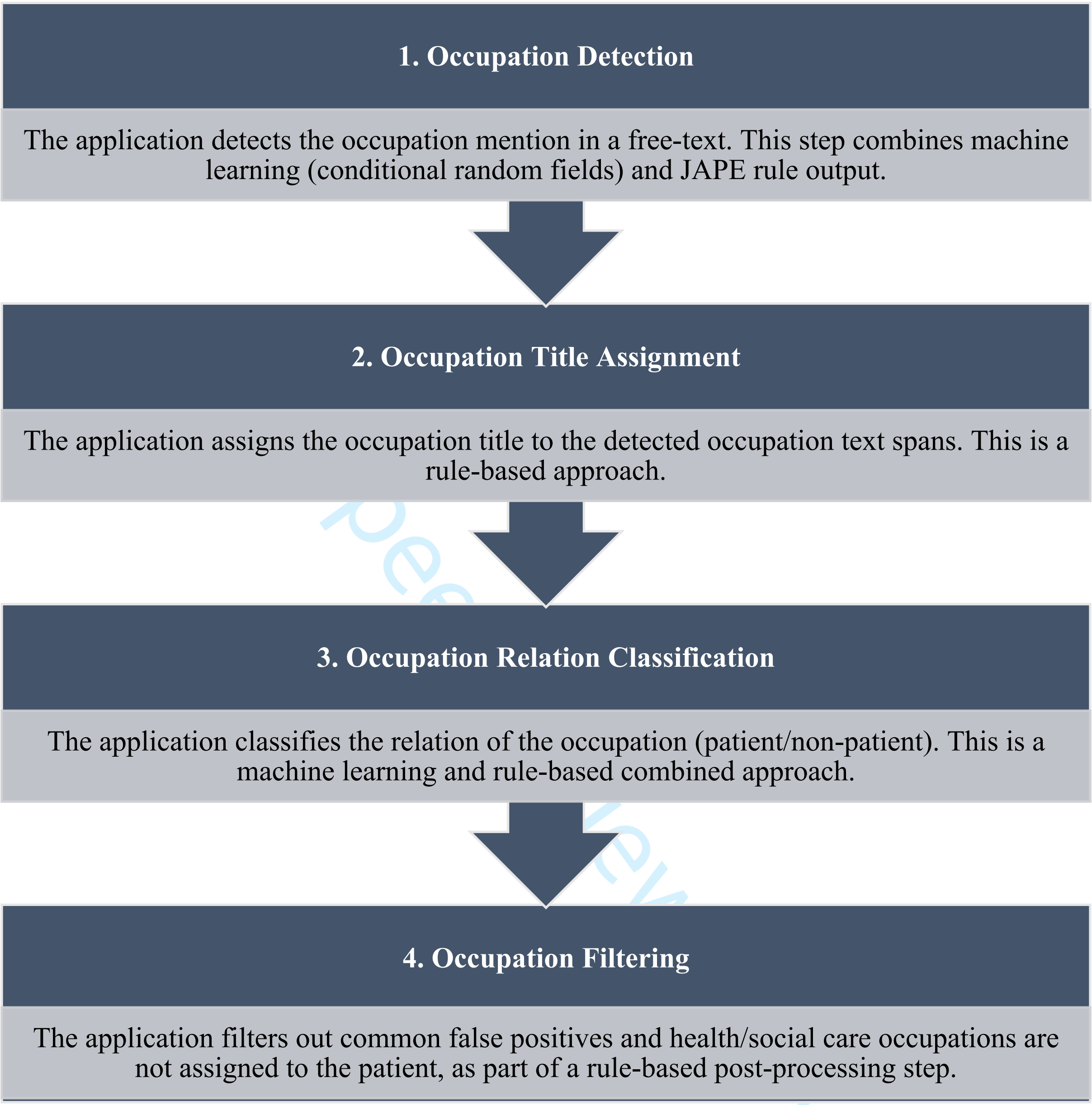

For peer review only - http://bmjopen.bmj.com/site/about/guidelines.xhtml

Figure 3 The study population selection process and extraction results from text-mining occupations over the Clinical Record Interactive Search (CRIS) case register.

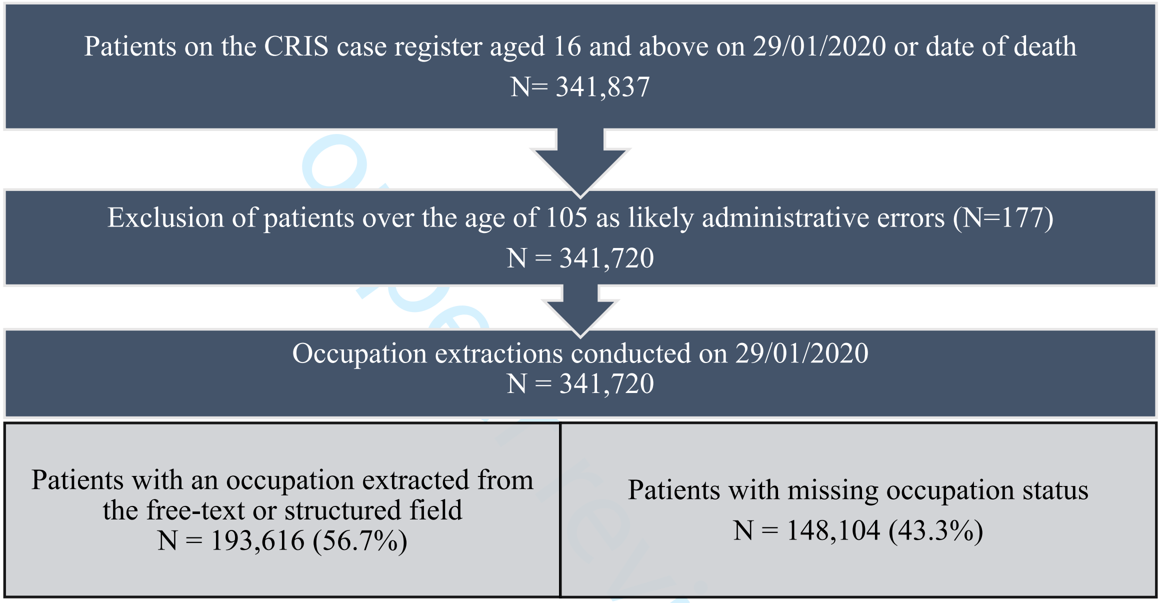

For peer review only

For peer review only - http://bmjopen.bmj.com/site/about/guidelines.xhtml

Table 1 Sociodemographic and clinical features of the Clinical Record Interactive Search (CRIS) case register.*

	**No. patients, % (Total N=341,720)**
**AGE**	
16-29	84,181 (24.63%)
30-49	123,216 (36.06%)
50-69	79,880 (23.38%)
70-89	43,852 (12.83%)
90+	10,591 (3.1%)
**GENDER**	
Male	166,480 (48.72%)
Female	175,007 (51.21%)
Other/Not Known	233 (0.07%)
**ETHNICITY**	
White British	136,289 (39.88%)
Irish	5,182 (1.70%)
Black Caribbean	34,229 (10.02%)
Black African	15,654 (4.58%)
Indian	4,345 (1.27%)
Pakistani	1,852 (0.54%)
Bangladeshi	1,088 (0.32%)
Chinese	1,124 (0.33%)
Other Asian	5,500 (1.61%)
Other Ethnic Group	19,650 (5.75%)
Other White	22,076 (6.46%)
Mixed	1,879 (0.55%)
Not Known	92,222 (26.99%)
**MARITAL STATUS**	
Married/civil partnership/cohabiting	46,617 (13.64%)
Divorced/separated/civil partnership dissolved	17,309 (5.07%)
Widowed	15,758 (4.61%)
Single	141,111 (41.29%)
Not Known	120,925 (35.39%)
**LOCAL QUARTILES OF NEIGHBOURHOOD DEPRIVATION**	
Least deprived	79,537 (23.28%)
3rd Quartile	80,049 (23.43%)
2nd Quartile	79,767 (23.34%)
Most deprived	79,829 (23.36%)
Address Not Known	22,538 (6.60%)
**PRIMARY DIAGNOSIS**	
F30-F39: mood (affective) disorders	37,796 (11.06%)
F00-F09: organic, including symptomatic, mental disorders	29,801 (8.72%)
F10-F19: mental and behavioural disorders due to psychoactive substance misuse	27,870 (8.16%)

For peer review only - http://bmjopen.bmj.com/site/about/guidelines.xhtml

F20-F29: schizophrenia, schizotypal and delusional disorders	18,253 (5.34%)
F40-F49: neurotic, stress-related and somatoform disorders	31,962 (9.35%)
F50-F59: behavioural syndromes associated with physiological disturbances and physical factors	9,166 (2.68%)
F60-F69: disorders of adult personality and behaviour	6,605 (1.93%)
F70-F79: mental retardation	2,732 (0.80%)
F80-F89: disorders of psychological development	5,874 (1.72%)
F90-F98: behavioural and emotional disorders with onset usually occurring in childhood and adolescence	12,028 (3.52%)
Other diagnosis	83,847 (24.54%)
Not Known	75,786 (22.18%)
**QUARTILES OF 'EVENTS' ENTERED INTO THE HEALTH RECORD**	
No Events	50,673 (14.83%)
Least Events (1-3)	86,818 (25.41%)
2nd Quartile (4-10)	62,804 (18.38%)
3rd Quartile (11-40)	68,774 (20.13%)
Most Events (41+)	72.651 (21.26%)
**INPATIENT BED DAYS**	
No inpatient admissions	311,099 (91.04%)
Low (1-2 days)	1,937 (0.50%)
Moderate (3-31 days)	10,587 (3,10%)
High (32+ days)	18,337 (5.37%)
**At the time of occupation application run (29.01.2020).*	

For peer review only - http://bmjopen.bmj.com/site/about/guidelines.xhtml

*Table 2 Results from crude and multivariable logistic regression analyses examining predictors of occupation recording from the Clinical Record Interactive Search (CRIS) case register. **

	**N (%) with at least one occupation retrieved by structured field/text-mining extractions**	**OR (95% CI)**	**aOR[1] (95% CI)**	**aOR[2] (95% CI)**
**AGE**				
16-29	41,653 (49.48)	Reference	Reference	Reference
30-49	68,422 (55.53%)	**1.27 (1.25-1.30)**	**1.56 (1.53-1.59)**	**1.72 (1.68-1.75)**
50-69	49,289 (61.70%)	**1.65 (1.61-1.68)**	**1.98 (1.93-2.02)**	**2.19 (2.14-2.25)**
70-89	27,175 (61.97%)	**1.66 (1.63-1.70)**	**1.71 (1.67-1.76)**	**1.60 (1.54-1.65)**
90+	7,077 (66.82%)	**2.06 (1.97-2.15)**	**2.14 (2.04-2.24)**	**2.00 (1.89-2.11)**
**GENDER**				
Male	96,141 (57.75%)	Reference	Reference	Reference
Female	97,443 (55.68%)	**0.92 (0.91-0.93)**	**0.88 (0.87-0.90)**	**0.87 (0.85-0.88)**
Other/Not Known	32 (13.73%)	**0.12 (0.08-0.17)**	**0.10 (0.07-0.15)**	**0.16 (0.10-0.24)**
**ETHNICITY**				
White British	91,575 (67.19%)	Reference	Reference	Reference
Irish	4,303 (74.04%)	**1.39 (1.31-1.48)**	**1.24 (1.17-1.33)**	**1.23 (1.15-1.31)**
Black Caribbean	24,753 (72.32%)	**1.28 (1.24-1.31)**	0.99 (0.96-1.02)	**1.06 (1.03-1.09)**
Black African	11,341 (72.45%)	**1.28 (1.24-1.33)**	**1.07 (1.03-1.11)**	**1.12 (1.07-1.17)**
Indian	2,876 (66.19%)	0.96 (0.90-1.02)	**0.91 (0.85-0.97)**	**0.91 (0.85-0.98)**
Pakistani	1,185 (63.98%)	**0.87 (0.79-0.95)**	**0.81 (0.73-0.90)**	**0.82 (0.74-0.91)**
Bangladeshi	719 (66.08%)	0.95 (0.84-1.08)	0.90 (0.78-1.03)	0.94 (0.82-1.08)
Chinese	690 (61.39%)	**0.78 (0.69-0.88)**	**0.73 (0.65-0.84)**	**0.81 (0.71-0.92)**
Other Asian	3,543 (64.42%)	**0.88 (0.84-0.94)**	**0.82 (0.78-0.87)**	**0.85 (0.80-0.91)**
Other ethnic Group	11,768 (59.89%)	**0.73 (0.71-0.75)**	**0.77 (0.75-0.80)**	**0.75 (0.72-0.77)**
Other White	14,610 (66.18%)	**0.96 (0.93-0.98)**	**0.94 (0.91-0.97)**	0.97 (0.94-1.00)
Mixed Race	1,197 (63.70%)	**0.86 (0.78-0.94)**	**0.68 (0.61-0.75)**	**0.78 (0.70-0.87)**
Not Known	25,056 (27.17%)	**0.18 (0.18-0.19)**	**0.31 (0.31-0.32)**	**0.50 (0.49-0.51)**

For peer review only - http://bmjopen.bmj.com/site/about/guidelines.xhtml

**MARITAL STATUS**				
Married/Civil Partnership/Cohabiting	31.037 (66.58%)	Reference	Reference	Reference
Divorced/Separated/Civil Partnership Dissolved	13,346 (77.10%)	**1.69 (1.62-1.76)**	**1.47 (1.40-1.53)**	**1.41 (1.35-1.47)**
Widowed	11,309 (71.77%)	**1.28 (1.23-1.33)**	1.05 (1.00-1.09)	**1.05 (1.01-1.10)**
Single	98,841 (70.04%)	**1.17 (1.15-1.20)**	1.02 (1.00-1.05)	**1.24 (1.21-1.27)**
Not Known	39,083 (32.32%)	**0.24 (0.23-0.25)**	**0.33 (0.32-0.33)**	**0.49 (0.47-0.50)**
**LOCAL QUARTILES OF NEIGHBOURHOOD DEPRIVATION**				
Least Deprived	48,155 (60.54%)		Reference	Reference
3rd Quartile	47,583 (59.44%)	**0.96 (0.94-0.97)**	**0.97 (0.95-0.99)**	**0.96 (0.94-0.99)**
2nd Quartile	45,842 (57.47%)	**0.88 (0.86-0.90)**	**0.94 (0.91-0.96)**	**0.93 (0.91-0.95)**
Most Deprived	41,800 (52.36%)	**0.72 (0.70-0.73)**	**0.89 (0.87-0.91)**	**0.88 (0.86-0.90)**
Address Not Known	10,236 (45.42%)	**0.54 (0.53-0.56)**	**0.70 (0.67-0.72)**	**0.77 (0.74-0.80)**
**DIAGNOSIS**				
F30-F39: mood (affective) disorders	27,057 (71.59%)	Reference	Reference	Reference
F00-F09: organic, including symptomatic, mental disorders	20,269 (68.01%)	**0.84 (0.82-0.87)**	**0.91 (0.88-0.94)**	**0.71 (0.68-0.74)**
F10-F19: mental and behavioural disorders due to psychoactive substance misuse	18,150 (65.12%)	**0.74 (0.72-0.77)**	**0.71 (0.68-0.73)**	**0.47 (0.45-0.49)**
F20-F29: schizophrenia, schizotypal and delusional disorders	14,645 (80.23%)	**1.61 (1.54-1.68)**	**0.87 (0.83-0.91)**	**0.78 (0.74-0.82)**
F40-F49: neurotic, stress-related and somatoform disorders	19,920 (62.32%)	**0.66 (0.64-0.68)**	**0.75 (0.72-0.77)**	**0.76 (0.73-0.79)**
F50-F59: behavioural syndromes associated with physiological disturbances and physical factors	5,287 (57.68%)	**0.54 (0.52-0.57)**	**0.65 (0.62-0.68)**	**0.68 (0.64-0.72)**

For peer review only - http://bmjopen.bmj.com/site/about/guidelines.xhtml

F60-F69: disorders of adult personality and behaviour	4,739 (71.75%)	1.01 (0.95-1.07)	**0.68 (0.64-0.73)**	**0.77 (0.72-0.82)**
F70-F79: mental retardation	2,277 (83.35%)	**1.99 (1.79-2.20)**	**1.81 (1.63-2.03)**	**1.69 (1.51-1.90)**
F80-F89: disorders of psychological development	4,377 (74.78%)	**1.16 (1.09-1.24)**	**1.22 (1.14-1.30)**	**1.78 (1.66-1.92)**
F90-F98: behavioural and emotional disorders with onset usually occurring in childhood and adolescence	8,754 (72.78%)	**1.06 (1.01-1.11)**	**1.25 (1.19-1.32)**	**1.84 (1.74-1.93)**
Other diagnosis	43,787 (52.22%)	**0.43 (0.42-0.45)**	**(0.68-0.72)**	**0.76 (0.73-0.78)**
Not Known	24,354 (32.14%)	**0.19 (0.18-0.19)**	**0.44 (0.43-0.45)**	**0.66 (0.64-0.68)**
**QUARTILES OF 'EVENTS' ENTERED INTO THE HEALTH RECORD**				
No Events	12,012 (23.70%)	Reference	Reference	Reference
Least Events	35,009 (40.32%)	**2.17 (2.12-2.23)**	**2.18 (2.13-2.23)**	**1.75 (1.70-1.79)**
2nd Quartile	34,368 (54.72%)	**3.89 (3.79-3.99)**	**3.89 (3.79-3.99)**	**2.79 (2.71-2.87)**
3rd Quartile	49,237 (71.59%)	**8.11 (7.90-8.33)**	**8.06 (7.85-8.28)**	**5.01 (4.86-5.16)**
Most Events	62,990 (86.70%)	**20.98 (20.37-21.60)**	**18.89 (18.29-19.50)**	**9.77 (9.43-10.1)**
**INPATIENT BED DAYS**				
No inpatient admissions	167,213 (53.75%)	Reference	Reference	Reference
Low (1-2 days)	1,408 (82.97%)	**4.19 (3.69-4.76)**	**1.87 (1.64-2.14)**	**1.68 (1.47-1.93)**
Moderate (3-31 days)	8,714 (82.31%)	**4 (3.81-4.21)**	1.06 (1.00-1.11)	1.01 (0.95-1.07)
High (32+ days)	16,281 (88.79%)	**6.81 (6.51-7.14)**	**1.57 (1.49-1.66)**	**1.32 (1.25-1.39)**

*All variables listed in this table had a strong association with the outcome variable (p<.0001), assessed by likelihood ratio tests.
[1]Adjusted for service contact variables (no. of events and inpatient bed days)
[2]Adjusted for all other variables in the table

For peer review only - http://bmjopen.bmj.com/site/about/guidelines.xhtml

Supplementary File 1: Descriptions of the datasets used in the development, testing and implementation of the occupation application

Application Development and Testing Datasets			
	**Type of document**	**Document count**	**No. of Occupation Annotations (manual)**
Training corpus	Personal history	77	405
Testing corpus 1: with vs without machine-learning comparison	Personal history + other CRIS documents	200	521
Testing corpus 2: gold-standard annotated documents	Personal history	666	3,429
Testing corpus 4: Unannotated documents	Personal history	200	442
**Application Implementation Dataset**			
	Type of document	**Patient count**	**No. Of Occupation Extractions (application)**
CRIS case register of patient records aged >=16	Attachments	341,720	21,321,757 (all relations)
	Events		
	Correspondence		
	Discharge Notification Summaries		
	History		
	Mental State Formulations		

For peer review only

	Presenting Circumstances		
	Risk Events		
	Social Situation		
	Ward Progress Notes		

Table 1: Descriptions of the datasets used in the development, testing and implementation of the occupation application

For peer review only

OCCUPATION ANNOTATION GUIDELINES

Authors:

Background – Natasha Chilman & Esther Tolani

Annotation rules – Esther Tolani, Angus Roberts, Zoe Chui, Karen Birnie, Lisa Harber-Aschan, Billy Gazard, Anna Kolliakou & Jayati Das-Munshi

General Tips – Esther Tolani

Appendices – Natasha Chilman & Anna Kolliakou

With thanks to Shirlee MaCrimmon for annotation support.

Supplementary File 2 to accompany the BMJ Open paper, "Text-mining occupations from the mental health electronic health record: a natural language processing approach using the Clinical Record Interactive Search (CRIS) platform to identify patient occupations in South London, UK."

Background 2
Setting 2
Development 2
Annotation rules 5
Annotation rules 6
Occupation 8
Subject of Occupation 14
Exclusion Criteria 15
General Tips 16
References 17
Appendix 1 17
A timeline of actions leading to guideline and application development 17
Appendix 2 22
Annotation Guidelines Version 1 22

For peer review only

For peer review only - http://bmjopen.bmj.com/site/about/guidelines.xhtml

Background

The CRIS-occupation-application has been developed to enable researchers to extract occupations from the Clinical Record Interactive Search (CRIS) case register. When using the occupation application, it is important to consider how it has been trained and tested to allow for appropriate use of the application and accurate interpretation of results. These guidelines provide clear and transparent rules which specify how occupations should be annotated manually in free-text EHRs, which then informed the development of the occupation application, and a gold-standard against which the application was evaluated against.

Setting

These occupation annotation guidelines were developed over the years 2017-2020 for use on psychiatric clinical texted accessed through the Clinical Record Interactive Search (CRIS) application. CRIS is a large de-identified case register of electronic health records, comprising of the Electronic Patient Health Journal notes used in South London and Maudsley NHS Trust (SLaM). SLaM is the largest unit mental health provider of secondary services in Europe, serving 1.3 million people across the London boroughs of Lambeth, Southwark, Lewisham and Croydon. The SLaM CRIS case register stores over 350,000 patient records to date, and encompasses a range of secondary mental health services (including inpatient and community mental health services) [1]. Whilst this annotation guideline was written following the exploration of CRIS text extracts, we also recommend that the guidelines can be used as a starting point when extracting occupations from other CRIS systems and psychiatric Electronic Health Records (EHRs) in the UK.

It is important to remember that EHRs are a secondary routine data resource in research, they are used primarily for a practical purpose by clinicians to document patient-level information. This context should be kept in mind when considering the complexity of annotating occupations.

Development

Here we summarise the actions taken to develop the guidelines and describe how the guidelines have changed over time. This is also detailed further in the development timeline (Appendix 1).

These guidelines were based on the 'personal history' sections of the free-text entries. When clinicians use 'personal history' as a header in the free-text fields in CRIS, the text which follows typically includes information on the patient's upbringing and family life,

For peer review only - http://bmjopen.bmj.com/site/about/guidelines.xhtml

education and – most importantly for our interests – occupation. Personal history sections were chosen as the best place to start when examining how occupation is described in the free-text fields in CRIS. An application previously developed by Dr David Chandran in the Biomedical Research Centre was used to extract personal history documents from CRIS to develop and test the annotation guidelines. A 'document' is a single section of a free-text field in CRIS, for example a letter attachment or event progress note. One patient may have more than one personal history section in their record.

Initial guidelines were drawn from the exploration of 100 personal history documents and team discussions. From the first draft, the occupation annotation guidelines were developed based on the premise that when an occupation is annotated in the free-text, two components must be specified: the occupation (feature) and subject of the occupation (relation). Occupation is a complex concept and can be written as a job title (e.g. a waiter) or a description of a work activity (e.g. serving tables).

The guidelines were developed through an iterative process of document annotation, team discussions and rule development (Appendix 1). 600 personal history documents were annotated throughout this process which informed and tested the sufficiency of the guidelines to instruct occupation annotation. Out of these 600 documents, 250 personal history documents were double annotated. Inter-annotator agreements were calculated throughout the guideline development stages to assess whether the guidelines were sufficient for occupation to be annotated consistently (Appendix 1). By November 2017, 200 further personal history documents were double-annotated with good inter-rater reliability between two manual annotators, with a Cohen's Kappa statistic of 77% for occupation and 72% for relation. This is considered a good level of agreement. 800 documents were then annotated by a single annotator using the latest guideline and together these formed the 1000 document gold-standard annotated document corpus. This corpus was later used for application development (forming the training corpus).

To demonstrate how the guidelines have changed over time, please see Appendix 2 which shows the Guidelines Version 1 (GV1). When compared to the current guidelines, a significant level of detail has been added since the initial draft. For example, there is now a section the beginning of the guidelines stating which parts of a sentence describing occupation should be annotated. Whilst re-drafting the guidelines, an 'additional information' column was added to give further detail on how the annotation rules work, which researchers found helpful when completing annotations. The later drafts of the guidelines also add a 'blank' annotation rule: if the occupation title can be inferred by the text itself then the occupation feature should be left empty, and the relation was determined by the sentence structure. This was important when later evaluating the application, as a bespoke GATE evaluation package was used to take this rule into account. All changes that were made to the annotation guideline throughout the development process were agreed within the research team.

For peer review only - http://bmjopen.bmj.com/site/about/guidelines.xhtml

The following guideline is the final annotation guideline document. Whilst there were some small formatting changes made during application development (Appendix 1), the rules in this guideline were used when annotating the 1000 gold-standard training and testing corpus for the application.

For peer review only

For peer review only - http://bmjopen.bmj.com/site/about/guidelines.xhtml

Annotation rules

Esther Tolani, Zoe Chui, Karen Birnie, Angus Roberts, Anna Kolliakou, Jayati Das-Munshi, Robert Stewart

These guidelines outline the process for annotating occupation status in GATE. The term(s) highlighted should be the word(s) in the free text which indicate(s) the occupation of an individual. After reading the free text, annotations should be made on the word(s) which is (are) related to an employment status or an occupation: job or profession. For all cases, each annotation will have the following features: **occupation and subject of occupation (relation).**

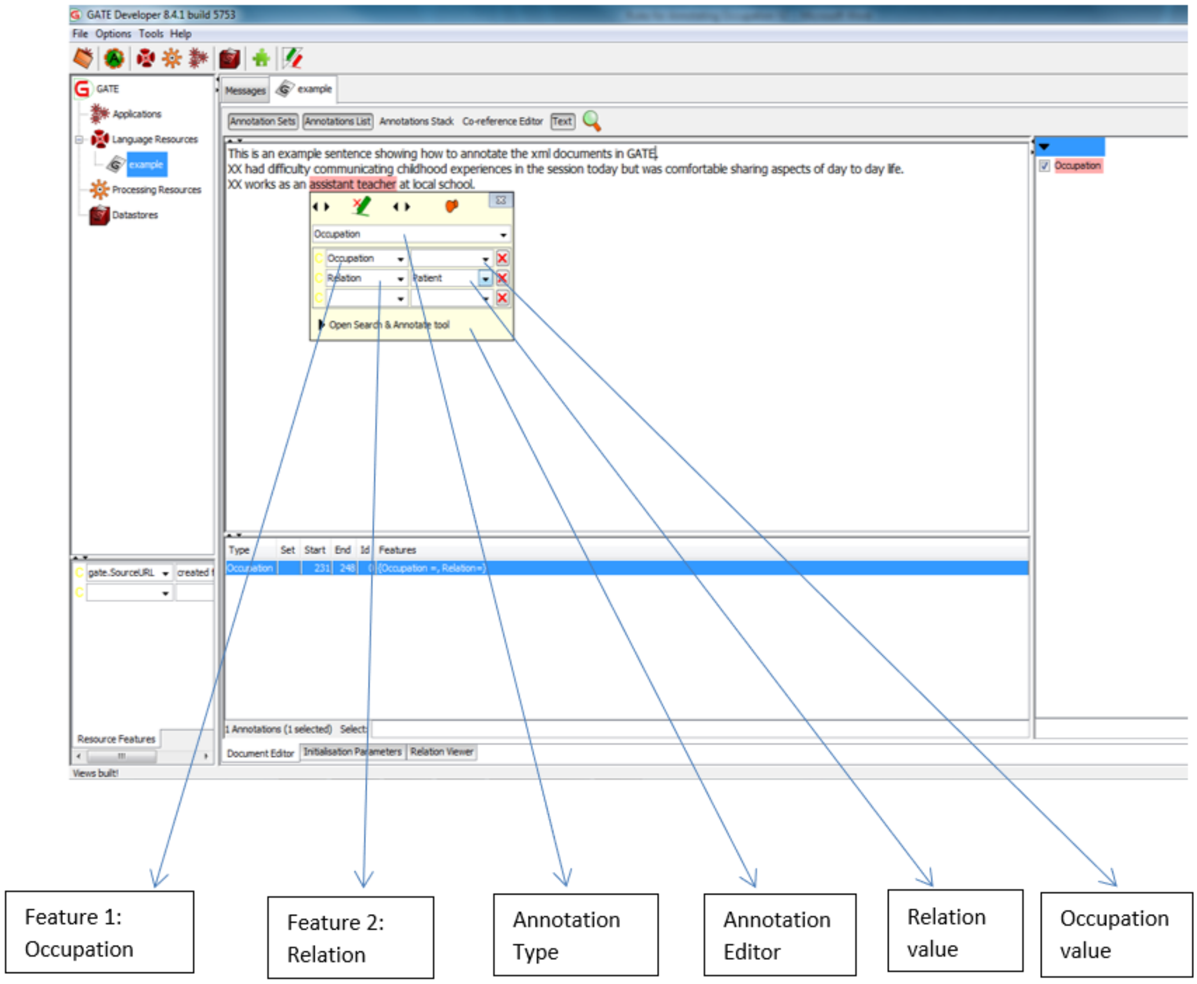

Figure 1: A labelled example illustrating how occupation is annotated in GATE software

Sentence Structure of Annotation

The annotation should be made on adjectives, nouns and verbs in the sentence.

- Title of Occupation

Titles of occupations are always nouns.

For peer review only - http://bmjopen.bmj.com/site/about/guidelines.xhtml

Adjectives should only be annotated when they are part of the occupation type or necessary for describing the occupation e.g. assistant manager, senior consultant. The annotation value is left empty when occupation can be inferred from the exact annotated text.

Example:
XX worked as an assistant teacher – occupation value: empty.
She is a mental health nurse – occupation value: empty
Annotate the adjective and noun.

- **Description of Occupation**
  A) Description of occupation consists of verbs referring to work activities.

Annotate text following:
  1) Works for/in/as/at…

Works for real estate - occupation value: estate agent
Works for British Gas - occupation value: British Gas worker
Works for investment bank – occupation value: investment bank worker

  2) Job/Role involves, has to do with, includes…

Job involves cleaning houses – occupation value: house cleaner
Role involves writing, teaching – occupation value: writer, teacher

  3) Verbs indicating membership

Joined the navy – occupation value: navy officer
Example:
XX worked joined the army after moved to the UK – occupation value: army officer.
Annotate the verb and noun because the noun or verb alone does not describe the occupation sufficiently.

Annotation rules

An occupation or description of work should be annotated regardless of whether it is current or past. However, text indicating whether occupation or description of work is current or past is not required for the annotation unless it offers information on the stability/transience of the occupation.

Examples:
XX is not working at the moment – occupation value: unemployed
XXX has been working as a chef for 3 years- occupation value: chef

For peer review only - http://bmjopen.bmj.com/site/about/guidelines.xhtml

XXX worked briefly or worked for a few months or worked every summer – occupation value: other

Do not annotate:

- Punctuation
    - e.g. full stops, semi-colons…
- Adverbs
    - e.g. happily, works hard…
- Articles in front of occupation
    - e.g. the, as, an, a…
- Conjunctions
    - e.g. and, but, if…

*[**UNLESS** these are articles and conjunctions in a double annotation as further below]*

- Adjectives when describing a quality assigned to a job
    - e.g. experienced teacher, qualified electrician
- Verbs that precede title of occupation
    - e.g. became, moved to, promoted to, went to, decided to, etc.
- Text around title of occupation describing place of work **unless** text around title of occupation refers to a field or sector
    - e.g. assistant manager for a phone company – value empty
    - e.g. assistant manager in sales – value fill Sales Assistant Manager
- Time frames or duration of work
    - e.g. worked for 5 years, was a chef in 1995, has worked, is not working

*[**UNLESS** it offers information on the stability/transience of the occupation ie worked briefly or worked for a few months or worked every summer]*

Double annotation:

In the case of two joint occupation descriptions, annotate the same text twice and give a different value each time.

Examples:
Annotate once: he worked in a clothes shop and a kitchen – occupation value: retail worker
Annotate twice: he worked in a clothes shop and a kitchen – occupation value: other - kitchen

Please use this double annotation as sparingly as possible and not when clearly stated occupations or different occupations/work descriptions are joined as below.

Examples:
He worked as a chef and cleaner – two annotations with blank values

For peer review only - http://bmjopen.bmj.com/site/about/guidelines.xhtml

He worked on building sites and roofing - two annotations with first value 'labourer' and second 'labourer' or 'other'.

Long job descriptions:
Sometimes clinical record notes are written in a rich and speech-like manner. In cases like this, it is best to annotate a longer piece of text then risk leaving out valuable information.

Examples:
She has worked for only 1 and half year in her life in a wine bar 28yrs ago – occupation value: other- bar

He used to work every summer with his brother at a car wash – occupation value: other-car wash

Occupation

For the occupation value, a title for the work described should be entered. If no title can be created from a work description, 'other' should be entered in the occupation value. In addition, if the title is identical to the work described (job can be inferred from the annotated text), the occupation should be left empty.

Rules for annotating Employment Status				
Rule	Rule Description	Example	Occupation Value	Additional Information
Description of job is given, without job title	Annotate with closely related description	Daily role involves operating the machines	Machine operator	
Multiple occupations	All occupations mentioned in the free text (personal history) should be annotated even if they are or appear to be repetitions of an occupation already mentioned within the same history	Chef, 7.5-tonne truck driver<br><br>Worked as kitchen assistant…he helped in a kitchen for 6 months<br><br>She was a teacher…enjoyed her work as a teacher	[blank]<br>[blank]<br><br>Kitchen assistant<br>Other-kitchen<br><br>[blank]<br>[blank]	For chef, truck driver, kitchen assistant and teacher the occupation value should be left empty because the work descriptions are identical to the title that should be given. For 'helped in a kitchen' the occupation value should be 'other'
Related occupations	Annotate all occupations which are mentioned which are	Worked as a social worker and later became a manager	[blank]<br>[blank]	The occupation value should be left empty because the work

For peer review only - http://bmjopen.bmj.com/site/about/guidelines.xhtml

	associated with the progress of the same job			descriptions social worker and manager identical to the titles that should be given
Place, sector or employer is mentioned without occupation	Annotate the company or sector	XX works for the council<br>He has been with his present boss for a while	Council worker<br><br>Other	Annotate the company, sector or employer
Loose description of job role which cannot be titled	Annotate the reference to odd jobs which have relevance	XX does various jobs which include, tiling, plumbing…	Tiler, plumber	Annotate the word referring to the odd job

For peer review only

The section below outlines how to annotate the alternative employment statuses: student, retired, self-employed, unemployed, carer, homemaker and other.

**Rules for annotating Student Status**				
Rule	Rule Description	Example	Occupation Value	Additional Information
Student (full time/part time)	Annotate term student or a description of full time/ part time study. Include training/vocational courses.	XX is currently studying XX at university<br><br>He trained as a bricklayer<br><br>He trained in art<br><br>She attended university<br><br>Has a degree in Physics<br><br>He did a Masters in Psychology<br><br>Left University in 1995<br><br>Graduated with a degree in maths<br><br>He undertook the early career researcher training scheme	Student<br><br>Student [blank]<br><br>Student<br><br>Student<br><br>Student<br><br>Student<br><br>Student<br><br>Student<br><br>Student	Two annotations are made to capture student status and occupation value of empty for bricklayer<br><br>'Trained' is annotated by itself whereas 'attended', 'did' 'degree' or 'undertook' need extra information annotated because out of context they wouldn't be sufficient by themselves
**Rules for annotating Retired Status**				
Retirement	Annotate the term retired or description of retirement	Worked until retirement	Retired	

For peer review only

Rules for annotating Self-Employed Status				
Self-employed without job description	Annotate the term or description of self-employed	Patient is self-employed	[blank]	The occupation value should be left empty because the job title self-employed is stated
		He owns a number of properties and shops	Self-employed	The occupation value should be self-employed. One annotation.
Self-employed with job description or business/property owner	Annotate the term or description of self-employed and job description	Patient is a self-employed builder	[blank] [blank]	These should be two separate annotations (self-employed and builder). Occupation values should be left empty because the job titles self-employed and builder are stated
		He owns a number of properties and shops	Self-employed	One annotation

Rules for annotating Other Employment Status				
Difficult to define or job/ job role not stated<br>Simple reference to work	Annotate the verb ‘work’ or the noun ‘job’	Works occasionally on weekends<br>Has had a few other jobs<br>He worked there for 4 years and then left<br>He worked in 1995<br>He worked hard all his life<br>He worked briefly when younger	Other	Annotate the verb work by itself unless followed by an adverb providing more information about the work itself ie occasionally, the number of jobs ie numerous or the quality ie hard, creative

For peer review only

		He had a satisfactory job<br>She had numerous jobs<br>He has a creative job<br>She has had three other jobs<br>He did about 8 jobs		
Sector is not mentioned	Statements not referring to a specific sector or industry should not be annotated	XX moved to the private sector	Other-private	
Army/Navy occupations	Annotate relevant word/ phrase	XX joined the army	Army officer	Always annotate as army or navy officer
Job or occupation relating to shops	Annotate relevant word/ phrase	XX worked part-time in WHSmith	Retail worker	Always annotate as retail worker
Sector or place of work is mentioned but unclear what job the subject undertook	Annotate relevant word/phrase	XX joined his brother in construction<br><br>XX worked in a kitchen	Other-construction	It is not clear what job in construction the patient did so occupation value is given 'other'
**Rules for annotating Unemployed Status**				
Unemployment	Annotate the term unemployed or the description of unemployment	XX has not worked for several years<br>XXX does not work anymore<br>XXX lost his job<br>XXX ran out of work a year ago<br>XXX is currently not working<br>XXX got sacked<br>XXX was made redundant<br>XXX stopped working<br>XXX cannot remember the	Unemployed	Unemployment is usually stated in various ways. If the word unemployed is annotated, the annotation value should be blank

For peer review only

		last job she had<br>Last job was about 5 years ago<br><br>XXX is unemployed	[blank]	
**Rules for annotating Homemaker Status**				
Housewife househusband	Annotate the term that states that an individual is a homemaker	Mother was a housewife…	[blank]	The occupation value should be left empty because housewife status is stated
**Rules for annotating Carer Status**				
Carer	Annotate the term carer or the description of care role	XX is a carer for elderly mother<br><br>XXX was a carer	Carer<br><br>[blank]	Annotation value of carer should be entered if text annotated includes who the person cared for is. In the second case, where this is not stated, the occupation value is left empty because carer status is stated
**Rules for annotating Volunteer**				
Volunteer	Annotate the noun volunteer or the verb volunteering	XX volunteered with the council once a week	Volunteer	
**Rules for annotating National Service**				
National Service	Annotate the noun national service and the verb preceding	He joined national service<br><br>He did his national service<br><br>He finished national service	Other – national service	
**Rules for annotating illegal activities**				

For peer review only - http://bmjopen.bmj.com/site/about/guidelines.xhtml

Prostitution	Annotate relevant word/phrase	XX was working as a prostitute	Sex-worker	Always annotate as sex-worker
Jobs of questionable status/legality	Text referring to income generating jobs that might not be legal	He was a brothel owner<br><br>She made money from dealing drugs	Other- brothel<br><br>Other – drug dealing	Other plus an indication of place or type of work

Subject of Occupation

The relation value should state who the occupation refers to/who carries out the job described. In most cases, the occupation belongs to the patient. The occupation can also belong to the parent/carer of the patient, spouse, relative or other.

**Rules for annotating Subject of Occupation**				
Rule	Rule Description	Example	Relation Value	Additional Information
Patient	The occupation annotated should belong to the patient	Patient was a butcher for XX years	Patient	
Parent/ Carer / Guardian	The occupation annotated should belong to the father or mother of the patient.	Father works as a mechanic	Father	
Spouse	The occupation annotated should belong to the spouse	Husband works for the government doing research	Spouse	The occupation of the spouse should still be recorded even if the text suggests they are no longer together
Relative	The occupation annotated belongs to a family member of the patient who is not the parent/carer or spouse	XX's brother discussed the issues faced being XX's carer and working as a shop assistant…	Brother	Relations include: sibling, cousin, aunt, uncle, niece, child, nephew, and grandchild
Girlfriend/Boyfriend/ Partner	The occupation annotated should belong to the patient's girlfriend/boyfriend	XX's girlfriend was a carer for the elderly	Girlfriend	
Other	The occupation annotated does not belong to the patient or patient's relative	The nurse came round to see XX	Other	

For peer review only - http://bmjopen.bmj.com/site/about/guidelines.xhtml

Exclusion Criteria

Rule	Rule Description	Example	Value	Additional information
Future Plans	Future plans to work should not be annotated	XX plans to start role	No annotation	
Hypothetical statements	Text referring to hypothetical scenarios or worries about losing job	XX said he would have left his job if he thought he couldn't cope<br><br>XX was worried he was going to get sacked<br><br>She would have quit if they hadn't given her a raise	No annotation<br><br>No annotation<br><br>No annotation	
When 'work' is used as an adjective<br><br>Describing quality of work without explicitly stating having one		She had great work ethics<br><br>Her work performance deteriorated<br><br>She didn't like her work colleague<br><br>Her job was really good<br><br>He didn't enjoy working there	No annotation	

For peer review only

General Tips: THINK LIKE AN OCCUPATION MACHINE!

1) The machine doesn't have any context

We annotate personal history segments which, if rich, give us a good idea of an individual's story. The machine does not have that reference and if, for example, we annotate 'stopped' in "he worked for 5 years and then stopped" as 'unemployed' we are essentially teaching it to recognise the word 'stopped' as referring to unemployment. Imagine what will happen when we run this application all over CRIS! Ask, if unsure - does the machine understand the annotation I have assigned regardless of context? What will happen if it learns to recognise it as such in another context?

2) The machine loves more of the same

You come across a personal history segment that has 'worked' 3 times, 'labourer' 2 times, 'jobs' 4 times and 2 'sacked'. The machine doesn't know that these have been repeated as it has no context. Also, the more 'labourers' it gets fed, the more it will learn to unequivocally recognise them automatically in any context. Annotate them all!

3) The machine is as smart as you

If you feel you are spending too long making annotation decisions or find a rule that is making your annotations inconsistent, the machine will think the same. Ask questions no matter how silly they seem!

For peer review only

References

1. Perera, G., et al., *Cohort profile of the South London and Maudsley NHS Foundation Trust Biomedical Research Centre (SLaM BRC) case register: current status and recent enhancement of an electronic mental health record-derived data resource.* BMJ open, 2016. **6**(3): p. e008721.

Appendix 1

A timeline of actions leading to guideline and application development

Natasha Chilman, Anna Kolliakou

Date	Action	Outcome
July 2017	Preliminary meeting with research team<br><br>Esther annotated 100 personal history extracts with no guidelines<br><br>Feedback on annotations and guideline ideas discussed by research team	Development of GV1 (Guidelines Version 1)
August 2017	Anna M annotated 50 of the above 100 extracts using GV1, recommended changes	Development of GV2
August 2017	Comments given by research team on GV2	Development of…<br>GV2.1<br>GV2.2<br>GV2.3<br>GV2.4
September 2017	1,262 personal history documents extracted:<br>- Esther annotated 500 using GV2.4<br>- Shirlee double-annotated 200 of these using GV2.	Inter-annotator agreement for 200 double-annotated documents: Cohen's Kappa calculated by GATE = 72% for occupation, 87% for relation<br><br>Development of GV2.5
October-November 2017	40 case examples were written by Anna K and annotated by Anna K, Lisa, Billy, Shirlee and Angus.	Collective agreements made on rules<br><br>Started development of GV2.6
November 2017	Above case examples were given to Karen and Zoe who annotated according to GV2.5	Collective agreements made on rules

For peer review only - http://bmjopen.bmj.com/site/about/guidelines.xhtml

		Finished development of GV2.6
November-December 2017	1000 new personal history documents extracted:<br>- Karen annotated all 1000 using GV2.6<br>- Zoe double-annotated 200 of these using GV2.6	Inter-annotator agreement for 200 double-annotated documents:<br>Cohen's Kappa =<br>77% for occupation<br>72% for relation<br><br>In total, 1000 documents = 'gold-standard' annotated corpus
March 2018	GV2.6 was finalised and so was re-named GV3. The 1000 annotated documents were stratified by gender, length of extract, and occupation feature type (labelled as 'other' vs 'non-other' – see guideline for more detail).	77/1000 stratified annotated extracts were sent to Xingyi as the test corpus (University of Sheffield) as the training set for the application
April 2018	Application version 1 (AV1) created by Xingyi, sent to Anna K who manually checked application output on the 77 test corpus and precision, recall and F-measures were calculated by GATE evaluation package, feedback provided to Xingyi on application areas for improvement.	Development of AV2
April 2018	As above: AV2 ran on 77 documents, manually checked and measures calculated by GATE evaluation package, feedback provided.	Development of AV3
June 2018	As above: AV3 ran on 77 documents, manually checked and measures calculated by GATE evaluation package, feedback provided.	Development of AV3.1
August 2018	AV3.1 included three different application versions, all ran on 77 documents, manually checked and measures calculated by GATE evaluation package, feedback provided.	Development of AV3.2
November 2018	AV3.2 (one version) was run on 77 documents. The GATE evaluation package was under-estimating the performance of the application, as it classified that if an occupation feature was 'blank' then it was not labelled correctly. Please see guideline for instructions on use of 'blank' feature annotations. These type of annotations came up often in the text.<br><br>A new evaluation package ('revised' GATE evaluation) was created which correctly identified 'blank' annotations as a hit. This	Development of AV3.3

For peer review only - http://bmjopen.bmj.com/site/about/guidelines.xhtml

	increased the F-measure and was felt to more accurately reflect the application performance when checking the output manually.<br><br>A small formatting change was made to the guideline, creating GV4, but there was no change in rule content.<br><br>Further feedback sent to Xingyi.	
November-December 2018	AV3.3 was run on the 77 documents, manually checked and F-measures calculated by revised GATE evaluation package, feedback sent to Xingyi.<br><br>A decision was made that the 77 documents needed to be re-annotated which was completed by Anna K in December 2018.	AV3.3 was updated
January-February 2019	Updated AV3.3 was run on both newly annotated 77 documents and previously annotated 77 documents. Barely any difference found in impact on F measure (a very small increase: old annotations F=0.890, new annotations F=0.896).<br><br>Updated AV3.3 run on newly annotated 77 documents, manually checked and F measures calculated by revised GATE evaluation package, feedback sent to Xingyi.	Development of AV3.4
April 2019	As the application was performing reasonably well on the 77 personal history documents, AV3.4 was run on the whole of CRIS. Anna K eyeballed the output and sent feedback to Xingyi for areas for improvement.	AV3.4 was updated to two versions: AV3.4(with machine learning) and AV3.4Revised (without machine learning)
June-July 2019	Both AV3.4 and AV3.4Revised were run on whole CRIS. Anna and Natasha manually checked 200 random personal-history-only documents, and 100 random CRIS documents. Areas for application improvement were sent to Xingyi.	Development of AV4
August 2019	AV4(ML) and AV4(Revised) were run on the whole CRIS. Training corpus of 77 documents was used to evaluate application on GATE. Anna and Natasha manually checked 200 random personal history-only documents, and 100 random CRIS documents (test corpus).	Results from performance of both applications on training corpus and test corpus is available in Supplementary File 3. Application reached good levels of performance (precision and recall all >0.79 on a test corpus). The machine learning application

For peer review only - http://bmjopen.bmj.com/site/about/guidelines.xhtml

		performed slightly better so this was chosen over the rule-based approach. However occupation ownership remained an issue, where many of the occupations retrieved belonged to people other than the patient e.g. clinicians. The application did not consistently annotate the relation of the occupation correctly, for example often 'psychiatrist' was annotated as belonging to the patient.
September-November 2019	Following occupation ownership issues identified in the manual evaluation, team meetings were held and it was decided to add an occupation 'filter' to the application. This is a list of occupations which have the most common incorrect relations (e.g. psychiatrist, social worker) – where the application incorrectly annotates the occupation as belonging to the patient. The occupations included in the filter will be assigned a 'other' relation, rather than 'patient' relation. This will mean that we can be more confident that the occupation extracted belongs to the patient. The team reflected that we may miss a small number of true positives this way (e.g. psychiatrists who are patients), but the risk of retrieving incorrect patient occupations is greater, plus healthcare professionals often go to different occupational services for mental health support so are less likely to be included in this sample of electronic health records.<br><br>Method:<br>- Natasha extracted occupations with ≥100 annotations across CRIS. She then sorted these occupations into 3 categories: those which should definitely be added to the filter (e.g. psychiatrist), those which she was not sure about (e.g. interpreter) and those not to add to the filter (e.g. construction).<br>- Out of those which she was not sure about, Natasha checked between 10-40 documents for the number of true positives retrieved by the application (where the occupation was annotated correctly as belonging to the patient).	AV4 with machine learning was updated by Xingyi to include the occupation filter, where the occupations on the filter list were assigned the relation 'other' rather than patient.

For peer review only - http://bmjopen.bmj.com/site/about/guidelines.xhtml

	During this process Natasha checked a total of 2,390 documents.<br>- Jay and Anna then went through this list to make collective decisions with Natasha on the unsure occupations. The filter list of occupations was then sent to research team for approval, then sent to Xingyi to add to the app.	
January-February 2020	The application was run over the whole of CRIS with the health/social care occupation filter applied.	Natasha firstly checked accuracy of 400 annotations made by the application: 200 from personal history documents only (precision all annotations = 96.00%, precision patient annotations only = 97%), and 200 annotations over other CRIS document types (precision all annotations = 93.00%; precision patient annotations only = 66%). Of the last estimate, many false positives were for occupation annotations for 'other'.
February 2020	Natasha checked 200 'other' occupation annotations to test the accuracy of this annotation and whether it should be excluded.	Precision for 'other' annotations only reached 23.5%. The false positives for this annotation seemed to fit 3 categories: text about job-seeking (e.g. looking for work), text about working on health/personal goals (e.g. working on his anxiety) or other incorrect annotations (e.g. blood work).
March 2020	Natasha looked at recall and precision more closely. Jyoti ran the application over the personal history table in gate (with extracts accessed via Dave Chandran's personal history app). Natasha selected 200 random documents from this personal history table, annotated them according to this occupation annotation guideline (excluding 'other' annotations), and then checking to see whether the app had identified these occupations (recall) or had identified any false positives (precision). As patient occupations are only mentioned rarely in the clinical record, it was not feasible to do a recall/precision check on all other types CRIS documents, therefore personal history documents are chosen as a targeted and feasible document to check.	When looking at all occupation relation annotations, the app had a precision level of 90.04 and recall level of 85.77. When looking at patient relation only annotations, the application reached precision of 77.33 and recall of 79.37.

For peer review only - http://bmjopen.bmj.com/site/about/guidelines.xhtml

Appendix 2

Annotation Guidelines Version 1

Date: 04/08/2017

This guideline outlines the process for annotating occupation status in GATE. The term highlighted should be the word(s) in the free text which indicates the occupation of an individual, as described in the personal history of the patient. After reading the free text, annotations should be made on the word(s) which are related to an employment status or an occupation: job or profession. For all cases, each annotation will have the following features: **occupation and subject of occupation.** The exclusion criteria outline when no annotations should be made.

**Rules for annotating Occupation Status**			
Rule	Rule Description	Example	Actual Annotation
Multiple occupations	All occupations mentioned in the free text (personal history) should be annotated	Chef, 7.5-tonne truck driver	Occupation: chef, truck driver
Working role is given, without occupation mentioned	Annotate with closely related description	Daily role involves operating the machines	Occupation: production worker/machine operator
Related occupations	Annotate all occupations which are mentioned which are associated with the progress of the same job	Worked as a social worker and later became a manager	Occupation: social worker, social work manager
Place or sector is mentioned without occupation	Annotate the company or sector	XX works for the council	Occupation: council worker
Loose description of job role which cannot be titled	Annotate the reference to odd jobs which have relevance	XX does various jobs which include, tiling, plumbing…	Occupation: Tiler and Plumber

For peer review only - http://bmjopen.bmj.com/site/about/guidelines.xhtml

**Rules for annotating Student Status**			
Student (full time/part time)	Annotate term student or a description of full time/ part time study	XX is currently studying XX at university	Occupation: student
**Rules for annotating Retired Status**			
Retirement	Annotate the term retired or description of retirement	Worked until retirement	Occupation: retired
**Rules for annotating Self-Employed Status**			
Self-employed without job description	Annotate the term or description of self-employed	Patient is self-employed	Occupation: self-employed
Self-employed with job description	Annotate the term or description of self-employed and job description	Patient is a self-employed builder	Occupation: self-employed, builder
**Rules for annotating Other Occupation Status**			
Difficult to define or job/role not stated	Annotate relevant phrase	Works occasionally on weekends	Occupation: other
**Rules for annotating Unemployed Status**			
Unemployment	Annotate the term unemployed or the description of unemployment	XX has not worked for several years	Occupation: unemployed

For peer review only

Supplementary File 3: Performance evaluations for the two versions of the occupation application applied to the Clinical Record Interactive Search case register: with machine-learning (and combined rule-based approaches), and without machine-learning (rule-based approaches only)

Documents	Application Version	Precision	Recall	F1 measure
77 personal history training corpus	3.5(With Machine-Learning)	0.88	0.90	0.89
77 personal history training corpus	3.5(Without Machine-Learning)	0.87	0.81	0.84

Table 1: Evaluation of occupation applications on the training corpus, calculated on GATE software

Documents	Application version	Precision	Occupation precision	Relation precision
100 personal history	3.5(With Machine-Learning)	0.92	0.96	0.91
	3.5(Without Machine-Learning)	0.95	0.96	0.85
100 other CRIS document types	3.5(With Machine-Learning)	0.79	1	0.68
	3.5(Without Machine-Learning)	0.94	1	0.58

Table 2: Evaluation of occupation applications on the test corpus of documents where the applications had identified an occupation, calculated manually.
**Precision = true positive annotations/all annotations*
*** Occupation precision = true positive occupation titles/all occupation titles*
****Relation precision = true positive relation assignments/all relation assignments*

For peer review only

BMJ Open

Text-mining occupations from the mental health electronic health record: a natural language processing approach using records from the Clinical Record Interactive Search (CRIS) platform in south London, UK.

Journal:	*BMJ Open*
Manuscript ID	bmjopen-2020-042274.R1
Article Type:	Original research
Date Submitted by the Author:	22-Sep-2020
Complete List of Authors:	Chilman, Natasha; King's College London, Department of Psychological Medicine; Insitute of Psychiatry, Psychology & Neuroscience<br>Song, Xingyi; The University of Sheffield<br>Roberts, Angus; King's College London, Institute of Psychiatry, Psychology and Neuroscience<br>Tolani, Esther; King's College London, Institute of Psychiatry, Psychology & Neuroscience<br>Stewart, Robert; King's College London, Institute of Psychiatry; South London and Maudsley NHS Foundation Trust<br>Chui, Zoe; King's College London, Institute of Psychiatry, Psychology & Neuroscience<br>Birnie, Karen; King's College London; King's College Hospital NHS Foundation Trust<br>Harber-Aschan, Lisa; King's College London<br>Gazard, Billy; King's College London<br>Chandran, David; South London and Maudsley NHS Foundation Trust, NIHR Biomedical Research Centre<br>Sanyal, Jyoti; South London and Maudsley NHS Foundation Trust, NIHR Biomedical Research Centre<br>HATCH, STEPHANI; King's College London, Institute of Psychiatry, Psychology and Neuroscience<br>Kolliakou, Anna; King's College London, Institute of Psychiatry, Psychology & Neuroscience<br>Das-Munshi, Jayati; King's College London, Department of Psychological Medicine, Institute of Psychiatry, Psychology & Neuroscience
**Primary Subject Heading**:	Health informatics
Secondary Subject Heading:	Epidemiology, Mental health, Occupational and environmental medicine
Keywords:	MENTAL HEALTH, Health informatics < BIOTECHNOLOGY & BIOINFORMATICS, EPIDEMIOLOGY, Adult psychiatry < PSYCHIATRY

SCHOLARONE™
Manuscripts

I, the Submitting Author has the right to grant and does grant on behalf of all authors of the Work (as defined in the below author licence), an exclusive licence and/or a non-exclusive licence for contributions from authors who are: i) UK Crown employees; ii) where BMJ has agreed a CC-BY licence shall apply, and/or iii) in accordance with the terms applicable for US Federal Government officers or employees acting as part of their official duties; on a worldwide, perpetual, irrevocable, royalty-free basis to BMJ Publishing Group Ltd ("BMJ") its licensees and where the relevant Journal is co-owned by BMJ to the co-owners of the Journal, to publish the Work in this journal and any other BMJ products and to exploit all rights, as set out in our licence.

The Submitting Author accepts and understands that any supply made under these terms is made by BMJ to the Submitting Author unless you are acting as an employee on behalf of your employer or a postgraduate student of an affiliated institution which is paying any applicable article publishing charge ("APC") for Open Access articles. Where the Submitting Author wishes to make the Work available on an Open Access basis (and intends to pay the relevant APC), the terms of reuse of such Open Access shall be governed by a Creative Commons licence – details of these licences and which Creative Commons licence will apply to this Work are set out in our licence referred to above.

Other than as permitted in any relevant BMJ Author's Self Archiving Policies, I confirm this Work has not been accepted for publication elsewhere, is not being considered for publication elsewhere and does not duplicate material already published. I confirm all authors consent to publication of this Work and authorise the granting of this licence.

For peer review only

Text-mining occupations from the mental health electronic health record: a natural language processing approach using records from the Clinical Record Interactive Search (CRIS) platform in south London, UK.

Natasha Chilman[1*], Xingyi Song[3], Angus Roberts[1], Esther Tolani[1], Robert Stewart[1,2] Zoe Chui[1], Karen Birnie[1, 5], Lisa Harber-Aschan[1], Billy Gazard[1], David Chandran[2], Jyoti Sanyal[2], Stephani L Hatch[1,4], Anna Kolliakou[1, **], Jayati Das-Munshi[1,2, 4**]

***Joint senior author*

***Corresponding author contact information:**

Natasha Chilman, East Wing 3.16, Department of Psychological Medicine, Institute of Psychiatry, Psychology & Neuroscience, Kings College London, DeCrespigny Park, London, SE5 8AF. Telephone: +44 796968 8554. Email: natasha.chilman@kcl.ac.uk

Author details:

[1] Institute of Psychiatry, Psychology and Neuroscience, King's College London, London, UK. [2] South London and Maudsley NHS Foundation Trust, London, UK. [3] University of Sheffield, Sheffield, UK. [4]Economic and Social Research Council (ESRC) Centre for Society and Mental Health, King's College London, UK. [5]King's College Hospital NHS Trust, London, UK.

ORCID identifiers:

Natasha Chilman 0000-0002-9661-5098

Xingyi Song 0000-0002-4188-6974

Angus Roberts 0000-0002-4570-9801

Esther Tolani 0000-0002-7415-0859

Robert Stewart 0000-0002-4435-6397

Zoe Chui 0000-0001-6844-6779

Karen Birnie 0000-0003-4123-1676

Lisa Harber-Aschan 0000-0002-6464-485

Billy Gazard 0000-0002-7562-539

For peer review only - http://bmjopen.bmj.com/site/about/guidelines.xhtml

David Chandran 0000-0002-0123-666X

Jyoti Sanyal – N/A

Stephani Hatch 0000-0001-9103-2427

Anna Kolliakou 0000-0003-1234-4129

Jayati Das-Munshi 0000-0002-3913-6859

Word Count

4,219

Keywords

Mental Health

Health informatics

Epidemiology

Adult psychiatry

For peer review only - http://bmjopen.bmj.com/site/about/guidelines.xhtml

ABSTRACT

Objectives

We set out to develop, evaluate, and implement a novel application using natural language processing to text-mine occupations from the free-text of psychiatric clinical notes.

Design

Development and validation of a natural language processing application using General Architecture for Text Engineering (GATE) software to extract occupations from de-identified clinical records.

Setting & Participants

Electronic health records from a large secondary mental health provider in south London, accessed through the Clinical Record Interactive Search (CRIS) platform. The text-mining application was run over the free-text fields in the electronic health records of 341,720 patients (all aged ≥16).

Outcomes

Precision and recall estimates of the application performance; occupation retrieval using the application compared to structured fields; most common patient occupations; and analysis of key sociodemographic and clinical indicators for occupation recording.

Results

Using the structured fields alone, only 14% of patients had occupation recorded. By implementing the text-mining application in addition to the structured fields, occupations were identified in 57% of patients. The application performed on gold-standard human-annotated clinical text at a precision level of 0.79 and recall level of 0.77. The most common patient occupations recorded were ‘student’, and ‘unemployed’. Patients with more service contact were more likely to have an occupation recorded, as were patients of a male gender, older age, and those living in areas of lower deprivation.

Conclusion

This is the first time a natural language processing application has been used to successfully derive patient-level occupations from the free-text of electronic mental health records, performing with good levels of precision and recall, and applied at scale. This may be used to inform clinical studies relating to the broader social determinants of health using electronic health records.

For peer review only - http://bmjopen.bmj.com/site/about/guidelines.xhtml

ARTICLE SUMMARY

Strengths and Limitations

- The application was developed on a sizeable corpus of training and test data from a large routine dataset, which was applied at scale over the record, providing us with insights into the occupations of patients using secondary mental health services.
- The application was thoroughly evaluated using gold-standard and cross-checking strategies.
- The application was developed and tested in a single site electronic health record system in the UK – the application will require validation on other similar systems before use with them.
- The application does not identify the temporality of occupations; it is unclear whether the extracted occupations are currently or previously held by the patient.
- Health and social care occupations were prevented from being assigned to the patient as these could not be ascertained with confidence, therefore the application cannot yet identify where a patient holds a health/social care occupation.

For peer review only

INTRODUCTION

Occupation and mental illness are highly interrelated. There are long-standing concerns that unemployment rates are considerably higher for people with mental illness [1, 2], and work participation has been described as among the most important factors for recovery by clinicians and service users alike [3, 4]. People with mental illnesses may also undertake precarious, poorly paid work which could have further negative impacts on mental health [5]. Moreover, occupation is a fundamental individual-level indicator of socio-economic position as it is predictive of material resources and is indicative of wider class interactions [6]. Recent systematic reviews have called for large and detailed longitudinal studies to investigate predictors of occupational functioning, and to examine how and when occupation is associated with clinical outcomes in mental health cohorts, as this is currently poorly understood [7, 8].

Research using electronic health records (EHRs) allows for the large-scale collection of sociodemographic and clinical information which would otherwise be logistically challenging to collect using traditional epidemiological approaches [9]. However, EHR research has major limitations including that information relating to occupation is either not recorded routinely or is poorly captured within standard EHR systems [10]. As there are no existing methods, to our knowledge, to reliably extract occupations from the psychiatric EHR, this is a problematic barrier for desirable research where occupation is an indicator of socioeconomic status and in research examining the relationships between occupation, mental illness and recovery.

Patient information can be recorded in the structured fields of the EHR, where the clinician records categorical or numerical data. In many psychiatric EHR systems, patient information is recorded in narrative text sections of the record, known as the 'free-text' fields, for example in notes describing patient contact [11]. Information recorded in this way is harder to extract. Clinicians may only record the patient's occupation in such free-text fields and not the structured fields, making it more complicated, time consuming and labour intensive to identify the patient's occupation [10]. Natural language processing (NLP) methods have the potential to overcome this obstacle by applying algorithms to extract relevant textual information. NLP methods have previously been used successfully for text-mining from mental health EHRs, for example to identify smoking status and symptoms of severe mental illness [12-16], and other types of clinical records [17, 18]. NLP methods are also being applied in large-scale industrial and occupational research [19-21]. This paper traces the development of a novel application using NLP methods to extract patient occupations from the free-text of EHRs from a large mental health Trust in south London, UK. We then provide profile information on the most frequently extracted occupations for patients using secondary mental health services, and clinical and sociodemographic factors associated with recorded occupation data compared to missing occupation data.

For peer review only

MATERIALS AND METHODS

Setting

Data for the development of the application were obtained from the South London and Maudsley (SLaM) Biomedical Research Centre (BRC) Case Register: a repository of de-identified clinical data from the EHRs of individuals receiving care from SLaM secondary mental health services. SLaM covers a socially and ethnically diverse inner-city area of approximately 1.3 million people [22]. The register contains over 350,000 de-identified patient records which are available for research purposes through the Clinical Record Interactive Search (CRIS) platform. CRIS was developed at SLaM in 2008 and similar resources have subsequently been implemented at several other mental health Trusts in the UK. The present application was developed over the years 2017-2019 and was implemented in January 2020.

Datasets

Figure 1 describes how the CRIS-derived dataset was used for cycles of application development and evaluation, and summarises the key steps taken. Age restrictions were implemented throughout document selection: free-text documents were only extracted where the patient was aged 16 and above at time of document extraction. There were no date restrictions. Free-text documents were retrieved from several different sections in this EHR, for example sections for clinical risk assessments and separate sections for discharge summaries. Further detail on the types of documents used at each stage of application development can be found in supplementary file 1.

Developing, Evaluating and Implementing the Application

Manually annotating occupation in the free-text (Figure 1, steps 1-3)

Personal history sections of psychiatric assessments typically describe the patient's occupation, as well as education and family history. Personal history sections of documents were extracted from the free-text fields of records at the document level using an NLP application (precision=0.78, recall=0.88) developed by DC (N=67,383). Typically these extracts were derived from documents of the 'attachments' type, which is a word-processed document such as a letter to or from the patient's primary care physician; and 'events', which are short pieces of text used to record some detail of a clinical encounter.

Occupations were identified in personal history documents by an interdisciplinary team of trained researchers, including clinicians, bioinformaticians and mental health researchers. In common with the NLP community, we refer to this task of marking mentions of occupation text as annotation. A set of occupation annotation guidelines were developed through an iterative process of manual annotation practice, team discussions and agreed annotation rulemaking (supplementary file 2). These guidelines

For peer review only - http://bmjopen.bmj.com/site/about/guidelines.xhtml

specified when and how an occupation should be identified, annotated and extracted from the text. An occupation annotation was defined as having two parts. Firstly, the *occupation* itself was annotated. This could be an occupation title, for example a 'builder'; or an occupation description, for example 'construction'. Secondly, the occupation *relation* was specified: who the occupation belongs to, for example the patient or their family member. Temporality, including when or how long a patient has held an occupation, was not annotated as the text often did not state this consistently. In total, 600 personal history documents were manually annotated to practice annotating occupation from text and develop the annotation guidelines (ET, AK, SM, KB, ZC, AR). Once the guidelines were developed, a set of 1000 personal history documents were manually annotated on the General Architecture for Text Engineering (GATE) platform [23] using the guidelines to a gold-standard, where 200 were double annotated to evaluate inter-annotator reliability.

Application development (Figure 1, step 4)

Out of the 1000 gold-standard annotated personal history documents, 334 documents were reserved for application development. The application was developed by XS on the GATE platform [23], a widely used NLP framework with over 40 thousand downloads per version and a history of use in the UK national health service, amongst other sectors [17]. The application was trained on 257 of the gold-standard annotated documents. To check the performance of the application throughout development, precision and recall metrics were estimated using a customized performance tool developed by XS on GATE on a validation set of 77 gold-standard annotated documents, with a total of 405 occupation annotations. Precision was the proportion of occupations correctly annotated, to all occupations annotated (whether correct or incorrect). Recall was the proportion of occupations correctly annotated, to all occupations that could have been correctly annotated. The application outputs were manually checked by the Clinical Informatics Interface and Network Lead at the NIHR BRC (AK). Any problems identified were addressed in each version of the application. An iterative process of application development, training, evaluation of performance using GATE and manual checks was repeated 10 times, at which point the application reached a good level of performance on the validation set.

Machine-learning approach testing (Figure 1, steps 5-6)

Two early versions of the application were developed for testing over unannotated documents in the CRIS case register: one version used combined machine-learning and rule-based approaches, and the second version used rule-based approaches only. This was due to a concern that the application had therein been developed on limited training data, and the trained model may not generalise well on the free-text other than personal history documents, which could lead to a loss in precision when implemented over the EHR. Specifically, the machine-learning approaches involved a trained conditional random field classifier to identify occupation mentions in the text, and a support-vector machine-based classifier to identify the occupation relation. Figure 2 illustrates how the machine-

For peer review only - http://bmjopen.bmj.com/site/about/guidelines.xhtml

learning and rule-based approaches were used in combination; this is described in further technical detail in supplementary file 3.

Two researchers (NC, AK) manually calculated precision performance for both versions of the application on 100 personal history documents (in domain testing data) and 100 other free-text document types (out domain test data) which had at least one occupation extraction and were previously unseen by the application in development. Whilst both application versions performed well when text-mining occupations from these test sets (precision ≥0.79, further detail in supplementary file 3), the application with machine-learning approaches performed at the highest level of precision when assigning the occupation relation - i.e. who the occupation was held by. The research team concluded from this testing phase that the application with combined machine-learning and rule-based approaches was most appropriate, as this pipeline performed best at assigning the occupation relation.

The healthcare occupation filter (Figure 1, step 7)

The evaluation of the application performance over CRIS documents revealed that the most common false positives were extractions where the healthcare professional involved in the patient's care was incorrectly annotated as the patient's occupation (96% of annotations manually checked were health/social care occupations). To deal with this issue, health and social care occupations were added to a filter. The application then implemented a rules-based step where the filtered healthcare occupations were prevented from being annotated as belonging to the patient. Occupations added to this filter included variations on terms for psychiatrists and doctors, therapists, nurses, and social workers, following the checking of 2,390 documents to confirm that these were common false positives.

Application implementation and testing (Figure 1, steps 8-10)

The final version of the text-mining application with the healthcare filter applied was run over 10 free-text fields, including those where personal history sections were found, in the records of all patients on the CRIS case register aged 16 and above. The fields included sections of the record such as discharge summaries, attachments, events and risk assessments (more detail in supplementary file 1). The application was evaluated on a total of 866 documents: 666 gold-standard annotated personal history documents (test corpus 1), and 200 previously unannotated random personal history documents from the CRIS dataset at the time of the application run (test corpus 2). Test corpus 1 was evaluated on GATE, and test corpus 2 was manually checked for occupations and then cross-referenced with the application output. The performance metrics considered the precision and recall level for the annotations made by the application, where both the occupation annotation and the relation classification needed to be accurate to be considered a 'true positive'. It was not feasible in this study to randomly select non-personal-history documents for evaluation as patient occupations were rarely mentioned in the record compared to other information (e.g. medication). As the application extracted

For peer review only - http://bmjopen.bmj.com/site/about/guidelines.xhtml

an annotation entitled ‘other’, 200 of these annotations were manually checked for precision to further investigate these instances where the application was unable to assign an occupation title.

The EHR in the present study contains a structured field to record occupation: the ‘Employment-ID’. This was explored on the CRIS platform using SQL queries. The proportion of completed ‘Employment-IDs’ from the records of all patients over the age of 16 in January 2020 was extracted. The text-mining application was simultaneously run over clinical records through CRIS, and the extracted patient occupations were converted into an SQL table. Sociodemographic, clinical and service contact data was also extracted from the structured fields of records using SQL queries. Data was exported to and analysed in STATA-15 to examine predictors of occupational data extraction using logistic regression models. This included the patient’s age at time of occupation extraction, gender, marital status, ethnicity, index of multiple deprivation (IMD) score and primary diagnosis. Indicators of service contact included number of events in the record, number of face-to-face events in the record, number of spaces in the free-text fields of the record (as a proxy for word count), number of active days under SLaM services, and number of inpatient bed days. These variables were transformed into categories, for example IMD scores were categorised into quartiles of local neighbourhood deprivation. Where data was missing for the extracted variables, this was coded as a ‘Not Known’ category for each variable.

Logistic regression models examined crude associations between the sociodemographic, clinical, and service contact variables (predictors) and the recording of at least one patient-occupation (outcome) from either the structured or free-text fields. The null hypothesis was that none of the predictors would be associated with likelihood of occupation recording. Firstly, models were adjusted for amount of contact the patient had with services. Fully adjusted models accounted for all other sociodemographic and clinical variables. Across all models, likelihood ratio tests were conducted to test the overall association between the variable and occupation recording. The aim of this analysis was to ascertain the characteristics of patients who had occupation recorded in their health record.

Patient and Public Involvement

This study proposal was reviewed and approved by the patient-led CRIS oversight committee prior to the commencement of the project. No other consultations were made with patients or the public during the process of the study.

For peer review only

RESULTS

Annotating Occupation

When double-annotating 200 personal history documents, two annotators reached a Cohen's kappa agreement of 0.77 for occupation title annotations and 0.72 for occupation relation annotations. Disagreements between annotators included instances where sentences posed unclear or vague references to occupation: for example, in the sentence, "she did several things, such as cleaning, cooking", it was not clear whether these were domestic tasks or occupation-descriptions, demonstrating the complexity of annotating occupation from text. Nonetheless, the Cohen's kappa agreement suggested that occupation could be annotated reasonably consistently across annotators using the annotation guidelines.

Application Development

The application reached a precision level of 0.88 and a recall level of 0.90 on the validation set of documents (N=77). The developed application process with combined rule-based and machine learning approaches is described in Figure 2.

Application Performance

When applied to the gold-standard annotated personal history documents (test corpus 1) on GATE, the application performed at a precision level of 0.79 and a recall level of 0.77. Two-hundred personal history documents were manually checked for occupations and then cross-referenced with the application output (test corpus 2): when considering patient-occupations only, the application reached a precision level of 0.77 and recall level of 0.79. An extraction of 'other' as an occupational category was excluded from subsequent analysis, as the check of 200 annotations showed that this annotation only reached a precision level of 0.23 and often referenced job-seeking or non-work behaviours, for example 'working on his anxiety'.

Application Implementation

Figure 3 shows the study population selection process for the implementation of the application over the CRIS case register, leading to an overall sample size of 341,720 patients.

Descriptives

Demographics of the study population at time of occupation extraction is described in Table 1, as well as patient diagnostic categories and two indicators of the amount of service contact the patient has had: the number of 'events' entries added to the EHR, and number of inpatient bed days. The three other extracted indicators for service contact (number of 'face-to-face events', total active days under SLaM mental health services, and number of spaces in the text in the record) were excluded from analysis due to collinearity with the 'events' variable.

Occupation Extractions

For peer review only - http://bmjopen.bmj.com/site/about/guidelines.xhtml

The structured field for employment was populated for 46,705 (13.7%) patients. Prior to the implementation of the healthcare filter, 81.5% patients had at least one patient-occupation extraction. When using the final version of application to extract occupations from the free-text fields with the healthcare filter applied, this recalled at least one patient-related occupation for 184,521 patients (54.0%). By combining structured field and extracted occupations, patient-related occupations were retrieved for 193,616 patients (56.7%) over the dataset.

The structured field for occupation included 13 categories for occupational status, for example 'unemployed' or 'paid employment'. In contrast, the text-mining application retrieved 72,955 different patient-related occupation types. In total, there were 3,957,959 patient-related occupation extractions. Multiple occupation types were often extracted per patient (median=4, inter-quartile-range=6).

The top 5 extracted occupations across the total sample of 341,720 patients were: student (n=98,719, 28.9%), unemployed (n=97,809, 28.6%), carer (n=61,893, 18.1%), self-employed (n=36,506, 10.8%) and retired (n=33,518, 9.8%). The less frequent extractions tended to be more specific occupation types, for example, 'retail worker', and 'banker'. The application also extracted 'undocumented' ways of making money, including 'drug dealer' and 'sex worker'.

Associations with Occupation Recording

Patients were split into two binary categories: those who had an occupation recorded either in the structured field or free-text (n=193,616, 56.7%), and patients who did not have occupation recorded, i.e. missing occupational data (n=148,104, 43.4%). Logistic regressions were used to examine sociodemographic, clinical, and service contact associations with recorded occupations (Table 2).

Across all models, all predictors were strongly associated with a recording of occupation even after fully adjusting for all other variables (likelihood ratio tests $p<.0001$). When key sociodemographic data was missing from the record, the odds of occupational data being recorded decreased: for example, where the marital status of the patient was 'Not Known', the fully-adjusted odds ratio for a recording of an occupation was 0.49 (95% CI 0.47-0.50) compared to patients who were recorded as married/in a civil partnership/cohabiting. Female patients were significantly less likely to have an occupation extracted compared to male patients, and older patients were most likely to have occupational data recorded compared to the youngest patients. Compared to patients of White British ethnicity, patients of Irish, Black Caribbean, or Black African ethnicity were more likely to have an occupation recorded; whilst Indian, Pakistani, Chinese, Mixed Race or recorded as being from 'other' Asian or ethnic groups were less likely to have occupation recorded. The odds of having occupation recorded were significantly lower for patients who were living in the most deprived local areas compared to the most affluent areas. Generally, patients with a primary diagnosis of an affective disorder had a higher odds of an occupation extraction than patients with other diagnoses, including organic disorders. In the crude logistic regression models, patients diagnosed with schizophrenia, schizotypal or delusional disorders were more likely to have occupation

For peer review only - http://bmjopen.bmj.com/site/about/guidelines.xhtml

extracted (OR 1.61, P5% CI 1.54-1.68). However, once adjusting for amount of contact with services, these patients were significantly less likely to have occupation extracted compared to patients with affective disorders (adjusted OR 0.87, 95% CI 0.83-0.91).

For peer review only

DISCUSSION

Annotating and extracting occupation from the free-text in clinical records is a challenging task. We have developed innovative methodology to text-mine patient occupations with a good degree of confidence from a mental health EHR, and applied this at scale over a large EHR in south London. An important finding was that we could retrieve over double the number of patient occupations using text-mining methodology than when using pre-existing structured fields alone. We could also access a much wider diversity of occupation types: this further detail on occupations held by patients opens up the possibility for the translation of occupations onto social class schema, which would not have been possible with the limited structured field categories. The most prevalent patient-occupations were 'student' and 'unemployed'. There were differences between patients who had occupation recorded and patients where occupation data remained missing: patients with occupations recorded were more likely to be of an older age, male, divorced/separated, living in areas of lower deprivation, and had more contact with mental health services. Across ethnic minority groups, there were mixed findings relating to the recording of occupation. Compared to White British patients, Irish, Black Caribbean and Black African patients were slightly more likely to have a recording of occupation, whereas all other ethnic minority groups were less likely to have a recording. Although it is possible that some of the demographic associations with the recording of occupation in the case notes were impacted upon by residual confounding in adjusted models, these findings may also indicate disparities relating to how occupations are assessed and recorded in the clinical record and should be explored in future work, particularly given the strong correlation of employment with recovery, within the context of mental disorders.

This study broadly supports the work of other studies which indicate that clinicians mostly describe occupation in the free-text of EHR systems, when these are available, rather than structured fields [10]. This study is the first of its kind to text-mine patient occupations from a mental healthcare EHR. There have been several previous efforts to extract patient occupations from other healthcare free-text notes. Occupations have been text-mined from general medical clinical text; however, in these studies the algorithms reached low levels of performance, largely due to a lack of training data [24, 25]. Dehghan and colleagues' text-mined occupation from the clinical records of cancer patients in the UK, reaching similar precision and recall levels to the present study [26]. However, none of these applications distinguished between text-mining occupations belonging to the patient and other relations, had the scope of applying and testing the text-mining methodology at scale across the EHR, or examined associations with extracted versus missing occupational data. The present application therefore represents significant progress in our ability to text-mine patient occupations from the EHR and furthers our understanding of what this may mean in practice.

For peer review only - http://bmjopen.bmj.com/site/about/guidelines.xhtml

We found that text-mining greatly increased our retrieval of patient-occupations in this psychiatry EHR database. Psychiatric notes may be more detailed than other types of healthcare text (for example, in general medicine) when describing the patient's occupation, as this often forms part of psychiatric history taking and assessment. We found that a sizeable proportion of patients over CRIS have at some point been a student or unemployed. A separate NLP application being developed using CRIS data (by author JS) will be able to interrogate this student group further by extracting the patient's level of educational attainment, which will complement the present application. There is also scope to explore older groups of patients who are students but are also working using this methodology. Our finding that unemployment was a dominant occupational category is consistent with previous research, in that unemployment levels are elevated particularly for those with severe mental illnesses compared to the general population [1, 2]. It may also be the case that patients in this group are formally unemployed but are working in more informal, undocumented ways to make money. This application identified some informal occupations, which is an interesting avenue for further research.

One limitation of our approach is that we could not distinguish the temporality of occupations – whether they were currently or previously held by the patient. Whilst developing the annotation guidelines, we found that the text was unlikely to be sufficient to assess temporality, as it was often not explicitly stated when the patient started or left an occupation, or how long they have held a position for. Multiple occupations were often extracted for a single patient, adding to the complexity. Whilst there is work ongoing to use NLP to detect temporality in psychiatric healthcare text [27], this remains a challenge and is a potential avenue for further work that is beyond the scope of this paper. As this application was developed at a single site in the UK, the generalisability of the application may be reduced, firstly to English language and secondly to this catchment area. As it was not possible to assign health and social care occupations to patients with reasonable confidence, we will also be missing patients who hold these occupations; however, we are planning further work to develop this aspect of the application further. Notwithstanding these limitations, this application was developed through an extensive process of training and testing using a large corpus leading to the application of text-mining algorithms for occupation at scale. This methodology is already revealing the kinds of occupations held by patients using secondary mental health services.

The development of this methodology has numerous implications. Firstly, this application will be valuable in allowing researchers to examine relationships between occupation and health in large psychiatric case registers. For example, work is currently underway using this application to investigate predictors of unemployment in a cohort of patients with severe mental illness [28]. As CRIS-like systems are in use over several sites in the UK, there is the scope to test and implement this application in other mental healthcare providers using similar EHR platforms. This application could also have potential practical implications including identifying unemployed patients to target interventions such as Individual Placement and Support (IPS) and retrieving occupational distributions for audits and

For peer review only - http://bmjopen.bmj.com/site/about/guidelines.xhtml

organisational monitoring in NHS mental health Trusts. Lastly, this application may have implications beyond mental health research and text, notably in industrial research, although this requires further testing.

There is room for further progress in this application as the NLP field further develops, including identifying the temporality of occupations and improving relation classification for health and social care occupations. We plan to develop methodology to ascertain the occupational social class of patients, using the large diversity of occupations extracted, to further inform health inequalities research specific to mental health. Future studies implementing this application in other CRIS systems may be able to investigate the transferability of the application to other NHS sites in the UK that serve different patient populations. Overall, we hope that this approach will prove useful in forwarding our understanding of the interactions between occupation and health in those with mental illness.

For peer review only

ORIGINAL PROTOCOL

N/A

FUNDING STATEMENT

This work was supported by the Clinical Record Interactive Search system which is funded by the National Institute for Health Research (NIHR) Biomedical Research Centre at South London and Maudsley National Health Service (NHS) Foundation Trust and King's College London and a joint infrastructure grant from Guy's and St Thomas' Charity and the Maudsley Charity. Specific application development was supported by the Health Foundation, in a grant held by JD.

JD is funded by the Health Foundation working together with the Academy of Medical Sciences, for a Clinician Scientist Fellowship and by the ESRC in relation to the SEP-MD study (ES/S002715/1) and part supported by the ESRC Centre for Society and Mental Health at King's College London (ESRC Reference: ES/S012567/1).

RS is part-funded by: i) the National Institute for Health Research (NIHR) Biomedical Research Centre at the South London and Maudsley NHS Foundation Trust and King's College London; ii) a Medical Research Council (MRC) Mental Health Data Pathfinder Award to King's College London; iii) an NIHR Senior Investigator Award; iv) the National Institute for Health Research (NIHR) Applied Research Collaboration South London (NIHR ARC South London) at King's College Hospital NHS Foundation Trust.

SH is part-funded by the National Institute for Health Research (NIHR) Biomedical Research Centre at the South London and Maudsley NHS Foundation Trust and King's College London; ESRC Centre for Society and Mental Health at King's College London (ESRC Reference: ES/S012567/1); and the Wellcome Trust (203380Z16Z).

The funders had no role in the study design, data collection and analysis, decision to publish, or preparation of the manuscript. The views expressed are those of the author(s) and not necessarily those of the NHS, the NIHR, the MRC, the Department of Health, the ESRC or King's College London.

COMPETING INTERESTS STATEMENT

All authors have confirmed that they have no competing interests to declare.

CHECKLIST/FLOW DIAGRAM FOR REPORTING STATEMENT

For peer review only - http://bmjopen.bmj.com/site/about/guidelines.xhtml

The RECORD Statement checklist is attached to this submission.

ETHICS

The SLaM Case Register has been approved as a source of de-identified data for secondary analyses (Oxford Research Ethics Committee C, reference 18/SC/0372).

DATA SHARING STATEMENT

We are unable to place test data in the public domain because these comprise patient information, but document IDs used in application development and testing have been archived and researchers may apply for approval to access these or other CRIS data. This application is also being put into production for researchers to use in the Biomedical Research Centre. More information can be found at http://brc.slam.nhs.uk/about/core-facilities/cris.

AUTHOR CONTRIBUTIONS

The study was conceived by JD, AK, RS, AR, SH, BG and LHA. Personal history sections of documents were extracted using an application developed by DC. Manual annotations to develop the annotation guidelines and produce the test and training data were conducted by AK, AR, ET, ZC and KB, and SM (acknowledgements). The application was developed by XS, with feedback from AK, NC, JD, RS, AR, and SH. The application was implemented over the EHR by DC and JS. The application was evaluated by AK and NC. The missing data analysis was conducted by NC and JD. The paper draft was led by NC, JD and AK; and was critically reviewed and edited by all authors (AK, XS, AR, ET, RS, ZC, KB, DC, JS, BG, LHA, SH).

ACKNOLWEDGEMENTS

We appreciated the technical support from informatics personnel in the NIHR Maudsley Biomedical Research Centre and the University of Sheffield. We would also like to thank Shirlee MacCrimmon for her assistance with annotations during the annotation guideline development process.

REFERENCES

For peer review only - http://bmjopen.bmj.com/site/about/guidelines.xhtml

REFERENCES

1. Luciano, A. and E. Meara, *Employment status of people with mental illness: national survey data from 2009 and 2010.* Psychiatric Services, 2014. **65**(10): p. 1201-1209.
2. Marwaha, S., et al., *Rates and correlates of employment in people with schizophrenia in the UK, France and Germany.* The British Journal of Psychiatry, 2007. **191**(1): p. 30-37.
3. Dunn, E.C., N.J. Wewiorski, and E.S. Rogers, *The meaning and importance of employment to people in recovery from serious mental illness: results of a qualitative study.* Psychiatric rehabilitation journal, 2008. **32**(1): p. 59.
4. Marwaha, S. and S. Johnson, *Schizophrenia and employment.* Social psychiatry and psychiatric epidemiology, 2004. **39**(5): p. 337-349.
5. Moscone, F., E. Tosetti, and G. Vittadini, *The impact of precarious employment on mental health: The case of Italy.* Social Science & Medicine, 2016. **158**: p. 86-95.
6. Connelly, R., V. Gayle, and P.S. Lambert, *A review of occupation-based social classifications for social survey research.* Methodological Innovations, 2016. **9**: p. 2059799116638003.
7. Luciano, A., G.R. Bond, and R.E. Drake, *Does employment alter the course and outcome of schizophrenia and other severe mental illnesses? A systematic review of longitudinal research.* Schizophrenia research, 2014. **159**(2-3): p. 312-321.
8. Gilbert, E. and S. Marwaha, *Predictors of employment in bipolar disorder: a systematic review.* Journal of Affective Disorders, 2013. **145**(2): p. 156-164.
9. Schofield, P. and J. Das-Munshi, *Big data: what it can and cannot achieve.* BJPsych Advances, 2018. **24**(4): p. 237-244.
10. Aldekhyyel, R., et al. *Content and quality of free-text occupation documentation in the electronic health record.* in *AMIA Annual Symposium Proceedings*. 2016. American Medical Informatics Association.
11. Lovis, C., R.H. Baud, and P. Planche, *Power of expression in the electronic patient record: structured data or narrative text?* International Journal of Medical Informatics, 2000. **58**: p. 101-110.
12. Wu, C.-Y., et al., *Evaluation of smoking status identification using electronic health records and open-text information in a large mental health case register.* PloS one, 2013. **8**(9): p. e74262.
13. Jackson, R.G., et al., *Natural language processing to extract symptoms of severe mental illness from clinical text: the Clinical Record Interactive Search Comprehensive Data Extraction (CRIS-CODE) project.* BMJ open, 2017. **7**(1): p. e012012.
14. Iqbal, E., et al., *Identification of adverse drug events from free text electronic patient records and information in a large mental health case register.* PloS one, 2015. **10**(8): p. e0134208.
15. Chandran, D., et al., *Use of Natural Language Processing to identify Obsessive Compulsive Symptoms in patients with schizophrenia, schizoaffective disorder or bipolar disorder.* Scientific reports, 2019. **9**(1): p. 1-7.
16. Fernandes, A.C., et al., *Identifying suicide ideation and suicidal attempts in a psychiatric clinical research database using natural language processing.* Scientific reports, 2018. **8**(1): p. 1-10.
17. Kreimeyer, K., Foster, M., Pandey, A., Arya, N., Halford, G., Jones, S. F., ... & Botsis, T. (2017). Natural language processing systems for capturing and standardizing unstructured clinical information: a systematic review. *Journal of biomedical informatics*, *73*, 14-29.
18. Koleck, T. A., Dreisbach, C., Bourne, P. E., & Bakken, S. (2019). Natural language processing of symptoms documented in free-text narratives of electronic health records: a systematic review. *Journal of the American Medical Informatics Association*, *26*(4), 364-379.
19. Peckham, T. K., Baker, M. G., Camp, J. E., Kaufman, J. D., & Seixas, N. S. (2017). Creating a future for occupational health. *Annals of Work Exposures and Health*, *61*(1), 3-15.
20. Djumalieva, J., Lima, A., & Sleeman, C. (2018). *Classifying occupations according to their skill requirements in job advertisements* (No. ESCoE DP-2018-04). Economic Statistics Centre of Excellence (ESCoE).

For peer review only - http://bmjopen.bmj.com/site/about/guidelines.xhtml

21. Tixier, A. J. P., Hallowell, M. R., Rajagopalan, B., & Bowman, D. (2016). Automated content analysis for construction safety: A natural language processing system to extract precursors and outcomes from unstructured injury reports. *Automation in Construction*, *62*, 45-56.

22. Perera, G., et al., *Cohort profile of the South London and Maudsley NHS Foundation Trust Biomedical Research Centre (SLaM BRC) case register: current status and recent enhancement of an electronic mental health record-derived data resource.* BMJ open, 2016. **6**(3): p. e008721.

23. Cunningham, H., et al., *Getting more out of biomedical documents with GATE's full lifecycle open source text analytics.* PLoS computational biology, 2013. **9**(2): p. e1002854.

24. Hollister, B.M., et al. *Development and performance of text-mining algorithms to extract socioeconomic status from de-identified electronic health records*. in *PACIFIC SYMPOSIUM ON BIOCOMPUTING 2017*. 2017. World Scientific.

25. Yang, H. and J.M. Garibaldi, *Automatic detection of protected health information from clinic narratives.* Journal of biomedical informatics, 2015. **58**: p. S30-S38.

26. Dehghan, A., et al. *Identification of occupation mentions in clinical narratives*. in *International Conference on Applications of Natural Language to Information Systems*. 2016. Springer.

27. Viani, N., et al. *Time Expressions in Mental Health Records for Symptom Onset Extraction*. in *Proceedings of the Ninth International Workshop on Health Text Mining and Information Analysis*. 2018.

28. Chilman, N.G.M., & Das-Munshi, J., *Sociodemographic predictors of unemployment in patients with severe mental illness: an electronic health record cohort study.* Retrieved from osf.io/rx7zs, 2020

For peer review only

For peer review only - http://bmjopen.bmj.com/site/about/guidelines.xhtml

Table 1 Sociodemographic and clinical features of the Clinical Record Interactive Search (CRIS) case register.*

	**No. patients, % (Total N=341,720)**
**AGE**	
16-29	84,181 (24.63%)
30-49	123,216 (36.06%)
50-69	79,880 (23.38%)
70-89	43,852 (12.83%)
90+	10,591 (3.1%)
**GENDER**	
Male	166,480 (48.72%)
Female	175,007 (51.21%)
Other/Not Known	233 (0.07%)
**ETHNICITY**	
White British	136,289 (39.88%)
Irish	5,182 (1.70%)
Black Caribbean	34,229 (10.02%)
Black African	15,654 (4.58%)
Indian	4,345 (1.27%)
Pakistani	1,852 (0.54%)
Bangladeshi	1,088 (0.32%)
Chinese	1,124 (0.33%)
Other Asian	5,500 (1.61%)
Other Ethnic Group	19,650 (5.75%)
Other White	22,076 (6.46%)
Mixed	1,879 (0.55%)
Not Known	92,222 (26.99%)
**MARITAL STATUS**	
Married/civil partnership/cohabiting	46,617 (13.64%)
Divorced/separated/civil partnership dissolved	17,309 (5.07%)
Widowed	15,758 (4.61%)
Single	141,111 (41.29%)
Not Known	120,925 (35.39%)
**LOCAL QUARTILES OF NEIGHBOURHOOD DEPRIVATION**	
Least deprived	79,537 (23.28%)
3rd Quartile	80,049 (23.43%)
2nd Quartile	79,767 (23.34%)
Most deprived	79,829 (23.36%)
Address Not Known	22,538 (6.60%)
**PRIMARY DIAGNOSIS**	
F30-F39: mood (affective) disorders	37,796 (11.06%)
F00-F09: organic, including symptomatic, mental disorders	29,801 (8.72%)
F10-F19: mental and behavioural disorders due to psychoactive substance misuse	27,870 (8.16%)

For peer review only - http://bmjopen.bmj.com/site/about/guidelines.xhtml

F20-F29: schizophrenia, schizotypal and delusional disorders	18,253 (5.34%)
F40-F49: neurotic, stress-related and somatoform disorders	31,962 (9.35%)
F50-F59: behavioural syndromes associated with physiological disturbances and physical factors	9,166 (2.68%)
F60-F69: disorders of adult personality and behaviour	6,605 (1.93%)
F70-F79: mental retardation	2,732 (0.80%)
F80-F89: disorders of psychological development	5,874 (1.72%)
F90-F98: behavioural and emotional disorders with onset usually occurring in childhood and adolescence	12,028 (3.52%)
Other diagnosis	83,847 (24.54%)
Not Known	75,786 (22.18%)
**QUARTILES OF 'EVENTS' ENTERED INTO THE HEALTH RECORD**	
No Events	50,673 (14.83%)
Least Events (1-3)	86,818 (25.41%)
2nd Quartile (4-10)	62,804 (18.38%)
3rd Quartile (11-40)	68,774 (20.13%)
Most Events (41+)	72.651 (21.26%)
**INPATIENT BED DAYS**	
No inpatient admissions	311,099 (91.04%)
Low (1-2 days)	1,937 (0.50%)
Moderate (3-31 days)	10,587 (3,10%)
High (32+ days)	18,337 (5.37%)
**At the time of occupation application run (29.01.2020).*	

For peer review only - http://bmjopen.bmj.com/site/about/guidelines.xhtml

*Table 2 Results from crude and multivariable logistic regression analyses examining predictors of occupation recording from the Clinical Record Interactive Search (CRIS) case register. **

	**N (%) with at least one occupation retrieved by structured field/text-mining extractions**	**OR (95% CI)**	**aOR[1] (95% CI)**	**aOR[2] (95% CI)**
**AGE**				
16-29	41,653 (49.48)	Reference	Reference	Reference
30-49	68,422 (55.53%)	**1.27 (1.25-1.30)**	**1.56 (1.53-1.59)**	**1.72 (1.68-1.75)**
50-69	49,289 (61.70%)	**1.65 (1.61-1.68)**	**1.98 (1.93-2.02)**	**2.19 (2.14-2.25)**
70-89	27,175 (61.97%)	**1.66 (1.63-1.70)**	**1.71 (1.67-1.76)**	**1.60 (1.54-1.65)**
90+	7,077 (66.82%)	**2.06 (1.97-2.15)**	**2.14 (2.04-2.24)**	**2.00 (1.89-2.11)**
**GENDER**				
Male	96,141 (57.75%)	Reference	Reference	Reference
Female	97,443 (55.68%)	**0.92 (0.91-0.93)**	**0.88 (0.87-0.90)**	**0.87 (0.85-0.88)**
Other/Not Known	32 (13.73%)	**0.12 (0.08-0.17)**	**0.10 (0.07-0.15)**	**0.16 (0.10-0.24)**
**ETHNICITY**				
White British	91,575 (67.19%)	Reference	Reference	Reference
Irish	4,303 (74.04%)	**1.39 (1.31-1.48)**	**1.24 (1.17-1.33)**	**1.23 (1.15-1.31)**
Black Caribbean	24,753 (72.32%)	**1.28 (1.24-1.31)**	0.99 (0.96-1.02)	**1.06 (1.03-1.09)**
Black African	11,341 (72.45%)	**1.28 (1.24-1.33)**	**1.07 (1.03-1.11)**	**1.12 (1.07-1.17)**
Indian	2,876 (66.19%)	0.96 (0.90-1.02)	**0.91 (0.85-0.97)**	**0.91 (0.85-0.98)**
Pakistani	1,185 (63.98%)	**0.87 (0.79-0.95)**	**0.81 (0.73-0.90)**	**0.82 (0.74-0.91)**
Bangladeshi	719 (66.08%)	0.95 (0.84-1.08)	0.90 (0.78-1.03)	0.94 (0.82-1.08)
Chinese	690 (61.39%)	**0.78 (0.69-0.88)**	**0.73 (0.65-0.84)**	**0.81 (0.71-0.92)**
Other Asian	3,543 (64.42%)	**0.88 (0.84-0.94)**	**0.82 (0.78-0.87)**	**0.85 (0.80-0.91)**
Other ethnic Group	11,768 (59.89%)	**0.73 (0.71-0.75)**	**0.77 (0.75-0.80)**	**0.75 (0.72-0.77)**
Other White	14,610 (66.18%)	**0.96 (0.93-0.98)**	**0.94 (0.91-0.97)**	0.97 (0.94-1.00)
Mixed Race	1,197 (63.70%)	**0.86 (0.78-0.94)**	**0.68 (0.61-0.75)**	**0.78 (0.70-0.87)**
Not Known	25,056 (27.17%)	**0.18 (0.18-0.19)**	**0.31 (0.31-0.32)**	**0.50 (0.49-0.51)**

**MARITAL STATUS**				
Married/Civil Partnership/Cohabiting	31.037 (66.58%)	Reference	Reference	Reference
Divorced/Separated/Civil Partnership Dissolved	13,346 (77.10%)	**1.69 (1.62-1.76)**	**1.47 (1.40-1.53)**	**1.41 (1.35-1.47)**
Widowed	11,309 (71.77%)	**1.28 (1.23-1.33)**	1.05 (1.00-1.09)	**1.05 (1.01-1.10)**
Single	98,841 (70.04%)	**1.17 (1.15-1.20)**	1.02 (1.00-1.05)	**1.24 (1.21-1.27)**
Not Known	39,083 (32.32%)	**0.24 (0.23-0.25)**	**0.33 (0.32-0.33)**	**0.49 (0.47-0.50)**
**LOCAL QUARTILES OF NEIGHBOURHOOD DEPRIVATION**				
Least Deprived	48,155 (60.54%)		Reference	Reference
3rd Quartile	47,583 (59.44%)	**0.96 (0.94-0.97)**	**0.97 (0.95-0.99)**	**0.96 (0.94-0.99)**
2nd Quartile	45,842 (57.47%)	**0.88 (0.86-0.90)**	**0.94 (0.91-0.96)**	**0.93 (0.91-0.95)**
Most Deprived	41,800 (52.36%)	**0.72 (0.70-0.73)**	**0.89 (0.87-0.91)**	**0.88 (0.86-0.90)**
Address Not Known	10,236 (45.42%)	**0.54 (0.53-0.56)**	**0.70 (0.67-0.72)**	**0.77 (0.74-0.80)**
**DIAGNOSIS**				
F30-F39: mood (affective) disorders	27,057 (71.59%)	Reference	Reference	Reference
F00-F09: organic, including symptomatic, mental disorders	20,269 (68.01%)	**0.84 (0.82-0.87)**	**0.91 (0.88-0.94)**	**0.71 (0.68-0.74)**
F10-F19: mental and behavioural disorders due to psychoactive substance misuse	18,150 (65.12%)	**0.74 (0.72-0.77)**	**0.71 (0.68-0.73)**	**0.47 (0.45-0.49)**
F20-F29: schizophrenia, schizotypal and delusional disorders	14,645 (80.23%)	**1.61 (1.54-1.68)**	**0.87 (0.83-0.91)**	**0.78 (0.74-0.82)**
F40-F49: neurotic, stress-related and somatoform disorders	19,920 (62.32%)	**0.66 (0.64-0.68)**	**0.75 (0.72-0.77)**	**0.76 (0.73-0.79)**
F50-F59: behavioural syndromes associated with physiological disturbances and physical factors	5,287 (57.68%)	**0.54 (0.52-0.57)**	**0.65 (0.62-0.68)**	**0.68 (0.64-0.72)**

For peer review only - http://bmjopen.bmj.com/site/about/guidelines.xhtml

F60-F69: disorders of adult personality and behaviour	4,739 (71.75%)	1.01 (0.95-1.07)	**0.68 (0.64-0.73)**	**0.77 (0.72-0.82)**
F70-F79: mental retardation	2,277 (83.35%)	**1.99 (1.79-2.20)**	**1.81 (1.63-2.03)**	**1.69 (1.51-1.90)**
F80-F89: disorders of psychological development	4,377 (74.78%)	**1.16 (1.09-1.24)**	**1.22 (1.14-1.30)**	**1.78 (1.66-1.92)**
F90-F98: behavioural and emotional disorders with onset usually occurring in childhood and adolescence	8,754 (72.78%)	**1.06 (1.01-1.11)**	**1.25 (1.19-1.32)**	**1.84 (1.74-1.93)**
Other diagnosis	43,787 (52.22%)	**0.43 (0.42-0.45)**	**(0.68-0.72)**	**0.76 (0.73-0.78)**
Not Known	24,354 (32.14%)	**0.19 (0.18-0.19)**	**0.44 (0.43-0.45)**	**0.66 (0.64-0.68)**
**QUARTILES OF 'EVENTS' ENTERED INTO THE HEALTH RECORD**				
No Events	12,012 (23.70%)	Reference	Reference	Reference
Least Events	35,009 (40.32%)	**2.17 (2.12-2.23)**	**2.18 (2.13-2.23)**	**1.75 (1.70-1.79)**
2nd Quartile	34,368 (54.72%)	**3.89 (3.79-3.99)**	**3.89 (3.79-3.99)**	**2.79 (2.71-2.87)**
3rd Quartile	49,237 (71.59%)	**8.11 (7.90-8.33)**	**8.06 (7.85-8.28)**	**5.01 (4.86-5.16)**
Most Events	62,990 (86.70%)	**20.98 (20.37-21.60)**	**18.89 (18.29-19.50)**	**9.77 (9.43-10.1)**
**INPATIENT BED DAYS**				
No inpatient admissions	167,213 (53.75%)	Reference	Reference	Reference
Low (1-2 days)	1,408 (82.97%)	**4.19 (3.69-4.76)**	**1.87 (1.64-2.14)**	**1.68 (1.47-1.93)**
Moderate (3-31 days)	8,714 (82.31%)	**4 (3.81-4.21)**	1.06 (1.00-1.11)	1.01 (0.95-1.07)
High (32+ days)	16,281 (88.79%)	**6.81 (6.51-7.14)**	**1.57 (1.49-1.66)**	**1.32 (1.25-1.39)**

*All variables listed in this table had a strong association with the outcome variable (p<.0001), assessed by likelihood ratio tests.
[1]Adjusted for service contact variables (no. of events and inpatient bed days)
[2]Adjusted for all other variables in the table

For peer review only - http://bmjopen.bmj.com/site/about/guidelines.xhtml

FIGURE CAPTIONS

Figure 1: A step-by-step illustration of the methods used for the occupation application development and evaluation, with the number and types of documents used at each step.

Figure 2: The process undertaken by the occupation application when text-mining occupations from the clinical free-text text.

Figure 3: The study population selection and extraction results from text-mining occupations from the Clinical Record Interactive Search case register.

For peer review only

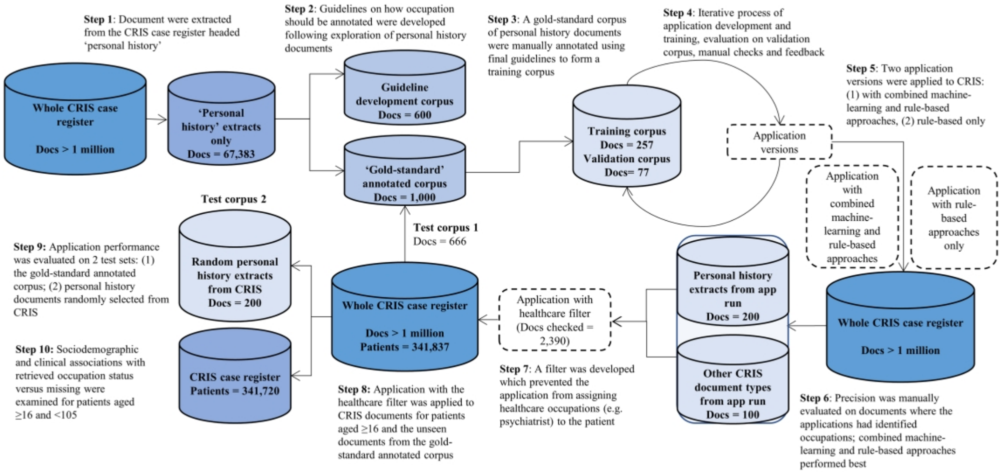

Figure 1: A step-by-step illustration of the methods used for the occupation application development and evaluation, with the number and types of documents used at each step.

254x121mm (300 x 300 DPI)

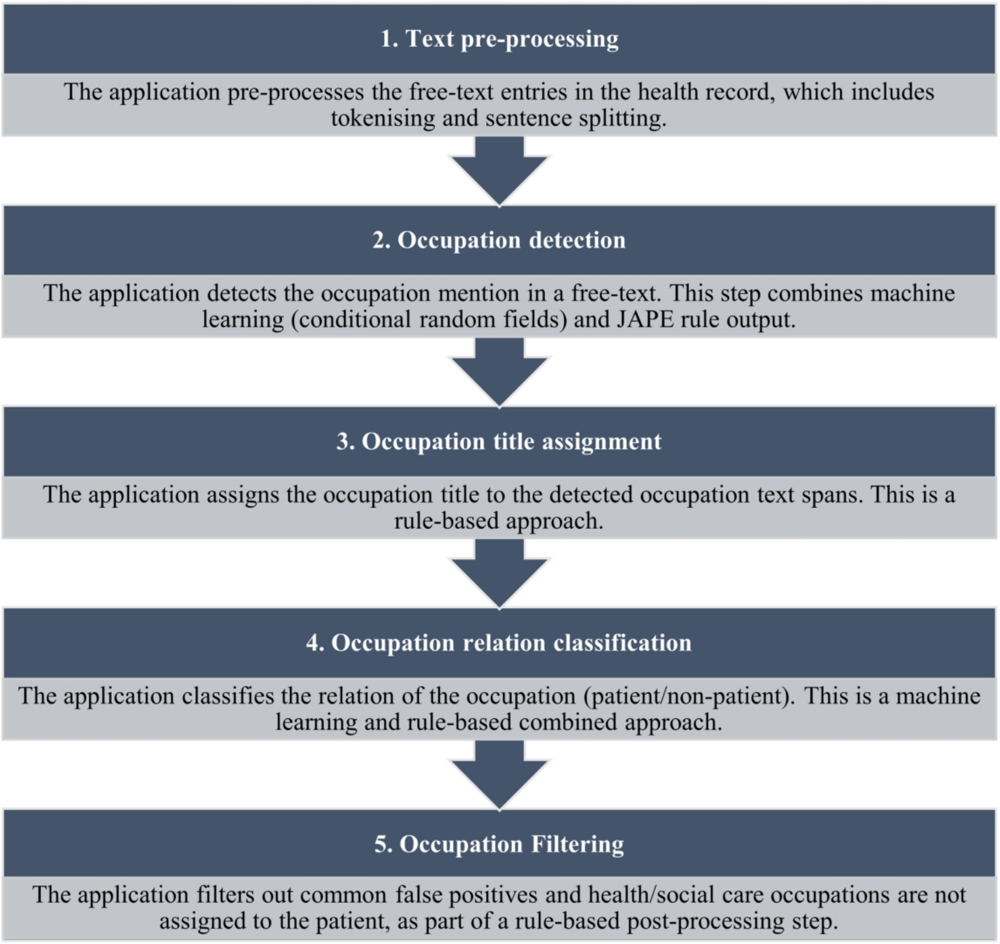

Figure 2: The process undertaken by the occupation application when text-mining occupations from the clinical free-text text.

153x145mm (300 x 300 DPI)

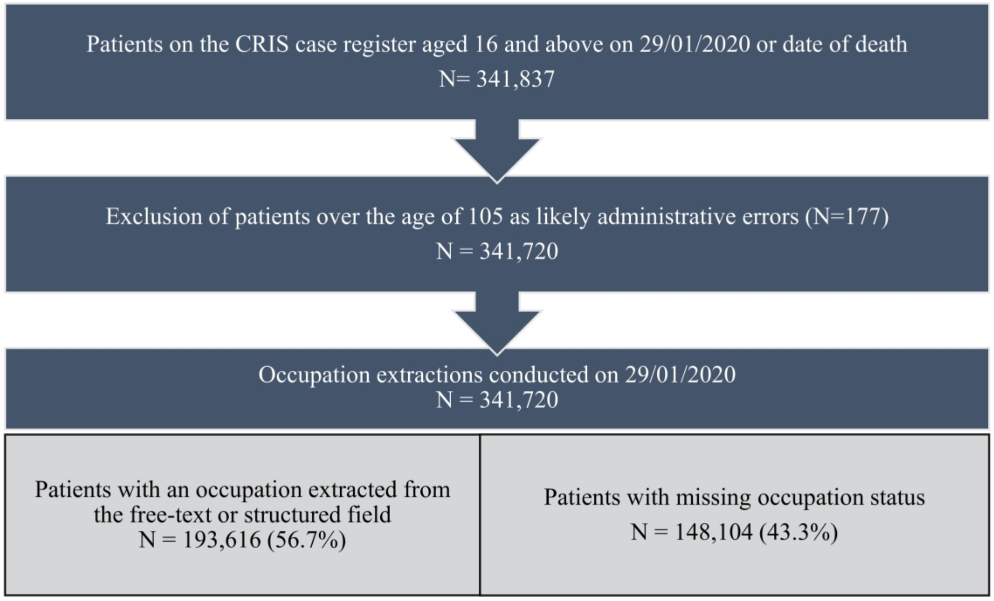

Figure 3: The study population selection and extraction results from text-mining occupations from the Clinical Record Interactive Search case register.

174x106mm (600 x 600 DPI)

Supplementary File 1: Descriptions of the datasets used in the development, testing and implementation of the occupation application

**Application Development and Testing Datasets**			
	**Type of document**	**Document count**	**No. of Occupation Annotations (manual)**
Validation corpus	Personal history	77<br>(+256 documents used in training)	405
Testing corpus 1: with vs without machine-learning comparison	Personal history + other CRIS documents	200	521
Testing corpus 2: gold-standard annotated documents	Personal history	666	3,429
Testing corpus 4: Unannotated documents	Personal history	200	442
**Application Implementation Dataset**			
	Type of document	**Patient count**	**No. Of Occupation Extractions (application)**
CRIS case register of patient records aged >=16	Attachments	341,720	21,321,757 (all relations)
	Events		
	Correspondence		
	Discharge Notification Summaries		
	History		

For peer review only

	Mental State Formulations		
	Presenting Circumstances		
	Risk Events		
	Social Situation		
	Ward Progress Notes		

Table 1: Descriptions of the datasets used in the development, testing and implementation of the occupation application

For peer review only

OCCUPATION ANNOTATION GUIDELINES

Authors:

Background – Natasha Chilman & Esther Tolani

Annotation rules – Esther Tolani, Angus Roberts, Zoe Chui, Karen Birnie, Lisa Harber-Aschan, Billy Gazard, Anna Kolliakou & Jayati Das-Munshi

General Tips – Esther Tolani

Appendices – Natasha Chilman & Anna Kolliakou

With thanks to Shirlee MaCrimmon for annotation support.

Supplementary File 2 to accompany the BMJ Open paper, "Text-mining occupations from the mental health electronic health record: a natural language processing approach using the Clinical Record Interactive Search (CRIS) platform to identify patient occupations in south London, UK."

Background 2
Setting 2
Development 2
Annotation rules 5
Annotation rules 6
Occupation 8
Subject of Occupation 13
Exclusion Criteria 15
General Tips 16
References 17
Appendix 1 17
A timeline of actions leading to guideline and application development 17
Appendix 2 22
Annotation Guidelines Version 1 22

For peer review only

For peer review only - http://bmjopen.bmj.com/site/about/guidelines.xhtml

Background

The CRIS-occupation-application has been developed to enable researchers to extract occupations from the Clinical Record Interactive Search (CRIS) case register. When using the occupation application, it is important to consider how it has been trained and tested to allow for appropriate use of the application and accurate interpretation of results. These guidelines provide clear and transparent rules which specify how occupations should be annotated manually in free-text EHRs, which then informed the development of the occupation application, and a gold-standard against which the application was evaluated against.

Setting

These occupation annotation guidelines were developed over the years 2017-2020 for use on psychiatric clinical texted accessed through the Clinical Record Interactive Search (CRIS) application. CRIS is a large de-identified case register of electronic health records, comprising of the Electronic Patient Health Journal notes used in South London and Maudsley NHS Trust (SLaM). SLaM is the largest unit mental health provider of secondary services in Europe, serving 1.3 million people across the London boroughs of Lambeth, Southwark, Lewisham and Croydon. The SLaM CRIS case register stores over 350,000 patient records to date, and encompasses a range of secondary mental health services (including inpatient and community mental health services) [1]. Whilst this annotation guideline was written following the exploration of CRIS text extracts, we also recommend that the guidelines can be used as a starting point when extracting occupations from other CRIS systems and psychiatric Electronic Health Records (EHRs) in the UK.

It is important to remember that EHRs are a secondary routine data resource in research, they are used primarily for a practical purpose by clinicians to document patient-level information. This context should be kept in mind when considering the complexity of annotating occupations.

Development

Here we summarise the actions taken to develop the guidelines and describe how the guidelines have changed over time. This is also detailed further in the development timeline (Appendix 1).

These guidelines were based on the 'personal history' sections of the free-text entries. When clinicians use 'personal history' as a header in the free-text fields in CRIS, the text which follows typically includes information on the patient's upbringing and family life,

For peer review only - http://bmjopen.bmj.com/site/about/guidelines.xhtml

For peer review only

education and – most importantly for our interests – occupation. Personal history sections were chosen as the best place to start when examining how occupation is described in the free-text fields in CRIS. An application previously developed by Dr David Chandran in the Biomedical Research Centre was used to extract personal history documents from CRIS to develop and test the annotation guidelines. A 'document' is a single section of a free-text field in CRIS, for example a letter attachment or event progress note. One patient may have more than one personal history section in their record.

Initial guidelines were drawn from the exploration of 100 personal history documents and team discussions. From the first draft, the occupation annotation guidelines were developed based on the premise that when an occupation is annotated in the free-text, two components must be specified: the occupation (feature) and subject of the occupation (relation). Occupation is a complex concept and can be written as a job title (e.g. a waiter) or a description of a work activity (e.g. serving tables).

The guidelines were developed through an iterative process of document annotation, team discussions and rule development (Appendix 1). 600 personal history documents were annotated throughout this process which informed and tested the sufficiency of the guidelines to instruct occupation annotation. Out of these 600 documents, 250 personal history documents were double annotated. Inter-annotator agreements were calculated throughout the guideline development stages to assess whether the guidelines were sufficient for occupation to be annotated consistently (Appendix 1). By November 2017, 200 further personal history documents were double-annotated with good inter-rater reliability between two manual annotators, with a Cohen's Kappa statistic of 77% for occupation and 72% for relation. This is considered a good level of agreement. 800 documents were then annotated by a single annotator using the latest guideline and together these formed the 1000 document gold-standard annotated document corpus. This corpus was later used for application development (forming the training corpus).

To demonstrate how the guidelines have changed over time, please see Appendix 2 which shows the Guidelines Version 1 (GV1). When compared to the current guidelines, a significant level of detail has been added since the initial draft. For example, there is now a section the beginning of the guidelines stating which parts of a sentence describing occupation should be annotated. Whilst re-drafting the guidelines, an 'additional information' column was added to give further detail on how the annotation rules work, which researchers found helpful when completing annotations. The later drafts of the guidelines also add a 'blank' annotation rule: if the occupation title can be inferred by the text itself then the occupation feature should be left empty, and the relation was determined by the sentence structure. This was important when later evaluating the application, as a bespoke GATE evaluation package was used to take this rule into account. All changes that were made to the annotation guideline throughout the development process were agreed within the research team.

For peer review only - http://bmjopen.bmj.com/site/about/guidelines.xhtml

The following guideline is the final annotation guideline document. Whilst there were some small formatting changes made during application development (Appendix 1), the rules in this guideline were used when annotating the 1000 gold-standard training and testing corpus for the application.

For peer review only

For peer review only - http://bmjopen.bmj.com/site/about/guidelines.xhtml

Annotation rules

Esther Tolani, Zoe Chui, Karen Birnie, Angus Roberts, Anna Kolliakou, Jayati Das-Munshi, Robert Stewart

These guidelines outline the process for annotating occupation status in GATE. The term(s) highlighted should be the word(s) in the free text which indicate(s) the occupation of an individual. After reading the free text, annotations should be made on the word(s) which is (are) related to an employment status or an occupation: job or profession. For all cases, each annotation will have the following features: **occupation and subject of occupation (relation).**

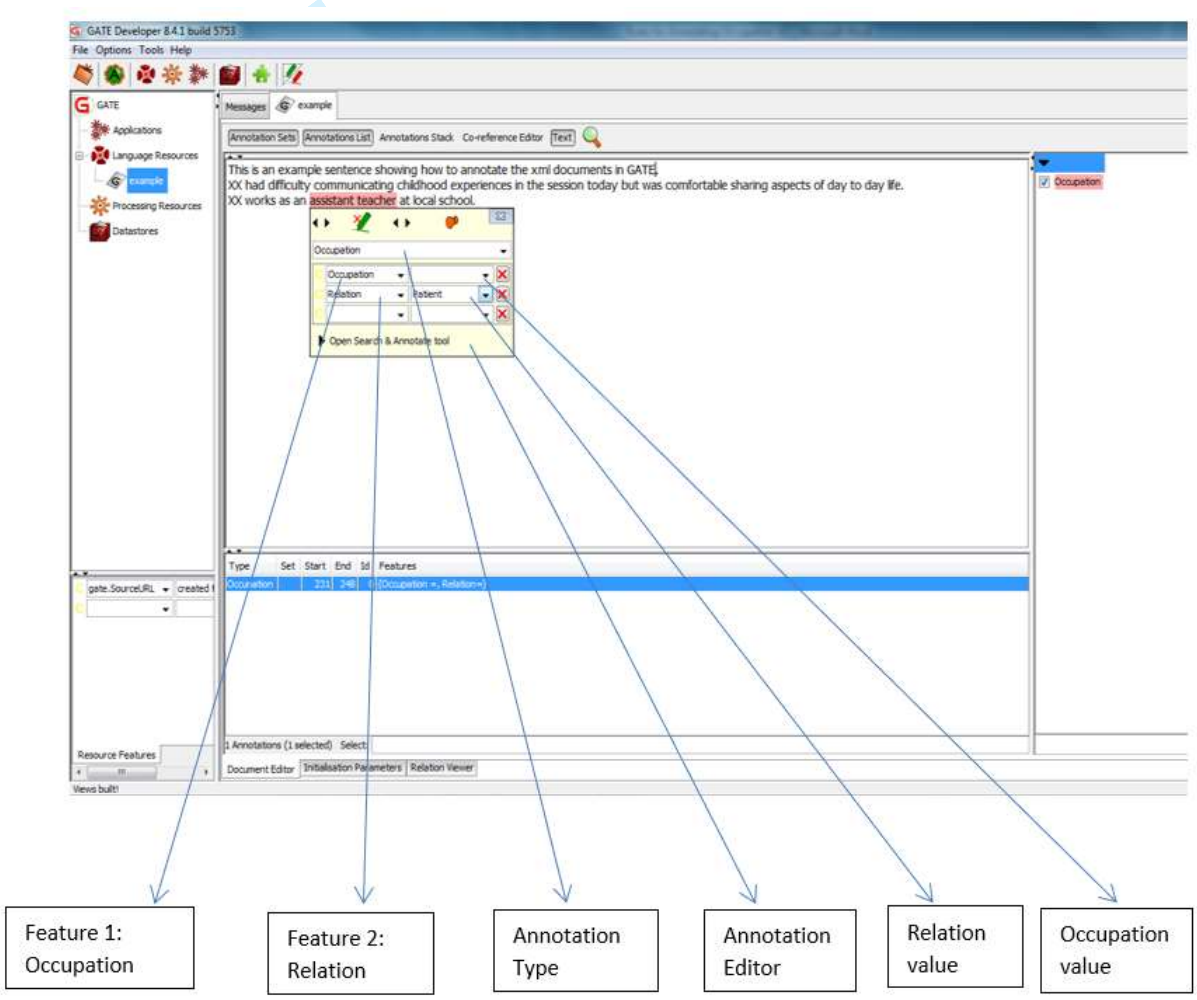

Figure 1: A labelled example illustrating how occupation is annotated in GATE software

Sentence Structure of Annotation

The annotation should be made on adjectives, nouns and verbs in the sentence.

- Title of Occupation

Titles of occupations are always nouns.

Adjectives should only be annotated when they are part of the occupation type or necessary for describing the occupation e.g. assistant manager, senior consultant. The annotation value is left empty when occupation can be inferred from the exact annotated text.
Example:
XX worked as an assistant teacher – occupation value: empty.
She is a mental health nurse – occupation value: empty
Annotate the adjective and noun.

- **Description of Occupation**
    A) Description of occupation consists of verbs referring to work activities.

Annotate text following:
    1) Works for/in/as/at…

Works for real estate - occupation value: estate agent
Works for British Gas - occupation value: British Gas worker
Works for investment bank – occupation value: investment bank worker

    2) Job/Role involves, has to do with, includes…

Job involves cleaning houses – occupation value: house cleaner
Role involves writing, teaching – occupation value: writer, teacher

    3) Verbs indicating membership

Joined the navy – occupation value: navy officer
Example:
XX worked joined the army after moved to the UK – occupation value: army officer.
Annotate the verb and noun because the noun or verb alone does not describe the occupation sufficiently.

Annotation rules

An occupation or description of work should be annotated regardless of whether it is current or past. However, text indicating whether occupation or description of work is current or past is not required for the annotation unless it offers information on the stability/transience of the occupation.

Examples:
XX is not working at the moment – occupation value: unemployed
XXX has been working as a chef for 3 years- occupation value: chef

For peer review only - http://bmjopen.bmj.com/site/about/guidelines.xhtml

XXX worked briefly or worked for a few months or worked every summer – occupation value: other

Do not annotate:

- Punctuation
  - e.g. full stops, semi-colons…
- Adverbs
  - e.g. happily, works hard…
- Articles in front of occupation
  - e.g. the, as, an, a…
- Conjunctions
  - e.g. and, but, if…

*[**UNLESS** these are articles and conjunctions in a double annotation as further below]*

- Adjectives when describing a quality assigned to a job
  - e.g. experienced teacher, qualified electrician
- Verbs that precede title of occupation
  - e.g. became, moved to, promoted to, went to, decided to, etc.
- Text around title of occupation describing place of work **unless** text around title of occupation refers to a field or sector
  - e.g. assistant manager for a phone company – value empty
  - e.g. assistant manager in sales – value fill Sales Assistant Manager
- Time frames or duration of work
  - e.g. worked for 5 years, was a chef in 1995, has worked, is not working

*[**UNLESS** it offers information on the stability/transience of the occupation ie worked briefly or worked for a few months or worked every summer]*

Double annotation:

In the case of two joint occupation descriptions, annotate the same text twice and give a different value each time.

Examples:
Annotate once: he worked in a clothes shop and a kitchen – occupation value: retail worker
Annotate twice: he worked in a clothes shop and a kitchen – occupation value: other - kitchen

Please use this double annotation as sparingly as possible and not when clearly stated occupations or different occupations/work descriptions are joined as below.

Examples:
He worked as a chef and cleaner – two annotations with blank values

For peer review only - http://bmjopen.bmj.com/site/about/guidelines.xhtml

He worked on building sites and roofing - two annotations with first value 'labourer' and second 'labourer' or 'other'.

Long job descriptions:
Sometimes clinical record notes are written in a rich and speech-like manner. In cases like this, it is best to annotate a longer piece of text then risk leaving out valuable information.

Examples:
She has worked for only 1 and half year in her life in a wine bar 28yrs ago – occupation value: other- bar

He used to work every summer with his brother at a car wash – occupation value: other-car wash

Occupation

For the occupation value, a title for the work described should be entered. If no title can be created from a work description, 'other' should be entered in the occupation value. In addition, if the title is identical to the work described (job can be inferred from the annotated text), the occupation should be left empty.

**Rules for annotating Employment Status**				
Rule	Rule Description	Example	Occupation Value	Additional Information
Description of job is given, without job title	Annotate with closely related description	Daily role involves operating the machines	Machine operator	
Multiple occupations	All occupations mentioned in the free text (personal history) should be annotated even if they are or appear to be repetitions of an occupation already mentioned within the same history	Chef, 7.5-tonne truck driver<br><br>Worked as kitchen assistant…he helped in a kitchen for 6 months<br><br>She was a teacher…enjoyed her work as a teacher	[blank]<br>[blank]<br><br>Kitchen assistant<br>Other-kitchen<br><br>[blank]<br>[blank]	For chef, truck driver, kitchen assistant and teacher the occupation value should be left empty because the work descriptions are identical to the title that should be given. For 'helped in a kitchen' the occupation value should be 'other'
Related occupations	Annotate all occupations which are mentioned which are	Worked as a social worker and later became a manager	[blank]<br>[blank]	The occupation value should be left empty because the work

For peer review only - http://bmjopen.bmj.com/site/about/guidelines.xhtml

	associated with the progress of the same job			descriptions social worker and manager identical to the titles that should be given
Place, sector or employer is mentioned without occupation	Annotate the company or sector	XX works for the council<br>He has been with his present boss for a while	Council worker<br><br>Other	Annotate the company, sector or employer
Loose description of job role which cannot be titled	Annotate the reference to odd jobs which have relevance	XX does various jobs which include, tiling, plumbing...	Tiler, plumber	Annotate the word referring to the odd job

The section below outlines how to annotate the alternative employment statuses: student, retired, self-employed, unemployed, carer, homemaker and other.

**Rules for annotating Student Status**				
Rule	Rule Description	Example	Occupation Value	Additional Information
Student (full time/part time)	Annotate term student or a description of full time/ part time study. Include training/vocational courses.	XX is currently studying XX at university	Student	
		He trained as a bricklayer	Student<br>[blank]	Two annotations are made to capture student status and occupation value of empty for bricklayer
		He trained in art	Student	'Trained' is annotated by itself whereas 'attended', 'did' 'degree' or 'undertook' need extra information annotated because out of context they wouldn't be sufficient by themselves
		She attended university	Student	
		Has a degree in Physics	Student	
		He did a Masters in Psychology	Student	
		Left University in 1995	Student	

		Graduated with a degree in maths<br><br>He undertook the early career researcher training scheme	Student	
**Rules for annotating Retired Status**				
Retirement	Annotate the term retired or description of retirement	Worked until retirement	Retired	
**Rules for annotating Self-Employed Status**				
Self-employed without job description	Annotate the term or description of self-employed	Patient is self-employed	[blank]	The occupation value should be left empty because the job title self-employed is stated
		He owns a number of properties and shops	Self-employed	The occupation value should be self-employed. One annotation.
Self-employed with job description or business/property owner	Annotate the term or description of self-employed and job description	Patient is a self-employed builder	[blank] [blank]	These should be two separate annotations (self-employed and builder). Occupation values should be left empty because the job titles self-employed and builder are stated
		He owns a number of properties and shops	Self-employed	One annotation

**Rules for annotating Other Employment Status**	

For peer review only - http://bmjopen.bmj.com/site/about/guidelines.xhtml

Difficult to define or job/ job role not stated<br>Simple reference to work	Annotate the verb 'work' or the noun 'job'	Works occasionally on weekends<br>Has had a few other jobs<br>He worked there for 4 years and then left<br>He worked in 1995<br>He worked hard all his life<br>He worked briefly when younger<br>He had a satisfactory job<br>She had numerous jobs<br>He has a creative job<br>She has had three other jobs<br>He did about 8 jobs	Other	Annotate the verb work by itself unless followed by an adverb providing more information about the work itself ie occasionally, the number of jobs ie numerous or the quality ie hard, creative
Sector is not mentioned	Statements not referring to a specific sector or industry should not be annotated	XX moved to the private sector	Other-private	
Army/Navy occupations	Annotate relevant word/ phrase	XX joined the army	Army officer	Always annotate as army or navy officer
Job or occupation relating to shops	Annotate relevant word/ phrase	XX worked part-time in WHSmith	Retail worker	Always annotate as retail worker
Sector or place of work is mentioned but unclear what job the subject undertook	Annotate relevant word/phrase	XX joined his brother in construction<br><br>XX worked in a kitchen	Other-construction	It is not clear what job in construction the patient did so occupation value is given 'other'
**Rules for annotating Unemployed Status**				
Unemployment	Annotate the term unemployed or the description of unemployment	XX has not worked for several years<br>XXX does not work anymore	Unemployed	Unemployment is usually stated in various ways. If the word unemployed is

For peer review only - http://bmjopen.bmj.com/site/about/guidelines.xhtml

		XXX lost his job<br>XXX ran out of work a year ago<br>XXX is currently not working<br>XXX got sacked<br>XXX was made redundant<br>XXX stopped working<br>XXX cannot remember the last job she had<br>Last job was about 5 years ago<br><br>XXX is unemployed	[blank]	annotated, the annotation value should be blank
**Rules for annotating Homemaker Status**				
Housewife househusband	Annotate the term that states that an individual is a homemaker	Mother was a housewife…	[blank]	The occupation value should be left empty because housewife status is stated
**Rules for annotating Carer Status**				
Carer	Annotate the term carer or the description of care role	XX is a carer for elderly mother<br><br>XXX was a carer	Carer<br><br>[blank]	Annotation value of carer should be entered if text annotated includes who the person cared for is. In the second case, where this is not stated, the occupation value is left empty because carer status is stated
**Rules for annotating Volunteer**				
Volunteer	Annotate the noun volunteer or the verb volunteering	XX volunteered with the	Volunteer	

For peer review only - http://bmjopen.bmj.com/site/about/guidelines.xhtml

		council once a week		
**Rules for annotating National Service**				
National Service	Annotate the noun national service and the verb preceding	He joined national service<br><br>He did his national service<br><br>He finished national service	Other – national service	
**Rules for annotating illegal activities**				
Prostitution	Annotate relevant word/phrase	XX was working as a prostitute	Sex-worker	Always annotate as sex-worker
Jobs of questionable status/legality	Text referring to income generating jobs that might not be legal	He was a brothel owner<br><br>She made money from dealing drugs	Other- brothel<br><br>Other – drug dealing	Other plus an indication of place or type of work

Subject of Occupation

The relation value should state who the occupation refers to/who carries out the job described. In most cases, the occupation belongs to the patient. The occupation can also belong to the parent/carer of the patient, spouse, relative or other.

**Rules for annotating Subject of Occupation**				
Rule	Rule Description	Example	Relation Value	Additional Information
Patient	The occupation annotated should belong to the patient	Patient was a butcher for XX years	Patient	
Parent/ Carer / Guardian	The occupation annotated should belong to the father or mother of the patient.	Father works as a mechanic	Father	
Spouse	The occupation annotated should belong to the spouse	Husband works for the government doing research	Spouse	The occupation of the spouse should still be recorded even if the text suggests they are no longer together
Relative	The occupation annotated belongs to a	XX's brother discussed the issues	Brother	Relations include: sibling,

For peer review only

	family member of the patient who is not the parent/carer or spouse	faced being XX's carer and working as a shop assistant…		cousin, aunt, uncle, niece, child, nephew, and grandchild
Girlfriend/Boyfriend/ Partner	The occupation annotated should belong to the patient's girlfriend/boyfriend	XX's girlfriend was a carer for the elderly	Girlfriend	
Other	The occupation annotated does not belong to the patient or patient's relative	The nurse came round to see XX	Other	

For peer review only

For peer review only - http://bmjopen.bmj.com/site/about/guidelines.xhtml

Exclusion Criteria

Rule	Rule Description	Example	Value	Additional information
Future Plans	Future plans to work should not be annotated	XX plans to start role	No annotation	
Hypothetical statements	Text referring to hypothetical scenarios or worries about losing job	XX said he would have left his job if he thought he couldn't cope	No annotation	
		XX was worried he was going to get sacked	No annotation	
		She would have quit if they hadn't given her a raise	No annotation	
When 'work' is used as an adjective		She had great work ethics	No annotation	
		Her work performance deteriorated		
		She didn't like her work colleague		
Describing quality of work without explicitly stating having one		Her job was really good		
		He didn't enjoy working there		

For peer review only

For peer review only - http://bmjopen.bmj.com/site/about/guidelines.xhtml

General Tips: THINK LIKE AN OCCUPATION MACHINE!

1) The machine doesn't have any context

We annotate personal history segments which, if rich, give us a good idea of an individual's story. The machine does not have that reference and if, for example, we annotate 'stopped' in "he worked for 5 years and then stopped" as 'unemployed' we are essentially teaching it to recognise the word 'stopped' as referring to unemployment. Imagine what will happen when we run this application all over CRIS! Ask, if unsure - does the machine understand the annotation I have assigned regardless of context? What will happen if it learns to recognise it as such in another context?

2) The machine loves more of the same

You come across a personal history segment that has 'worked' 3 times, 'labourer' 2 times, 'jobs' 4 times and 2 'sacked'. The machine doesn't know that these have been repeated as it has no context. Also, the more 'labourers' it gets fed, the more it will learn to unequivocally recognise them automatically in any context. Annotate them all!

3) The machine is as smart as you

If you feel you are spending too long making annotation decisions or find a rule that is making your annotations inconsistent, the machine will think the same. Ask questions no matter how silly they seem!

For peer review only

For peer review only - http://bmjopen.bmj.com/site/about/guidelines.xhtml

References

1. Perera, G., et al., *Cohort profile of the South London and Maudsley NHS Foundation Trust Biomedical Research Centre (SLaM BRC) case register: current status and recent enhancement of an electronic mental health record-derived data resource.* BMJ open, 2016. **6**(3): p. e008721.

Appendix 1

A timeline of actions leading to guideline and application development

Natasha Chilman, Anna Kolliakou

Date	Action	Outcome
July 2017	Preliminary meeting with research team<br><br>Esther annotated 100 personal history extracts with no guidelines<br><br>Feedback on annotations and guideline ideas discussed by research team	Development of GV1 (Guidelines Version 1)
August 2017	Anna M annotated 50 of the above 100 extracts using GV1, recommended changes	Development of GV2
August 2017	Comments given by research team on GV2	Development of…<br>GV2.1<br>GV2.2<br>GV2.3<br>GV2.4
September 2017	1,262 personal history documents extracted:<br>- Esther annotated 500 using GV2.4<br>- Shirlee double-annotated 200 of these using GV2.	Inter-annotator agreement for 200 double-annotated documents: Cohen's Kappa calculated by GATE = 72% for occupation, 87% for relation<br><br>Development of GV2.5
October-November 2017	40 case examples were written by Anna K and annotated by Anna K, Lisa, Billy, Shirlee and Angus.	Collective agreements made on rules<br><br>Started development of GV2.6
November 2017	Above case examples were given to Karen and Zoe who annotated according to GV2.5	Collective agreements made on rules

For peer review only - http://bmjopen.bmj.com/site/about/guidelines.xhtml

		Finished development of GV2.6
November-December 2017	1000 new personal history documents extracted:<br>- Karen annotated all 1000 using GV2.6<br>- Zoe double-annotated 200 of these using GV2.6	Inter-annotator agreement for 200 double-annotated documents:<br>Cohen's Kappa =<br>77% for occupation<br>72% for relation<br><br>In total, 1000 documents = 'gold-standard' annotated corpus
March 2018	GV2.6 was finalised and so was re-named GV3. The 1000 annotated documents were stratified by gender, length of extract, and occupation feature type (labelled as 'other' vs 'non-other' – see guideline for more detail).	334/1000 stratified annotated extracts were sent to Xingyi (University of Sheffield) to develop the application: 257 were used as a training set, 77 were used as a validation set.
April 2018	Application version 1 (AV1) created by Xingyi, sent to Anna K who manually checked application output on the 77 test corpus and precision, recall and F-measures were calculated by GATE evaluation package, feedback provided to Xingyi on application areas for improvement.	Development of AV2
April 2018	As above: AV2 ran on 77 documents, manually checked and measures calculated by GATE evaluation package, feedback provided.	Development of AV3
June 2018	As above: AV3 ran on 77 documents, manually checked and measures calculated by GATE evaluation package, feedback provided.	Development of AV3.1
August 2018	AV3.1 included three different application versions, all ran on 77 documents, manually checked and measures calculated by GATE evaluation package, feedback provided.	Development of AV3.2
November 2018	AV3.2 (one version) was run on 77 documents. The GATE evaluation package was under-estimating the performance of the application, as it classified that if an occupation feature was 'blank' then it was not labelled correctly. Please see guideline for instructions on use of 'blank' feature annotations. These type of annotations came up often in the text.	Development of AV3.3

For peer review only - http://bmjopen.bmj.com/site/about/guidelines.xhtml

	A new evaluation package ('revised' GATE evaluation) was created which correctly identified 'blank' annotations as a hit. This increased the F-measure and was felt to more accurately reflect the application performance when checking the output manually.<br><br>A small formatting change was made to the guideline, creating GV4, but there was no change in rule content.<br><br>Further feedback sent to Xingyi.	
November-December 2018	AV3.3 was run on the 77 documents, manually checked and F-measures calculated by revised GATE evaluation package, feedback sent to Xingyi.<br><br>A decision was made that the 77 documents needed to be re-annotated which was completed by Anna K in December 2018.	AV3.3 was updated
January-February 2019	Updated AV3.3 was run on both newly annotated 77 documents and previously annotated 77 documents. Barely any difference found in impact on F measure (a very small increase: old annotations F=0.890, new annotations F=0.896).<br><br>Updated AV3.3 run on newly annotated 77 documents, manually checked and F measures calculated by revised GATE evaluation package, feedback sent to Xingyi.	Development of AV3.4
April 2019	As the application was performing reasonably well on the 77 personal history documents, AV3.4 was run on the whole of CRIS. Anna K eyeballed the output and sent feedback to Xingyi for areas for improvement.	AV3.4 was updated to two versions: AV3.4(with machine learning) and AV3.4Revised (without machine learning)
June-July 2019	Both AV3.4 and AV3.4Revised were run on whole CRIS. Anna and Natasha manually checked 200 random personal-history-only documents, and 100 random CRIS documents. Areas for application improvement were sent to Xingyi.	Development of AV4
August 2019	AV4(ML) and AV4(Revised) were run on the whole CRIS. Training corpus of 77 documents was used to evaluate application on GATE. Anna and Natasha manually checked 200 random personal history-only documents, and 100 random CRIS documents (test corpus).	Results from performance of both applications on training corpus and test corpus is available in Supplementary File 3. Application reached good levels of performance

For peer review only - http://bmjopen.bmj.com/site/about/guidelines.xhtml

		(precision and recall all >0.79 on a test corpus). The machine learning application performed slightly better so this was chosen over the rule-based approach. However occupation ownership remained an issue, where many of the occupations retrieved belonged to people other than the patient e.g. clinicians. The application did not consistently annotate the relation of the occupation correctly, for example often 'psychiatrist' was annotated as belonging to the patient.
September-November 2019	Following occupation ownership issues identified in the manual evaluation, team meetings were held and it was decided to add an occupation 'filter' to the application. This is a list of occupations which have the most common incorrect relations (e.g. psychiatrist, social worker) – where the application incorrectly annotates the occupation as belonging to the patient. The occupations included in the filter will be assigned a 'other' relation, rather than 'patient' relation. This will mean that we can be more confident that the occupation extracted belongs to the patient. The team reflected that we may miss a small number of true positives this way (e.g. psychiatrists who are patients), but the risk of retrieving incorrect patient occupations is greater, plus healthcare professionals often go to different occupational services for mental health support so are less likely to be included in this sample of electronic health records.<br><br>Method:<br>- Natasha extracted occupations with ≥100 annotations across CRIS. She then sorted these occupations into 3 categories: those which should definitely be added to the filter (e.g. psychiatrist), those which she was not sure about (e.g. interpreter) and those not to add to the filter (e.g. construction).<br>- Out of those which she was not sure about, Natasha checked between 10-40 documents for the number of true positives retrieved by the application	AV4 with machine learning was updated by Xingyi to include the occupation filter, where the occupations on the filter list were assigned the relation 'other' rather than patient.

For peer review only - http://bmjopen.bmj.com/site/about/guidelines.xhtml

	(where the occupation was annotated correctly as belonging to the patient). During this process Natasha checked a total of 2,390 documents.<br>- Jay and Anna then went through this list to make collective decisions with Natasha on the unsure occupations. The filter list of occupations was then sent to research team for approval, then sent to Xingyi to add to the app.	
January-February 2020	The application was run over the whole of CRIS with the health/social care occupation filter applied.	Natasha firstly checked accuracy of 400 annotations made by the application: 200 from personal history documents only (precision all annotations = 96.00%, precision patient annotations only = 97%), and 200 annotations over other CRIS document types (precision all annotations = 93.00%; precision patient annotations only = 66%). Of the last estimate, many false positives were for occupation annotations for 'other'.
February 2020	Natasha checked 200 'other' occupation annotations to test the accuracy of this annotation and whether it should be excluded.	Precision for 'other' annotations only reached 23.5%. The false positives for this annotation seemed to fit 3 categories: text about job-seeking (e.g. looking for work), text about working on health/personal goals (e.g. working on his anxiety) or other incorrect annotations (e.g. blood work).
March 2020	Natasha looked at recall and precision more closely. Jyoti ran the application over the personal history table in gate (with extracts accessed via Dave Chandran's personal history app). Natasha selected 200 random documents from this personal history table, annotated them according to this occupation annotation guideline (excluding 'other' annotations), and then checking to see whether the app had identified these occupations (recall) or had identified any false positives (precision). As patient occupations are only mentioned rarely in the clinical record, it was not feasible to do a recall/precision check on all other types CRIS documents, therefore personal history	When looking at all occupation relation annotations, the app had a precision level of 90.04 and recall level of 85.77. When looking at patient relation only annotations, the application reached precision of 77.33 and recall of 79.37.

For peer review only - http://bmjopen.bmj.com/site/about/guidelines.xhtml

	documents are chosen as a targeted and feasible document to check.	

Appendix 2

Annotation Guidelines Version 1

Date: 04/08/2017

This guideline outlines the process for annotating occupation status in GATE. The term highlighted should be the word(s) in the free text which indicates the occupation of an individual, as described in the personal history of the patient. After reading the free text, annotations should be made on the word(s) which are related to an employment status or an occupation: job or profession. For all cases, each annotation will have the following features: **occupation and subject of occupation.** The exclusion criteria outline when no annotations should be made.

**Rules for annotating Occupation Status**			
Rule	Rule Description	Example	Actual Annotation
Multiple occupations	All occupations mentioned in the free text (personal history) should be annotated	Chef, 7.5-tonne truck driver	Occupation: chef, truck driver
Working role is given, without occupation mentioned	Annotate with closely related description	Daily role involves operating the machines	Occupation: production worker/machine operator
Related occupations	Annotate all occupations which are mentioned which are associated with the progress of the same job	Worked as a social worker and later became a manager	Occupation: social worker, social work manager
Place or sector is mentioned without occupation	Annotate the company or sector	XX works for the council	Occupation: council worker
Loose description of job role which cannot be titled	Annotate the reference to odd jobs which have relevance	XX does various jobs which include, tiling, plumbing…	Occupation: Tiler and Plumber

For peer review only - http://bmjopen.bmj.com/site/about/guidelines.xhtml

**Rules for annotating Student Status**			
Student (full time/part time)	Annotate term student or a description of full time/ part time study	XX is currently studying XX at university	Occupation: student
**Rules for annotating Retired Status**			
Retirement	Annotate the term retired or description of retirement	Worked until retirement	Occupation: retired
**Rules for annotating Self-Employed Status**			
Self-employed without job description	Annotate the term or description of self-employed	Patient is self-employed	Occupation: self-employed
Self-employed with job description	Annotate the term or description of self-employed and job description	Patient is a self-employed builder	Occupation: self-employed, builder
**Rules for annotating Other Occupation Status**			
Difficult to define or job/role not stated	Annotate relevant phrase	Works occasionally on weekends	Occupation: other
**Rules for annotating Unemployed Status**			
Unemployment	Annotate the term unemployed or the description of unemployment	XX has not worked for several years	Occupation: unemployed

For peer review only - http://bmjopen.bmj.com/site/about/guidelines.xhtml

Supplementary File 3: Machine learning and rule-based approaches to text-mine occupations from the electronic health record

The Occupation Application Pipeline

The occupation extraction application works by implementing 5 steps: 1) Text pre-processing, 2) Occupation mention detection, 3) Occupation title assignment, 4) Occupation relation extraction and 5) Occupation filtering. The pipeline of the application is demonstrated in Figure 1.

For a free-text input, we pre-process the input document through: (1) an English Tokeniser, (2) GATE's Morphological Analyser (lemmatise and tokens), (3) a sentence splitter (as the occupation extraction is conducted at sentence level), (4) a POS tagger (where we obtain part-of-speech for each token, and the part-of-speeches are used as features in later rule and machine-learning modules), and (5) ANNIE Name Entity Transducer (the default Name Entity Transducer embedded in the GATE system; these entities are used as features in later rule and machine learning modules).

After text pre-processing, we detect occupation mentions in the free-text by using: (1) a Conditional Random Field algorithm-based machine learning approach, and (2) a JAPE rule based approach. We combine the results from both approaches to increase the recall level. A rule-based title assignment module is applied to assign the occupation titles (e.g. builder, doctor, etc) for extracted occupation mentions.

When identifying who the occupation belongs to ('relation' extraction), we first extract the relation phrases (e.g. patient, mother, etc) from the surrounding context of the occupation mention. We then use a rule-based and machine-learning (support vector machine)-based classifier to classify the occupation relation. In this application we prefer rule-based relation classifier output to the machine-learning output when available – the machine-learning relationship is only used when there is no output from the rules.

The final step of the pipeline is occupation filtering, which is a rule-based approach to filter out common false positives and health/social care occupations.

For peer review only

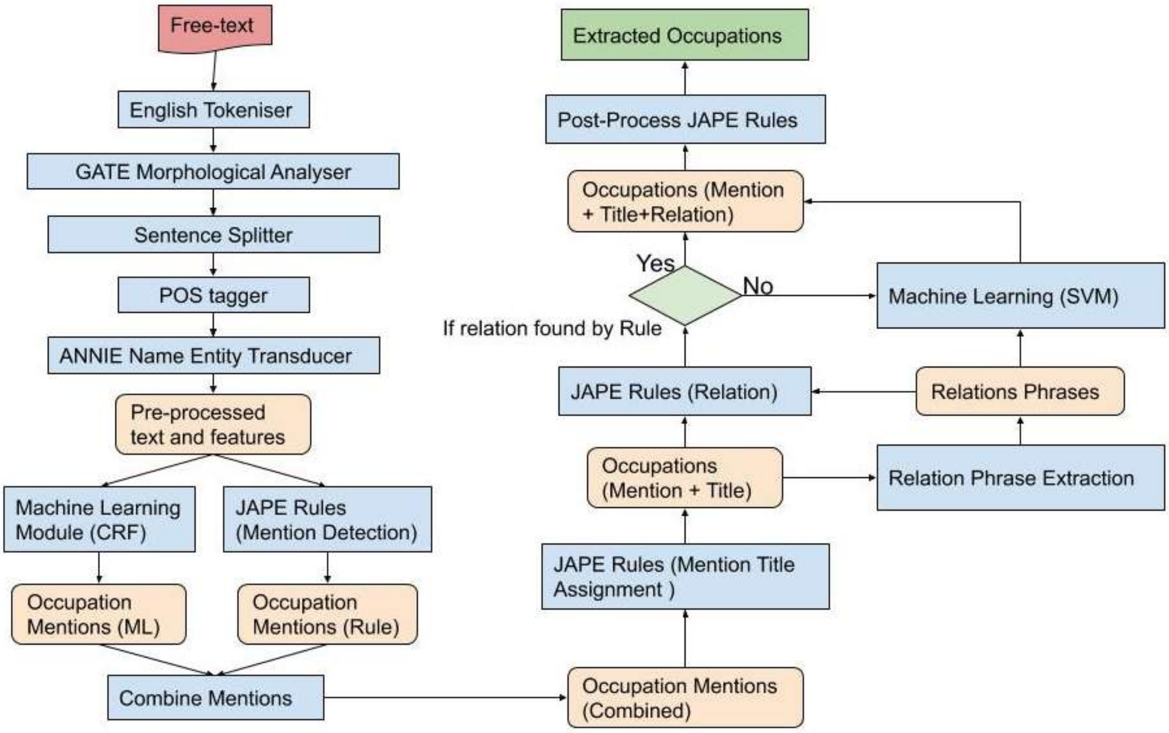

Figure 1: The pipeline of the occupation application. [1]

[1] The red box represents the input text, blue boxes represent NLP modules, light orange boxes represent the intermediate output from the NLP modules and the green box represents the extracted occupation.

For peer review only - http://bmjopen.bmj.com/site/about/guidelines.xhtml

Comparing combined machine-learning and rule-based approaches with rule-based only approaches

During testing we evaluated two versions of the application: one with machine-learning and rule-based combined approaches, and one with rule-based approaches only (without machine-learning). In the application version with rule-based approaches only, all machine-learning components in the occupation application pipeline (Figure 1) were removed.

The two versions of the applications were run over free-text documents in the case register of electronic health records. Where an occupation was identified by at least one of the application versions, we extracted 100 documents which included sections of text entitled 'personal history' and 100 documents which did not include a 'personal history' section (e.g. other 'Events' or 'Attachments'). One document may have multiple occupation annotations – all of which were evaluated. Where an occupation was annotated correctly this was counted as a true positive for occupation precision; where who the occupation belonged to was annotated correctly this was counted as a true positive for occupation relation; and where both were correct this was counted as an overall true positive for precision (table 1).

Both applications performed similarly, however the application with machine learning performed best on both personal history and other document types when assigning the occupation 'relation' (relation precision=0.91 on personal history documents). As the authors wanted to maximise precision regarding who the occupation belonged to (particularly for the patient), this application version was chosen for further developments.

**Documents**	**Application version**	**Precision**	**Occupation precision**	**Relation precision**
100 personal history	With Machine-Learning	0.92	0.96	0.91
	Without Machine-Learning	0.95	0.96	0.85
100 other CRIS document types	With Machine-Learning	0.79	1	0.68
	Without Machine-Learning	0.94	1	0.58

Table 1: Evaluation of occupation applications on the test corpus of documents where the applications had identified an occupation, calculated manually.
**Precision = true positive annotations/all annotations*
*** Occupation precision = true positive occupation titles/all occupation titles*
****Relation precision = true positive relation assignments/all relation assignments*

The RECORD statement – checklist of items, extended from the STROBE statement, that should be reported in observational studies using routinely collected health data.

	Item No.	STROBE items	Location in manuscript where items are reported	RECORD items	Location in manuscript where items are reported
**Title and abstract**					
	1	(a) Indicate the study's design with a commonly used term in the title or the abstract (b) Provide in the abstract an informative and balanced summary of what was done and what was found	Page 1, title<br><br>Page 3, abstract	RECORD 1.1: The type of data used should be specified in the title or abstract. When possible, the name of the databases used should be included.<br><br>RECORD 1.2: If applicable, the geographic region and timeframe within which the study took place should be reported in the title or abstract.<br><br>RECORD 1.3: If linkage between databases was conducted for the study, this should be clearly stated in the title or abstract.	Page 1, title<br><br>Page 1, title<br>Page 3, abstract<br><br>N/A
**Introduction**					
Background rationale	2	Explain the scientific background and rationale for the investigation being reported	Page 5, introduction		
Objectives	3	State specific objectives, including any prespecified hypotheses	Page 5, introduction, paragraph 3		
**Methods**					
Study Design	4	Present key elements of study design early in the paper	Page 5, introduction, paragraph 3<br><br>Figure 1		
Setting	5	Describe the setting, locations, and relevant dates, including	Page 6, materials & methods, paragraph 1		

For peer review only

		periods of recruitment, exposure, follow-up, and data collection			
Participants	6	*(a) Cohort study* - Give the eligibility criteria, and the sources and methods of selection of participants. Describe methods of follow-up<br>*Case-control study* - Give the eligibility criteria, and the sources and methods of case ascertainment and control selection. Give the rationale for the choice of cases and controls<br>*Cross-sectional study* - Give the eligibility criteria, and the sources and methods of selection of participants<br><br>*(b) Cohort study* - For matched studies, give matching criteria and number of exposed and unexposed<br>*Case-control study* - For matched studies, give matching criteria and the number of controls per case	N/A	RECORD 6.1: The methods of study population selection (such as codes or algorithms used to identify subjects) should be listed in detail. If this is not possible, an explanation should be provided.<br><br>RECORD 6.2: Any validation studies of the codes or algorithms used to select the population should be referenced. If validation was conducted for this study and not published elsewhere, detailed methods and results should be provided.<br><br>RECORD 6.3: If the study involved linkage of databases, consider use of a flow diagram or other graphical display to demonstrate the data linkage process, including the number of individuals with linked data at each stage.	Page 6, materials & methods, paragraph 2<br><br>Figure 1<br><br>Page 6, materials & methods, paragraph 3<br><br>N/A
Variables	7	Clearly define all outcomes, exposures, predictors, potential confounders, and effect modifiers. Give diagnostic criteria, if applicable.	Page 7, materials & methods, paragraph 1<br><br>Page 9, materials & methods, paragraphs 2 and 3	RECORD 7.1: A complete list of codes and algorithms used to classify exposures, outcomes, confounders, and effect modifiers should be provided. If these cannot be reported, an explanation should be provided.	Page 17, data sharing
Data sources/ measurement	8	For each variable of interest, give sources of data and details of methods of assessment (measurement).	Page 6, materials & methods, paragraph 2		

For peer review only

		Describe comparability of assessment methods if there is more than one group	Page 8, materials & methods, paragraph 4<br><br>Page 9, materials & methods, paragraphs 2 and 3		
Bias	9	Describe any efforts to address potential sources of bias	Page 9, materials & methods, paragraph 3		
Study size	10	Explain how the study size was arrived at	Page 9, materials & methods, paragraph 2		
Quantitative variables	11	Explain how quantitative variables were handled in the analyses. If applicable, describe which groupings were chosen, and why	Page 9, materials & methods, paragraphs 2 and 3		
Statistical methods	12	(a) Describe all statistical methods, including those used to control for confounding<br>(b) Describe any methods used to examine subgroups and interactions<br>(c) Explain how missing data were addressed<br>(d) *Cohort study* - If applicable, explain how loss to follow-up was addressed<br>*Case-control study* - If applicable, explain how matching of cases and controls was addressed<br>*Cross-sectional study* - If applicable, describe analytical methods taking account of sampling strategy	(a) Page 7, materials & methods, paragraph 2<br><br>Page 9, materials & methods, paragraphs 2 and 3<br><br>(b) N/A<br><br>(c) Page 9, materials & methods, paragraph 3<br><br>(d) N/A<br><br>(e) N/A		

		(e) Describe any sensitivity analyses			
Data access and cleaning methods		..		RECORD 12.1: Authors should describe the extent to which the investigators had access to the database population used to create the study population.<br><br>RECORD 12.2: Authors should provide information on the data cleaning methods used in the study.	Page 6, materials & methods, paragraphs 1 and 2<br><br>Figure 2 (text pre-processing)<br><br>Page 9, materials & methods, paragraph 2
Linkage		..		RECORD 12.3: State whether the study included person-level, institutional-level, or other data linkage across two or more databases. The methods of linkage and methods of linkage quality evaluation should be provided.	N/A
**Results**					
Participants	13	(a) Report the numbers of individuals at each stage of the study (*e.g.*, numbers potentially eligible, examined for eligibility, confirmed eligible, included in the study, completing follow-up, and analysed)<br>(b) Give reasons for non-participation at each stage.<br>(c) Consider use of a flow diagram	(a) Figure 1<br><br>Page 10, results, paragraph 4<br><br>Table 1<br><br>(b) Figure 1<br><br>(c) Figure 3 (flow diagram)	RECORD 13.1: Describe in detail the selection of the persons included in the study (*i.e.*, study population selection) including filtering based on data quality, data availability and linkage. The selection of included persons can be described in the text and/or by means of the study flow diagram.	Figure 1<br><br>Figure 3
Descriptive data	14	(a) Give characteristics of study participants (*e.g.*, demographic, clinical, social) and information on exposures and potential confounders	(a) Page 10, results, paragraph 5<br><br>Table 1		

For peer review only

		(b) Indicate the number of participants with missing data for each variable of interest<br>(c) *Cohort study* - summarise follow-up time (*e.g.*, average and total amount)	(b) Figure 3<br><br>Page 11, results, paragraph 1<br><br>Table 2		
Outcome data	15	*Cohort study* - Report numbers of outcome events or summary measures over time<br>*Case-control study* - Report numbers in each exposure category, or summary measures of exposure<br>*Cross-sectional study* - Report numbers of outcome events or summary measures	Page 11, results, paragraphs 1, 2 and 3		
Main results	16	(a) Give unadjusted estimates and, if applicable, confounder-adjusted estimates and their precision (e.g., 95% confidence interval). Make clear which confounders were adjusted for and why they were included<br>(b) Report category boundaries when continuous variables were categorized<br>(c) If relevant, consider translating estimates of relative risk into absolute risk for a meaningful time period	(a) Page 10, results, paragraph 3<br><br>Page 11, results, paragraph 5<br><br>Table 2<br><br>(b) Table 2<br><br>(c) N/A		
Other analyses	17	Report other analyses done—e.g., analyses of subgroups and interactions, and sensitivity analyses	N/A		
**Discussion**					
Key results	18	Summarise key results with reference to study objectives	Page 13, discussion, paragraph 1		

For peer review only

Limitations	19	Discuss limitations of the study, taking into account sources of potential bias or imprecision. Discuss both direction and magnitude of any potential bias	Page 13, discussion, paragraph 1<br><br>Page 14, discussion, paragraph 2	RECORD 19.1: Discuss the implications of using data that were not created or collected to answer the specific research question(s). Include discussion of misclassification bias, unmeasured confounding, missing data, and changing eligibility over time, as they pertain to the study being reported.	Page 14, discussion, paragraph 2
Interpretation	20	Give a cautious overall interpretation of results considering objectives, limitations, multiplicity of analyses, results from similar studies, and other relevant evidence	Page 13, discussion, paragraphs 1 and 2		
Generalisability	21	Discuss the generalisability (external validity) of the study results	Page 14, discussion, paragraph 2		
**Other Information**					
Funding	22	Give the source of funding and the role of the funders for the present study and, if applicable, for the original study on which the present article is based	Page 15, funding		
Accessibility of protocol, raw data, and programming code		..		RECORD 22.1: Authors should provide information on how to access any supplemental information such as the study protocol, raw data, or programming code.	Supplementary materials 1, 2 and 3<br><br>Page 17, data sharing

*Reference: Benchimol EI, Smeeth L, Guttmann A, Harron K, Moher D, Petersen I, Sørensen HT, von Elm E, Langan SM, the RECORD Working Committee. The REporting of studies Conducted using Observational Routinely-collected health Data (RECORD) Statement. *PLoS Medicine* 2015; in press.

*Checklist is protected under Creative Commons Attribution (CC BY) license.

BMJ Open

Text-mining occupations from the mental health electronic health record: a natural language processing approach using records from the Clinical Record Interactive Search (CRIS) platform in south London, UK.

Journal:	*BMJ Open*
Manuscript ID	bmjopen-2020-042274.R2
Article Type:	Original research
Date Submitted by the Author:	06-Nov-2020
Complete List of Authors:	Chilman, Natasha; King's College London, Department of Psychological Medicine; Insitute of Psychiatry, Psychology & Neuroscience<br>Song, Xingyi; The University of Sheffield<br>Roberts, Angus; King's College London, Institute of Psychiatry, Psychology and Neuroscience<br>Tolani, Esther; King's College London, Institute of Psychiatry, Psychology & Neuroscience<br>Stewart, Robert; King's College London, Institute of Psychiatry; South London and Maudsley NHS Foundation Trust<br>Chui, Zoe; King's College London, Institute of Psychiatry, Psychology & Neuroscience<br>Birnie, Karen; King's College London; King's College Hospital NHS Foundation Trust<br>Harber-Aschan, Lisa; King's College London<br>Gazard, Billy; King's College London<br>Chandran, David; South London and Maudsley NHS Foundation Trust, NIHR Biomedical Research Centre<br>Sanyal, Jyoti; South London and Maudsley NHS Foundation Trust, NIHR Biomedical Research Centre<br>HATCH, STEPHANI; King's College London, Institute of Psychiatry, Psychology and Neuroscience<br>Kolliakou, Anna; King's College London, Institute of Psychiatry, Psychology & Neuroscience<br>Das-Munshi, Jayati; King's College London, Department of Psychological Medicine, Institute of Psychiatry, Psychology & Neuroscience
**Primary Subject Heading**:	Health informatics
Secondary Subject Heading:	Epidemiology, Mental health, Occupational and environmental medicine
Keywords:	MENTAL HEALTH, Health informatics < BIOTECHNOLOGY & BIOINFORMATICS, EPIDEMIOLOGY, Adult psychiatry < PSYCHIATRY

I, the Submitting Author has the right to grant and does grant on behalf of all authors of the Work (as defined in the below author licence), an exclusive licence and/or a non-exclusive licence for contributions from authors who are: i) UK Crown employees; ii) where BMJ has agreed a CC-BY licence shall apply, and/or iii) in accordance with the terms applicable for US Federal Government officers or employees acting as part of their official duties; on a worldwide, perpetual, irrevocable, royalty-free basis to BMJ Publishing Group Ltd ("BMJ") its licensees and where the relevant Journal is co-owned by BMJ to the co-owners of the Journal, to publish the Work in this journal and any other BMJ products and to exploit all rights, as set out in our licence.

The Submitting Author accepts and understands that any supply made under these terms is made by BMJ to the Submitting Author unless you are acting as an employee on behalf of your employer or a postgraduate student of an affiliated institution which is paying any applicable article publishing charge ("APC") for Open Access articles. Where the Submitting Author wishes to make the Work available on an Open Access basis (and intends to pay the relevant APC), the terms of reuse of such Open Access shall be governed by a Creative Commons licence – details of these licences and which Creative Commons licence will apply to this Work are set out in our licence referred to above.

Other than as permitted in any relevant BMJ Author's Self Archiving Policies, I confirm this Work has not been accepted for publication elsewhere, is not being considered for publication elsewhere and does not duplicate material already published. I confirm all authors consent to publication of this Work and authorise the granting of this licence.

For peer review only

Text-mining occupations from the mental health electronic health record: a natural language processing approach using records from the Clinical Record Interactive Search (CRIS) platform in south London, UK.

Natasha Chilman[1*], Xingyi Song[3], Angus Roberts[1], Esther Tolani[1], Robert Stewart[1,2] Zoe Chui[1], Karen Birnie[1, 5], Lisa Harber-Aschan[1], Billy Gazard[1], David Chandran[2], Jyoti Sanyal[2], Stephani L Hatch[1,4], Anna Kolliakou[1, **], Jayati Das-Munshi[1,2, 4**]

***Joint senior author*

***Corresponding author contact information:**

Natasha Chilman, East Wing 3.16, Department of Psychological Medicine, Institute of Psychiatry, Psychology & Neuroscience, Kings College London, DeCrespigny Park, London, SE5 8AF. Telephone: +44 796968 8554. Email: natasha.chilman@kcl.ac.uk

Author details:

[1] Institute of Psychiatry, Psychology and Neuroscience, King's College London, London, UK. [2] South London and Maudsley NHS Foundation Trust, London, UK. [3] University of Sheffield, Sheffield, UK. [4]Economic and Social Research Council (ESRC) Centre for Society and Mental Health, King's College London, UK. [5]King's College Hospital NHS Trust, London, UK.

ORCID identifiers:

Natasha Chilman 0000-0002-9661-5098

Xingyi Song 0000-0002-4188-6974

Angus Roberts 0000-0002-4570-9801

Esther Tolani 0000-0002-7415-0859

Robert Stewart 0000-0002-4435-6397

Zoe Chui 0000-0001-6844-6779

Karen Birnie 0000-0003-4123-1676

Lisa Harber-Aschan 0000-0002-6464-485

Billy Gazard 0000-0002-7562-539

For peer review only - http://bmjopen.bmj.com/site/about/guidelines.xhtml

For peer review only

David Chandran 0000-0002-0123-666X

Jyoti Sanyal – N/A

Stephani Hatch 0000-0001-9103-2427

Anna Kolliakou 0000-0003-1234-4129

Jayati Das-Munshi 0000-0002-3913-6859

Word Count

4,223

Keywords

Mental Health

Health informatics

Epidemiology

Adult psychiatry

For peer review only

ABSTRACT

Objectives

We set out to develop, evaluate, and implement a novel application using natural language processing to text-mine occupations from the free-text of psychiatric clinical notes.

Design

Development and validation of a natural language processing application using General Architecture for Text Engineering (GATE) software to extract occupations from de-identified clinical records.

Setting & Participants

Electronic health records from a large secondary mental health provider in south London, accessed through the Clinical Record Interactive Search (CRIS) platform. The text-mining application was run over the free-text fields in the electronic health records of 341,720 patients (all aged ≥16).

Outcomes

Precision and recall estimates of the application performance; occupation retrieval using the application compared to structured fields; most common patient occupations; and analysis of key sociodemographic and clinical indicators for occupation recording.

Results

Using the structured fields alone, only 14% of patients had occupation recorded. By implementing the text-mining application in addition to the structured fields, occupations were identified in 57% of patients. The application performed on gold-standard human-annotated clinical text at a precision level of 0.79 and recall level of 0.77. The most common patient occupations recorded were ‘student’, and ‘unemployed’. Patients with more service contact were more likely to have an occupation recorded, as were patients of a male gender, older age, and those living in areas of lower deprivation.

Conclusion

This is the first time a natural language processing application has been used to successfully derive patient-level occupations from the free-text of electronic mental health records, performing with good levels of precision and recall, and applied at scale. This may be used to inform clinical studies relating to the broader social determinants of health using electronic health records.

For peer review only - http://bmjopen.bmj.com/site/about/guidelines.xhtml

ARTICLE SUMMARY

Strengths and Limitations

- The application was developed on a sizeable corpus of training and test data from a large routine dataset, which was applied at scale over the record, providing us with insights into the occupations of patients using secondary mental health services.
- The application was thoroughly evaluated using gold-standard and cross-checking strategies.
- The application was developed and tested in a single site electronic health record system in the UK – the application will require validation on other similar systems before use with them.
- The application does not identify the temporality of occupations; it is unclear whether the extracted occupations are currently or previously held by the patient.
- Health and social care occupations were prevented from being assigned to the patient as these could not be ascertained with confidence, therefore the application cannot yet identify where a patient holds a health/social care occupation.

For peer review only

INTRODUCTION

Occupation and mental illness are highly interrelated. There are long-standing concerns that unemployment rates are considerably higher for people with mental illness [1, 2], and work participation has been described as among the most important factors for recovery by clinicians and service users alike [3, 4]. People with mental illnesses may also undertake precarious, poorly paid work which could have further negative impacts on mental health [5]. Moreover, occupation is a fundamental individual-level indicator of socio-economic position as it is predictive of material resources and is indicative of wider class interactions [6]. Recent systematic reviews have called for large and detailed longitudinal studies to investigate predictors of occupational functioning, and to examine how and when occupation is associated with clinical outcomes in mental health cohorts, as this is currently poorly understood [7, 8].

Research using electronic health records (EHRs) allows for the large-scale collection of sociodemographic and clinical information which would otherwise be logistically challenging to collect using traditional epidemiological approaches [9]. However, EHR research has major limitations including that information relating to occupation is either not recorded routinely or is poorly captured within standard EHR systems [10]. As there are no existing methods, to our knowledge, to reliably extract occupations from the psychiatric EHR, this is a problematic barrier for desirable research where occupation is an indicator of socioeconomic status and in research examining the relationships between occupation, mental illness and recovery.

Patient information can be recorded in the structured fields of the EHR, where the clinician records categorical or numerical data. In many psychiatric EHR systems, patient information is recorded in narrative text sections of the record, known as the 'free-text' fields, for example in notes describing patient contact [11]. Information recorded in this way is harder to extract. Clinicians may only record the patient's occupation in such free-text fields and not the structured fields, making it more complicated, time consuming and labour intensive to identify the patient's occupation [10]. Natural language processing (NLP) methods have the potential to overcome this obstacle by applying algorithms to extract relevant textual information. NLP methods have previously been used successfully for text-mining from mental health EHRs, for example to identify smoking status and symptoms of severe mental illness [12-16], and other types of clinical records [17, 18]. NLP methods are also being applied in large-scale industrial and occupational research [19-21]. This paper traces the development of a novel application using NLP methods to extract patient occupations from the free-text of EHRs from a large mental health Trust in south London, UK. We then provide profile information on the most frequently extracted occupations for patients using secondary mental health services, and clinical and sociodemographic factors associated with recorded occupation data compared to missing occupation data.

For peer review only - http://bmjopen.bmj.com/site/about/guidelines.xhtml

MATERIALS AND METHODS

Setting

Data for the development of the application were obtained from the South London and Maudsley (SLaM) Biomedical Research Centre (BRC) Case Register: a repository of de-identified clinical data from the EHRs of individuals receiving care from SLaM secondary mental health services. SLaM covers a socially and ethnically diverse inner-city area of approximately 1.3 million people [22]. The register contains over 350,000 de-identified patient records which are available for research purposes through the Clinical Record Interactive Search (CRIS) platform. CRIS was developed at SLaM in 2008 and similar resources have subsequently been implemented at several other mental health Trusts in the UK. The present application was developed over the years 2017-2019 and was implemented in January 2020.

Datasets

Figure 1 describes how the CRIS-derived dataset was used for cycles of application development and evaluation, and summarises the key steps taken. Age restrictions were implemented throughout document selection: free-text documents were only extracted where the patient was aged 16 and above at time of document extraction. There were no date restrictions. Free-text documents were retrieved from several different sections in this EHR, for example sections for clinical risk assessments and separate sections for discharge summaries. Further detail on the types of documents used at each stage of application development can be found in supplementary file 1.

Developing, Evaluating and Implementing the Application

Manually annotating occupation in the free-text (Figure 1, steps 1-3)

Personal history sections of psychiatric assessments typically describe the patient's occupation, as well as education and family history. Personal history sections of documents were extracted from the free-text fields of records at the document level using an NLP application (precision=0.78, recall=0.88) developed by DC (N=67,383). Typically these extracts were derived from documents of the 'attachments' type, which is a word-processed document such as a letter to or from the patient's primary care physician; and 'events', which are short pieces of text used to record some detail of a clinical encounter.

Occupations were identified in personal history documents by an interdisciplinary team of trained researchers, including clinicians, bioinformaticians and mental health researchers. In common with the NLP community, we refer to this task of marking mentions of occupation text as annotation. A set of occupation annotation guidelines were developed through an iterative process of manual annotation practice, team discussions and agreed annotation rulemaking (supplementary file 2). These guidelines

For peer review only - http://bmjopen.bmj.com/site/about/guidelines.xhtml

specified when and how an occupation should be identified, annotated and extracted from the text. An occupation annotation was defined as having two parts. Firstly, the *occupation* itself was annotated. This could be an occupation title, for example a 'builder'; or an occupation description, for example 'construction'. Secondly, the occupation *relation* was specified: who the occupation belongs to, for example the patient or their family member. Temporality, including when or how long a patient has held an occupation, was not annotated as the text often did not state this consistently. In total, 600 personal history documents were manually annotated to practice annotating occupation from text and develop the annotation guidelines (ET, AK, SM, KB, ZC, AR). Once the guidelines were developed, a set of 1000 personal history documents were manually annotated on the General Architecture for Text Engineering (GATE) platform [23] using the guidelines to create a gold-standard, where 200 were double annotated to evaluate inter-annotator reliability.

Application development (Figure 1, step 4)

Out of the 1000 gold-standard annotated personal history documents, 334 documents were reserved for application development. The application was developed by XS on the GATE platform [23], a widely used NLP framework with over 40 thousand downloads per version and a history of use in the UK national health service, amongst other sectors [17]. The application was trained on 257 of the gold-standard annotated documents. To check the performance of the application throughout development, precision and recall metrics were estimated using a customized performance tool developed by XS on GATE on a validation set of 77 gold-standard annotated documents, with a total of 405 occupation annotations. Precision was the proportion of occupations correctly annotated, to all occupations annotated (whether correct or incorrect). Recall was the proportion of occupations correctly annotated, to all occupations that could have been correctly annotated. The application outputs were manually checked by the Clinical Informatics Interface and Network Lead at the NIHR BRC (AK). Any problems identified were addressed in each version of the application. An iterative process of application development, training, evaluation of performance using GATE and manual checks was repeated 10 times, at which point the application reached a good level of performance on the validation set.

Machine-learning approach testing (Figure 1, steps 5-6)

Two early versions of the application were developed for testing over unannotated documents in the CRIS case register: one version used combined machine-learning and rule-based approaches, and the second version used rule-based approaches only. This was due to a concern that the application had therein been developed on limited training data, and the trained model may not generalise well on the free-text other than personal history documents, which could lead to a loss in precision when implemented over the EHR. Specifically, the machine-learning approaches involved a trained conditional random field classifier to identify occupation mentions in the text, and a support-vector machine-based classifier to identify the occupation relation. Figure 2 illustrates how the machine-

For peer review only - http://bmjopen.bmj.com/site/about/guidelines.xhtml

learning and rule-based approaches were used in combination; this is described in further technical detail in supplementary file 3.

Two researchers (NC, AK) manually calculated precision performance for both versions of the application on 100 personal history documents (in domain testing data) and 100 other free-text document types (out domain test data) which had at least one occupation extraction and were previously unseen by the application in development. Whilst both application versions performed well when text-mining occupations from these test sets (precision ≥0.79, further detail in supplementary file 3), the application with machine-learning approaches performed at the highest level of precision when assigning the occupation relation - i.e. who the occupation was held by. The research team concluded from this testing phase that the application with combined machine-learning and rule-based approaches was most appropriate, as this pipeline performed best at assigning the occupation relation.

The healthcare occupation filter (Figure 1, step 7)

The evaluation of the application performance over CRIS documents revealed that the most common false positives were extractions where the healthcare professional involved in the patient's care was incorrectly annotated as the patient's occupation (96% of annotations manually checked were health/social care occupations). To deal with this issue, health and social care occupations were added to a filter. The application then implemented a rules-based step where the filtered healthcare occupations were prevented from being annotated as belonging to the patient. Occupations added to this filter included variations on terms for psychiatrists and doctors, therapists, nurses, and social workers, following the checking of 2,390 documents to confirm that these were common false positives.

Application implementation and testing (Figure 1, steps 8-10)

The final version of the text-mining application with the healthcare filter applied was run over 10 free-text fields, including those where personal history sections were found, in the records of all patients on the CRIS case register aged 16 and above. The fields included sections of the record such as discharge summaries, attachments, events and risk assessments (more detail in supplementary file 1). The application was evaluated on a total of 866 documents: 666 gold-standard annotated personal history documents (test corpus 1), and 200 previously unannotated random personal history documents from the CRIS dataset at the time of the application run (test corpus 2). Test corpus 1 was evaluated on GATE, and test corpus 2 was manually checked for occupations and then cross-referenced with the application output. The performance metrics considered the precision and recall level for the annotations made by the application, where both the occupation annotation and the relation classification needed to be accurate to be considered a 'true positive'. It was not feasible in this study to randomly select non-personal-history documents for evaluation as patient occupations were rarely mentioned in the record compared to other information (e.g. medication). As the application extracted

For peer review only - http://bmjopen.bmj.com/site/about/guidelines.xhtml

an annotation entitled ‘other’, 200 of these annotations were manually checked for precision to further investigate these instances where the application was unable to assign an occupation title.

The EHR in the present study contains a structured field to record occupation: the ‘Employment-ID’. This was explored on the CRIS platform using SQL queries. The proportion of completed ‘Employment-IDs’ from the records of all patients over the age of 16 in January 2020 was extracted. The text-mining application was simultaneously run over clinical records through CRIS, and the extracted patient occupations were converted into an SQL table. Sociodemographic, clinical and service contact data was also extracted from the structured fields of records using SQL queries. Data was exported to and analysed in STATA-15 to examine predictors of occupational data extraction using logistic regression models. This included the patient’s age at time of occupation extraction, gender, marital status, ethnicity, index of multiple deprivation (IMD) score and primary diagnosis. Indicators of service contact included number of events in the record, number of face-to-face events in the record, number of spaces in the free-text fields of the record (as a proxy for word count), number of active days under SLaM services, and number of inpatient bed days. These variables were transformed into categories, for example IMD scores were categorised into quartiles of local neighbourhood deprivation. Where data was missing for the extracted variables, this was coded as a ‘Not Known’ category for each variable.

Logistic regression models examined crude associations between the sociodemographic, clinical, and service contact variables (predictors) and the recording of at least one patient-occupation (outcome) from either the structured or free-text fields. The null hypothesis was that none of the predictors would be associated with likelihood of occupation recording. Firstly, models were adjusted for amount of contact the patient had with services. Fully adjusted models accounted for all other sociodemographic and clinical variables. Across all models, likelihood ratio tests were conducted to test the overall association between the variable and occupation recording. The aim of this analysis was to ascertain the characteristics of patients who had occupation recorded in their health record.

Patient and Public Involvement

This study proposal was reviewed and approved by the patient-led CRIS oversight committee prior to the commencement of the project. No other consultations were made with patients or the public during the process of the study.

For peer review only - http://bmjopen.bmj.com/site/about/guidelines.xhtml

RESULTS

Annotating Occupation

When double-annotating 200 personal history documents, two annotators reached a Cohen's kappa agreement [24] of 0.77 for occupation title annotations and 0.72 for occupation relation annotations. Disagreements between annotators included instances where sentences posed unclear or vague references to occupation: for example, in the sentence, "she did several things, such as cleaning, cooking", it was not clear whether these were domestic tasks or occupation-descriptions, demonstrating the complexity of annotating occupation from text. Nonetheless, the Cohen's kappa agreement suggested that occupation could be annotated reasonably consistently across annotators using the annotation guidelines.

Application Development

The application reached a precision level of 0.88 and a recall level of 0.90 on the validation set of documents (N=77). The developed application process with combined rule-based and machine learning approaches is described in Figure 2.

Application Performance

When applied to the gold-standard annotated personal history documents (test corpus 1) on GATE, the application performed at a precision level of 0.79 and a recall level of 0.77. Two-hundred personal history documents were manually checked for occupations and then cross-referenced with the application output (test corpus 2): when considering patient-occupations only, the application reached a precision level of 0.77 and recall level of 0.79. An extraction of 'other' as an occupational category was excluded from subsequent analysis, as the check of 200 annotations showed that this annotation only reached a precision level of 0.23 and often referenced job-seeking or non-work behaviours, for example 'working on his anxiety'.

Application Implementation

Figure 3 shows the study population selection process for the implementation of the application over the CRIS case register, leading to an overall sample size of 341,720 patients.

Descriptives

The demographics of the study population at the time of occupation extraction is described in Table 1, as well as patient diagnostic categories and two indicators of the amount of service contact the patient has had: the number of 'events' entries added to the EHR, and number of inpatient bed days. The three other extracted indicators for service contact (number of 'face-to-face events', total active days under SLaM mental health services, and number of spaces in the text in the record) were excluded from analysis due to collinearity with the 'events' variable.

Occupation Extractions

For peer review only - http://bmjopen.bmj.com/site/about/guidelines.xhtml

The structured field for employment was populated for 46,705 (13.7%) patients. Prior to the implementation of the healthcare filter, 81.5% patients had at least one patient-occupation extraction. When using the final version of application to extract occupations from the free-text fields with the healthcare filter applied, this recalled at least one patient-related occupation for 184,521 patients (54.0%). By combining structured field and extracted occupations, patient-related occupations were retrieved for 193,616 patients (56.7%) over the dataset.

The structured field for occupation included 13 categories for occupational status, for example ‘unemployed’ or ‘paid employment’. In contrast, the text-mining application retrieved 72,955 different patient-related occupation types. In total, there were 3,957,959 patient-related occupation extractions. Multiple occupation types were often extracted per patient (median=4, inter-quartile-range=6).

The top 5 extracted occupations across the total sample of 341,720 patients were: student (n=98,719, 28.9%), unemployed (n=97,809, 28.6%), carer (n=61,893, 18.1%), self-employed (n=36,506, 10.8%) and retired (n=33,518, 9.8%). The less frequent extractions tended to be more specific occupation types, for example, ‘retail worker’, and ‘banker’. The application also extracted ‘undocumented’ ways of making money, including ‘drug dealer’ and ‘sex worker’.

Associations with Occupation Recording

Patients were split into two binary categories: those who had an occupation recorded either in the structured field or free-text (n=193,616, 56.7%), and patients who did not have occupation recorded, i.e. missing occupational data (n=148,104, 43.3%). Logistic regressions were used to examine sociodemographic, clinical, and service contact associations with recorded occupations (Table 2).

Across all models, all predictors were strongly associated with a recording of occupation even after fully adjusting for all other variables (likelihood ratio tests $p<.0001$). When key sociodemographic data was missing from the record, the odds of occupational data being recorded decreased: for example, where the marital status of the patient was ‘Not Known’, the fully-adjusted odds ratio for a recording of an occupation was 0.49 (95% CI 0.47-0.50) compared to patients who were recorded as married/in a civil partnership/cohabiting. Female patients were significantly less likely to have an occupation extracted compared to male patients, and older patients were most likely to have occupational data recorded compared to the youngest patients. Compared to patients of White British ethnicity, patients of Irish, Black Caribbean, or Black African ethnicity were more likely to have an occupation recorded; whilst Indian, Pakistani, Chinese, Mixed Race or patients recorded as being from ‘other’ Asian or ethnic groups were less likely to have occupation recorded. The odds of having occupation recorded were significantly lower for patients who were living in the most deprived local areas compared to the most affluent areas. Generally, patients with a primary diagnosis of an affective disorder had a higher odds of an occupation extraction than patients with other diagnoses, including organic disorders. In the crude logistic regression models, patients diagnosed with schizophrenia, schizotypal or delusional disorders were more likely to have occupation

For peer review only - http://bmjopen.bmj.com/site/about/guidelines.xhtml

extracted (OR 1.61, P5% CI 1.54-1.68). However, once adjusting for amount of contact with services, these patients were significantly less likely to have occupation extracted compared to patients with affective disorders (adjusted OR 0.87, 95% CI 0.83-0.91).

For peer review only

For peer review only - http://bmjopen.bmj.com/site/about/guidelines.xhtml

DISCUSSION

Annotating and extracting occupation from the free-text in clinical records is a challenging task. We have developed a tool to text-mine patient occupations with a good degree of confidence from a mental health EHR, and applied this at scale over a large EHR in south London. An important finding was that we could retrieve over double the number of patient occupations using text-mining methodology than when using pre-existing structured fields alone. We could also access a much wider diversity of occupation types: this further detail on occupations held by patients opens up the possibility for the translation of occupations onto social class schema, which would not have been possible with the limited structured field categories. The most prevalent patient-occupations were 'student' and 'unemployed'. There were differences between patients who had occupation recorded and patients where occupation data remained missing: patients with occupations recorded were more likely to be of an older age, male, divorced/separated, living in areas of lower deprivation, and had more contact with mental health services. Across ethnic minority groups, there were mixed findings relating to the recording of occupation. Compared to White British patients, Irish, Black Caribbean and Black African patients were slightly more likely to have a recording of occupation, whereas all other ethnic minority groups were less likely to have a recording. Although it is possible that some of the demographic associations with the recording of occupation in the case notes were impacted upon by residual confounding in adjusted models, these findings may also indicate disparities relating to how occupations are assessed and recorded in the clinical record and should be explored in future work, particularly given the strong correlation of employment with recovery, within the context of mental disorders.

This study broadly supports the work of other studies which indicate that clinicians mostly describe occupation in the free-text of EHR systems, when these are available, rather than structured fields [10]. This study is the first of its kind to text-mine patient occupations from a mental healthcare EHR. There have been several previous efforts to extract patient occupations from other healthcare free-text notes. Occupations have been text-mined from general medical clinical text; however, in these studies the algorithms reached low levels of performance, largely due to a lack of training data [25, 26]. Dehghan and colleagues' text-mined occupation from the clinical records of cancer patients in the UK, reaching similar precision and recall levels to the present study [27]. However, none of these applications distinguished between text-mining occupations belonging to the patient and other relations, had the scope of applying and testing the text-mining methodology at scale across the EHR, or examined associations with extracted versus missing occupational data. The present application therefore represents significant progress in our ability to text-mine patient occupations from the EHR and furthers our understanding of what this may mean in practice.

We found that text-mining greatly increased our retrieval of patient-occupations in this psychiatry EHR database. Psychiatric notes may be more detailed than other types of healthcare text (for example, in

For peer review only - http://bmjopen.bmj.com/site/about/guidelines.xhtml

general medicine) when describing the patient's occupation, as this often forms part of psychiatric history taking and assessment. We found that a sizeable proportion of patients over CRIS have at some point been a student or unemployed. A separate NLP application being developed using CRIS data (by author JS) will be able to interrogate this student group further by extracting the patient's level of educational attainment, which will complement the present application. There is also scope to explore older groups of patients who are students but are also working using this methodology. Our finding that unemployment was a dominant occupational category is consistent with previous research, in that unemployment levels are elevated particularly for those with severe mental illnesses compared to the general population [1, 2]. It may also be the case that some patients in this group are formally unemployed but are working in more informal, undocumented ways to make money. This application identified some informal occupations, which is an interesting avenue for further research.

One limitation of our approach is that we could not distinguish the temporality of occupations – whether they were currently or previously held by the patient. Whilst developing the annotation guidelines, we found that the text was unlikely to be sufficient to assess temporality, as it was often not explicitly stated when the patient started or left an occupation, or how long they have held a position for. Multiple occupations were often extracted for a single patient, adding to the complexity. Whilst there is work ongoing to use NLP to detect temporality in psychiatric healthcare text [28], this remains a challenge and is a potential avenue for further work that is beyond the scope of this paper. As this application was developed at a single site in the UK, the generalisability of the application may be reduced, firstly to English language and secondly to this catchment area. As it was not possible to assign health and social care occupations to patients with reasonable confidence, we will also be missing patients who hold these occupations; however, we are planning further work to develop this aspect of the application further. Notwithstanding these limitations, this application was developed through an extensive process of training and testing using a large corpus leading to the application of text-mining algorithms for occupation at scale. This methodology is already revealing the kinds of occupations held by patients using secondary mental health services.

The development of this methodology has numerous implications. Firstly, this application will be valuable in allowing researchers to examine relationships between occupation and health in large psychiatric case registers. For example, work is currently underway using this application to investigate predictors of unemployment in a cohort of patients with severe mental illness [29]. As CRIS-like systems are in use over several sites in the UK, there is the scope to test and implement this application in other mental healthcare providers using similar EHR platforms. This application could also have potential practical implications including identifying unemployed patients to target interventions such as Individual Placement and Support (IPS) and retrieving occupational distributions for audits and organisational monitoring in NHS mental health Trusts. Lastly, this application may have implications

For peer review only - http://bmjopen.bmj.com/site/about/guidelines.xhtml

beyond mental health research and text, notably in research on industrial injuries, although this requires further testing.

There is room for further progress in this application as the NLP field further develops, including identifying the temporality of occupations and improving relation classification for health and social care occupations. We plan to develop methodology to ascertain the occupational social class of patients, using the large diversity of occupations extracted, to further inform health inequalities research specific to mental health. Future studies implementing this application in other CRIS systems may be able to investigate the transferability of the application to other NHS sites in the UK that serve different patient populations. Overall, we hope that this approach will prove useful in addressing our understanding of the interactions between occupation and health in those with mental illness.

For peer review only

ORIGINAL PROTOCOL

N/A

FUNDING STATEMENT

This work was supported by the Clinical Record Interactive Search system which is funded by the National Institute for Health Research (NIHR) Biomedical Research Centre at South London and Maudsley National Health Service (NHS) Foundation Trust and King's College London and a joint infrastructure grant from Guy's and St Thomas' Charity and the Maudsley Charity. Specific application development was supported by the Health Foundation, in a grant held by JD.

JD is funded by the Health Foundation working together with the Academy of Medical Sciences, for a Clinician Scientist Fellowship and by the ESRC in relation to the SEP-MD study (ES/S002715/1) and part supported by the ESRC Centre for Society and Mental Health at King's College London (ESRC Reference: ES/S012567/1).

RS is part-funded by: i) the National Institute for Health Research (NIHR) Biomedical Research Centre at the South London and Maudsley NHS Foundation Trust and King's College London; ii) a Medical Research Council (MRC) Mental Health Data Pathfinder Award to King's College London; iii) an NIHR Senior Investigator Award; iv) the National Institute for Health Research (NIHR) Applied Research Collaboration South London (NIHR ARC South London) at King's College Hospital NHS Foundation Trust.

SH is part-funded by the National Institute for Health Research (NIHR) Biomedical Research Centre at the South London and Maudsley NHS Foundation Trust and King's College London; ESRC Centre for Society and Mental Health at King's College London (ESRC Reference: ES/S012567/1); and the Wellcome Trust (203380Z16Z).

The funders had no role in the study design, data collection and analysis, decision to publish, or preparation of the manuscript. The views expressed are those of the author(s) and not necessarily those of the NHS, the NIHR, the MRC, the Department of Health, the ESRC or King's College London.

COMPETING INTERESTS STATEMENT

All authors have confirmed that they have no competing interests to declare.

CHECKLIST/FLOW DIAGRAM FOR REPORTING STATEMENT

For peer review only - http://bmjopen.bmj.com/site/about/guidelines.xhtml

The RECORD Statement checklist is attached to this submission.

ETHICS

The SLaM Case Register has been approved as a source of de-identified data for secondary analyses (Oxford Research Ethics Committee C, reference 18/SC/0372).

DATA SHARING STATEMENT

We are unable to place test data in the public domain because these comprise patient information, but document IDs used in application development and testing have been archived and researchers may apply for approval to access these or other CRIS data. This application is also being put into production for researchers to use in the Biomedical Research Centre. More information can be found at http://brc.slam.nhs.uk/about/core-facilities/cris.

AUTHOR CONTRIBUTIONS

The study was conceived by JD, AK, RS, AR, SH, BG and LHA. Personal history sections of documents were extracted using an application developed by DC. Manual annotations to develop the annotation guidelines and produce the test and training data were conducted by AK, AR, ET, ZC and KB, and SM (acknowledgements). The application was developed by XS, with feedback from AK, NC, JD, RS, AR, and SH. The application was implemented over the EHR by DC and JS. The application was evaluated by AK and NC. The missing data analysis was conducted by NC and JD. The paper draft was led by NC, JD and AK; and was critically reviewed and edited by all authors (AK, XS, AR, ET, RS, ZC, KB, DC, JS, BG, LHA, SH).

ACKNOLWEDGEMENTS

We appreciated the technical support from informatics personnel in the NIHR Maudsley Biomedical Research Centre and the University of Sheffield. We would also like to thank Shirlee MacCrimmon for her assistance with annotations during the annotation guideline development process.

REFERENCES

For peer review only - http://bmjopen.bmj.com/site/about/guidelines.xhtml

REFERENCES

1. Luciano, A. and E. Meara, *Employment status of people with mental illness: national survey data from 2009 and 2010.* Psychiatric Services, 2014. **65**(10): p. 1201-1209.
2. Marwaha, S., et al., *Rates and correlates of employment in people with schizophrenia in the UK, France and Germany.* The British Journal of Psychiatry, 2007. **191**(1): p. 30-37.
3. Dunn, E.C., N.J. Wewiorski, and E.S. Rogers, *The meaning and importance of employment to people in recovery from serious mental illness: results of a qualitative study.* Psychiatric rehabilitation journal, 2008. **32**(1): p. 59.
4. Marwaha, S. and S. Johnson, *Schizophrenia and employment.* Social psychiatry and psychiatric epidemiology, 2004. **39**(5): p. 337-349.
5. Moscone, F., E. Tosetti, and G. Vittadini, *The impact of precarious employment on mental health: The case of Italy.* Social Science & Medicine, 2016. **158**: p. 86-95.
6. Connelly, R., V. Gayle, and P.S. Lambert, *A review of occupation-based social classifications for social survey research.* Methodological Innovations, 2016. **9**: p. 2059799116638003.
7. Luciano, A., G.R. Bond, and R.E. Drake, *Does employment alter the course and outcome of schizophrenia and other severe mental illnesses? A systematic review of longitudinal research.* Schizophrenia research, 2014. **159**(2-3): p. 312-321.
8. Gilbert, E. and S. Marwaha, *Predictors of employment in bipolar disorder: a systematic review.* Journal of Affective Disorders, 2013. **145**(2): p. 156-164.
9. Schofield, P. and J. Das-Munshi, *Big data: what it can and cannot achieve.* BJPsych Advances, 2018. **24**(4): p. 237-244.
10. Aldekhyyel, R., et al. *Content and quality of free-text occupation documentation in the electronic health record.* in *AMIA Annual Symposium Proceedings*. 2016. American Medical Informatics Association.
11. Lovis, C., R.H. Baud, and P. Planche, *Power of expression in the electronic patient record: structured data or narrative text?* International Journal of Medical Informatics, 2000. **58**: p. 101-110.
12. Wu, C.-Y., et al., *Evaluation of smoking status identification using electronic health records and open-text information in a large mental health case register.* PloS one, 2013. **8**(9): p. e74262.
13. Jackson, R.G., et al., *Natural language processing to extract symptoms of severe mental illness from clinical text: the Clinical Record Interactive Search Comprehensive Data Extraction (CRIS-CODE) project.* BMJ open, 2017. **7**(1): p. e012012.
14. Iqbal, E., et al., *Identification of adverse drug events from free text electronic patient records and information in a large mental health case register.* PloS one, 2015. **10**(8): p. e0134208.
15. Chandran, D., et al., *Use of Natural Language Processing to identify Obsessive Compulsive Symptoms in patients with schizophrenia, schizoaffective disorder or bipolar disorder.* Scientific reports, 2019. **9**(1): p. 1-7.
16. Fernandes, A.C., et al., *Identifying suicide ideation and suicidal attempts in a psychiatric clinical research database using natural language processing.* Scientific reports, 2018. **8**(1): p. 1-10.
17. Kreimeyer, K., Foster, M., Pandey, A., Arya, N., Halford, G., Jones, S. F., ... & Botsis, T. (2017). Natural language processing systems for capturing and standardizing unstructured clinical information: a systematic review. *Journal of biomedical informatics*, *73*, 14-29.
18. Koleck, T. A., Dreisbach, C., Bourne, P. E., & Bakken, S. (2019). Natural language processing of symptoms documented in free-text narratives of electronic health records: a systematic review. *Journal of the American Medical Informatics Association*, *26*(4), 364-379.
19. Peckham, T. K., Baker, M. G., Camp, J. E., Kaufman, J. D., & Seixas, N. S. (2017). Creating a future for occupational health. *Annals of Work Exposures and Health*, *61*(1), 3-15.
20. Djumalieva, J., Lima, A., & Sleeman, C. (2018). *Classifying occupations according to their skill requirements in job advertisements* (No. ESCoE DP-2018-04). Economic Statistics Centre of Excellence (ESCoE).

For peer review only - http://bmjopen.bmj.com/site/about/guidelines.xhtml

21. Tixier, A. J. P., Hallowell, M. R., Rajagopalan, B., & Bowman, D. (2016). Automated content analysis for construction safety: A natural language processing system to extract precursors and outcomes from unstructured injury reports. *Automation in Construction*, *62*, 45-56.

22. Perera, G., et al., *Cohort profile of the South London and Maudsley NHS Foundation Trust Biomedical Research Centre (SLaM BRC) case register: current status and recent enhancement of an electronic mental health record-derived data resource.* BMJ open, 2016. **6**(3): p. e008721.

23. Cunningham, H., et al., *Getting more out of biomedical documents with GATE's full lifecycle open source text analytics.* PLoS computational biology, 2013. **9**(2): p. e1002854.

24. Deleger, L., Li, Q., Lingren, T., Kaiser, M., & Molnar, K. (2012). Building gold standard corpora for medical natural language processing tasks. In *AMIA Annual Symposium Proceedings* (Vol. 2012, p. 144). American Medical Informatics Association.

25. Hollister, B.M., et al. *Development and performance of text-mining algorithms to extract socioeconomic status from de-identified electronic health records*. in *PACIFIC SYMPOSIUM ON BIOCOMPUTING 2017*. 2017. World Scientific.

26. Yang, H. and J.M. Garibaldi, *Automatic detection of protected health information from clinic narratives.* Journal of biomedical informatics, 2015. **58**: p. S30-S38.

27. Dehghan, A., et al. *Identification of occupation mentions in clinical narratives*. in *International Conference on Applications of Natural Language to Information Systems*. 2016. Springer.

28. Viani, N., et al. *Time Expressions in Mental Health Records for Symptom Onset Extraction*. in *Proceedings of the Ninth International Workshop on Health Text Mining and Information Analysis*. 2018.

29. Chilman, N.G.M., & Das-Munshi, J., *Sociodemographic predictors of unemployment in patients with severe mental illness: an electronic health record cohort study.* Retrieved from osf.io/rx7zs, 2020

For peer review only - http://bmjopen.bmj.com/site/about/guidelines.xhtml

Table 1 Sociodemographic and clinical features of the Clinical Record Interactive Search (CRIS) case register.*

	**No. patients, % (Total N=341,720)**
**AGE**	
16-29	84,181 (24.63%)
30-49	123,216 (36.06%)
50-69	79,880 (23.38%)
70-89	43,852 (12.83%)
90+	10,591 (3.10%)
**GENDER**	
Male	166,480 (48.72%)
Female	175,007 (51.21%)
Other/Not Known	233 (0.07%)
**ETHNICITY**	
White British	136,289 (39.88%)
Irish	5,182 (1.70%)
Black Caribbean	34,229 (10.02%)
Black African	15,654 (4.58%)
Indian	4,345 (1.27%)
Pakistani	1,852 (0.54%)
Bangladeshi	1,088 (0.32%)
Chinese	1,124 (0.33%)
Other Asian	5,500 (1.61%)
Other Ethnic Group	19,650 (5.75%)
Other White	22,076 (6.46%)
Mixed	1,879 (0.55%)
Not Known	92,222 (26.99%)
**MARITAL STATUS**	
Married/civil partnership/cohabiting	46,617 (13.64%)
Divorced/separated/civil partnership dissolved	17,309 (5.07%)
Widowed	15,758 (4.61%)
Single	141,111 (41.29%)
Not Known	120,925 (35.39%)
**LOCAL QUARTILES OF NEIGHBOURHOOD DEPRIVATION**	
Least deprived	79,537 (23.28%)
3rd Quartile	80,049 (23.43%)
2nd Quartile	79,767 (23.34%)
Most deprived	79,829 (23.36%)
Address Not Known	22,538 (6.60%)
**PRIMARY DIAGNOSIS**	
F30-F39: mood (affective) disorders	37,796 (11.06%)
F00-F09: organic, including symptomatic, mental disorders	29,801 (8.72%)
F10-F19: mental and behavioural disorders due to psychoactive substance misuse	27,870 (8.16%)

For peer review only - http://bmjopen.bmj.com/site/about/guidelines.xhtml

F20-F29: schizophrenia, schizotypal and delusional disorders	18,253 (5.34%)
F40-F49: neurotic, stress-related and somatoform disorders	31,962 (9.35%)
F50-F59: behavioural syndromes associated with physiological disturbances and physical factors	9,166 (2.68%)
F60-F69: disorders of adult personality and behaviour	6,605 (1.93%)
F70-F79: mental retardation	2,732 (0.80%)
F80-F89: disorders of psychological development	5,874 (1.72%)
F90-F98: behavioural and emotional disorders with onset usually occurring in childhood and adolescence	12,028 (3.52%)
Other diagnosis	83,847 (24.54%)
Not Known	75,786 (22.18%)
**QUARTILES OF 'EVENTS' ENTERED INTO THE HEALTH RECORD**	
No Events	50,673 (14.83%)
Least Events (1-3)	86,818 (25.41%)
2nd Quartile (4-10)	62,804 (18.38%)
3rd Quartile (11-40)	68,774 (20.13%)
Most Events (41+)	72.651 (21.26%)
**INPATIENT BED DAYS**	
No inpatient admissions	311,099 (91.04%)
Low (1-2 days)	1,937 (0.50%)
Moderate (3-31 days)	10,587 (3,10%)
High (32+ days)	18,337 (5.37%)
**At the time of the occupation application run (29.01.2020).*	

For peer review only

*Table 2 Results from crude and multivariable logistic regression analyses examining predictors of occupation recording from the Clinical Record Interactive Search (CRIS) case register. **

	N (%) with at least one occupation retrieved by structured field/text-mining extractions	OR (95% CI)	aOR[1] (95% CI)	aOR[2] (95% CI)
**AGE**				
16-29	41,653 (49.48)	Reference	Reference	Reference
30-49	68,422 (55.53%)	**1.27 (1.25-1.30)**	**1.56 (1.53-1.59)**	**1.72 (1.68-1.75)**
50-69	49,289 (61.70%)	**1.65 (1.61-1.68)**	**1.98 (1.93-2.02)**	**2.19 (2.14-2.25)**
70-89	27,175 (61.97%)	**1.66 (1.63-1.70)**	**1.71 (1.67-1.76)**	**1.60 (1.54-1.65)**
90+	7,077 (66.82%)	**2.06 (1.97-2.15)**	**2.14 (2.04-2.24)**	**2.00 (1.89-2.11)**
**GENDER**				
Male	96,141 (57.75%)	Reference	Reference	Reference
Female	97,443 (55.68%)	**0.92 (0.91-0.93)**	**0.88 (0.87-0.90)**	**0.87 (0.85-0.88)**
Other/Not Known	32 (13.73%)	**0.12 (0.08-0.17)**	**0.10 (0.07-0.15)**	**0.16 (0.10-0.24)**
**ETHNICITY**				
White British	91,575 (67.19%)	Reference	Reference	Reference
Irish	4,303 (74.04%)	**1.39 (1.31-1.48)**	**1.24 (1.17-1.33)**	**1.23 (1.15-1.31)**
Black Caribbean	24,753 (72.32%)	**1.28 (1.24-1.31)**	0.99 (0.96-1.02)	**1.06 (1.03-1.09)**
Black African	11,341 (72.45%)	**1.28 (1.24-1.33)**	**1.07 (1.03-1.11)**	**1.12 (1.07-1.17)**
Indian	2,876 (66.19%)	0.96 (0.90-1.02)	**0.91 (0.85-0.97)**	**0.91 (0.85-0.98)**
Pakistani	1,185 (63.98%)	**0.87 (0.79-0.95)**	**0.81 (0.73-0.90)**	**0.82 (0.74-0.91)**
Bangladeshi	719 (66.08%)	0.95 (0.84-1.08)	0.90 (0.78-1.03)	0.94 (0.82-1.08)
Chinese	690 (61.39%)	**0.78 (0.69-0.88)**	**0.73 (0.65-0.84)**	**0.81 (0.71-0.92)**
Other Asian	3,543 (64.42%)	**0.88 (0.84-0.94)**	**0.82 (0.78-0.87)**	**0.85 (0.80-0.91)**
Other ethnic Group	11,768 (59.89%)	**0.73 (0.71-0.75)**	**0.77 (0.75-0.80)**	**0.75 (0.72-0.77)**
Other White	14,610 (66.18%)	**0.96 (0.93-0.98)**	**0.94 (0.91-0.97)**	0.97 (0.94-1.00)
Mixed Race	1,197 (63.70%)	**0.86 (0.78-0.94)**	**0.68 (0.61-0.75)**	**0.78 (0.70-0.87)**
Not Known	25,056 (27.17%)	**0.18 (0.18-0.19)**	**0.31 (0.31-0.32)**	**0.50 (0.49-0.51)**

For peer review only - http://bmjopen.bmj.com/site/about/guidelines.xhtml

**MARITAL STATUS**				
Married/Civil Partnership/Cohabiting	31.037 (66.58%)	Reference	Reference	Reference
Divorced/Separated/Civil Partnership Dissolved	13,346 (77.10%)	**1.69 (1.62-1.76)**	**1.47 (1.40-1.53)**	**1.41 (1.35-1.47)**
Widowed	11,309 (71.77%)	**1.28 (1.23-1.33)**	1.05 (1.00-1.09)	**1.05 (1.01-1.10)**
Single	98,841 (70.04%)	**1.17 (1.15-1.20)**	1.02 (1.00-1.05)	**1.24 (1.21-1.27)**
Not Known	39,083 (32.32%)	**0.24 (0.23-0.25)**	**0.33 (0.32-0.33)**	**0.49 (0.47-0.50)**
**LOCAL QUARTILES OF NEIGHBOURHOOD DEPRIVATION**				
Least Deprived	48,155 (60.54%)		Reference	Reference
3rd Quartile	47,583 (59.44%)	**0.96 (0.94-0.97)**	**0.97 (0.95-0.99)**	**0.96 (0.94-0.99)**
2nd Quartile	45,842 (57.47%)	**0.88 (0.86-0.90)**	**0.94 (0.91-0.96)**	**0.93 (0.91-0.95)**
Most Deprived	41,800 (52.36%)	**0.72 (0.70-0.73)**	**0.89 (0.87-0.91)**	**0.88 (0.86-0.90)**
Address Not Known	10,236 (45.42%)	**0.54 (0.53-0.56)**	**0.70 (0.67-0.72)**	**0.77 (0.74-0.80)**
**DIAGNOSIS**				
F30-F39: mood (affective) disorders	27,057 (71.59%)	Reference	Reference	Reference
F00-F09: organic, including symptomatic, mental disorders	20,269 (68.01%)	**0.84 (0.82-0.87)**	**0.91 (0.88-0.94)**	**0.71 (0.68-0.74)**
F10-F19: mental and behavioural disorders due to psychoactive substance misuse	18,150 (65.12%)	**0.74 (0.72-0.77)**	**0.71 (0.68-0.73)**	**0.47 (0.45-0.49)**
F20-F29: schizophrenia, schizotypal and delusional disorders	14,645 (80.23%)	**1.61 (1.54-1.68)**	**0.87 (0.83-0.91)**	**0.78 (0.74-0.82)**
F40-F49: neurotic, stress-related and somatoform disorders	19,920 (62.32%)	**0.66 (0.64-0.68)**	**0.75 (0.72-0.77)**	**0.76 (0.73-0.79)**
F50-F59: behavioural syndromes associated with physiological disturbances and physical factors	5,287 (57.68%)	**0.54 (0.52-0.57)**	**0.65 (0.62-0.68)**	**0.68 (0.64-0.72)**

For peer review only - http://bmjopen.bmj.com/site/about/guidelines.xhtml

F60-F69: disorders of adult personality and behaviour	4,739 (71.75%)	1.01 (0.95-1.07)	**0.68 (0.64-0.73)**	**0.77 (0.72-0.82)**
F70-F79: mental retardation	2,277 (83.35%)	**1.99 (1.79-2.20)**	**1.81 (1.63-2.03)**	**1.69 (1.51-1.90)**
F80-F89: disorders of psychological development	4,377 (74.78%)	**1.16 (1.09-1.24)**	**1.22 (1.14-1.30)**	**1.78 (1.66-1.92)**
F90-F98: behavioural and emotional disorders with onset usually occurring in childhood and adolescence	8,754 (72.78%)	**1.06 (1.01-1.11)**	**1.25 (1.19-1.32)**	**1.84 (1.74-1.93)**
Other diagnosis	43,787 (52.22%)	**0.43 (0.42-0.45)**	**(0.68-0.72)**	**0.76 (0.73-0.78)**
Not Known	24,354 (32.14%)	**0.19 (0.18-0.19)**	**0.44 (0.43-0.45)**	**0.66 (0.64-0.68)**
**QUARTILES OF 'EVENTS' ENTERED INTO THE HEALTH RECORD**				
No Events	12,012 (23.70%)	Reference	Reference	Reference
Least Events	35,009 (40.32%)	**2.17 (2.12-2.23)**	**2.18 (2.13-2.23)**	**1.75 (1.70-1.79)**
2nd Quartile	34,368 (54.72%)	**3.89 (3.79-3.99)**	**3.89 (3.79-3.99)**	**2.79 (2.71-2.87)**
3rd Quartile	49,237 (71.59%)	**8.11 (7.90-8.33)**	**8.06 (7.85-8.28)**	**5.01 (4.86-5.16)**
Most Events	62,990 (86.70%)	**20.98 (20.37-21.60)**	**18.89 (18.29-19.50)**	**9.77 (9.43-10.1)**
**INPATIENT BED DAYS**				
No inpatient admissions	167,213 (53.75%)	Reference	Reference	Reference
Low (1-2 days)	1,408 (82.97%)	**4.19 (3.69-4.76)**	**1.87 (1.64-2.14)**	**1.68 (1.47-1.93)**
Moderate (3-31 days)	8,714 (82.31%)	**4 (3.81-4.21)**	1.06 (1.00-1.11)	1.01 (0.95-1.07)
High (32+ days)	16,281 (88.79%)	**6.81 (6.51-7.14)**	**1.57 (1.49-1.66)**	**1.32 (1.25-1.39)**

*All variables listed in this table had a strong association with the outcome variable (p<.0001), assessed by likelihood ratio tests.
[1]Adjusted for service contact variables (no. of events and inpatient bed days)
[2]Adjusted for all other variables in the table

For peer review only - http://bmjopen.bmj.com/site/about/guidelines.xhtml

FIGURE CAPTIONS

Figure 1: A step-by-step illustration of the methods used for the occupation application development and evaluation, with the number and types of documents used at each step.

Figure 2: The process undertaken by the occupation application when text-mining occupations from the clinical free-text text.

Figure 3: The study population selection and extraction results from text-mining occupations from the Clinical Record Interactive Search case register.

For peer review only

For peer review only - http://bmjopen.bmj.com/site/about/guidelines.xhtml

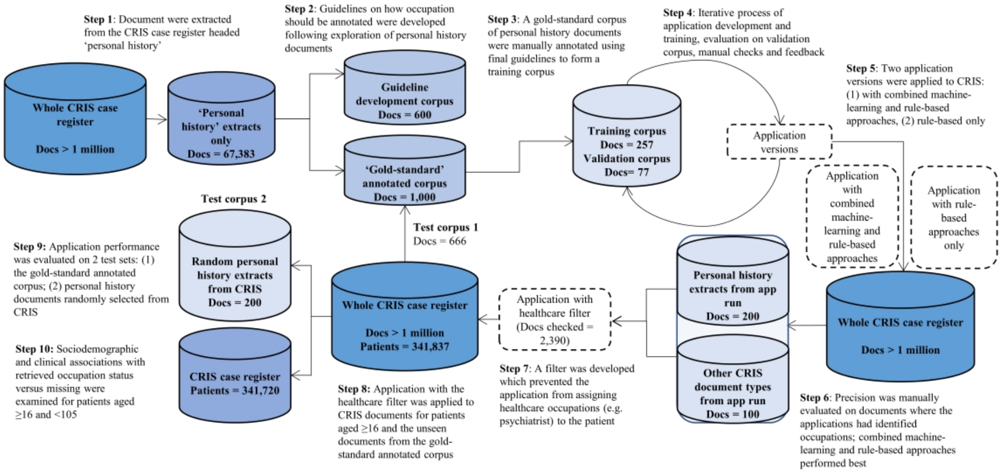

Figure 1: A step-by-step illustration of the methods used for the occupation application development and evaluation, with the number and types of documents used at each step.

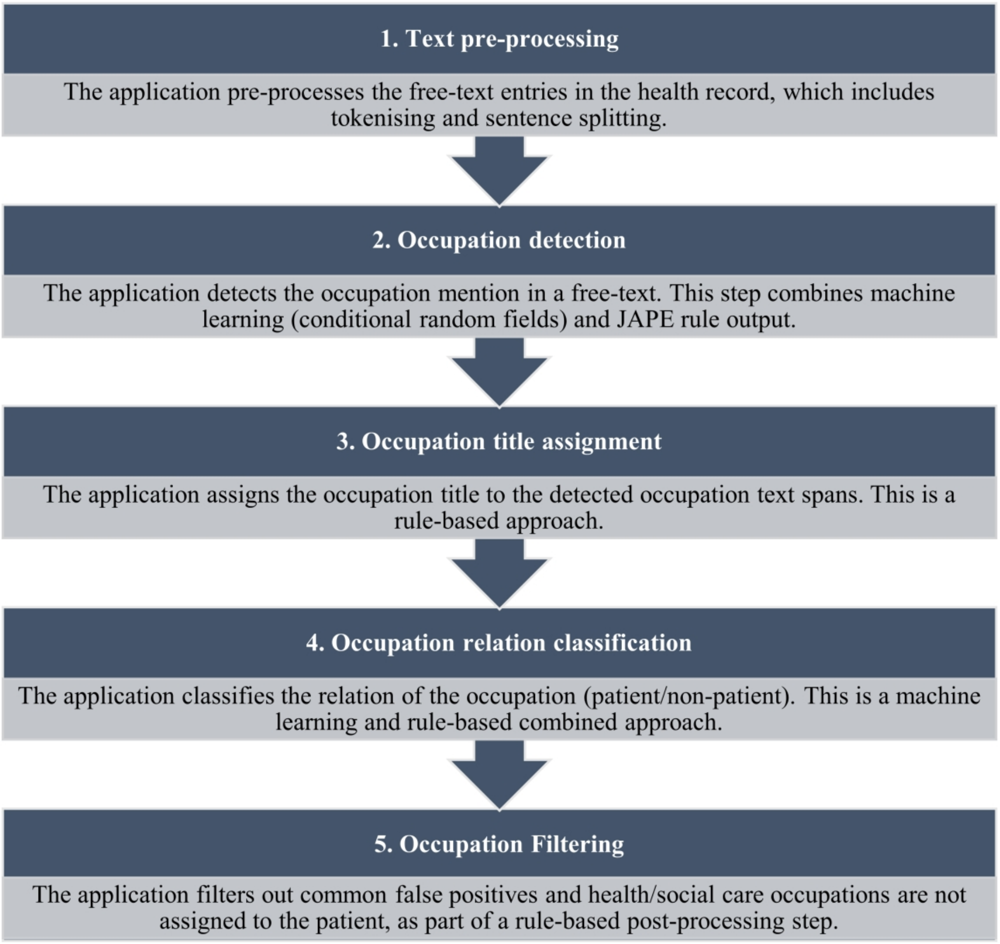

Figure 2: The process undertaken by the occupation application when text-mining occupations from the clinical free-text text.

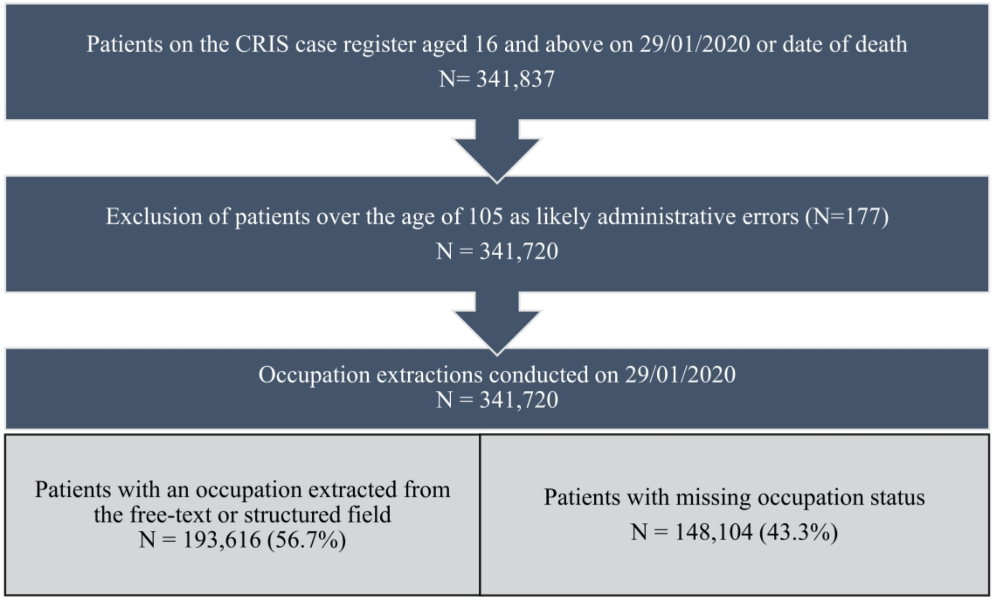

Figure 3: The study population selection and extraction results from text-mining occupations from the Clinical Record Interactive Search case register.

Supplementary File 1: Descriptions of the datasets used in the development, testing and implementation of the occupation application

Application Development and Testing Datasets			
	**Type of document**	**Document count**	**No. of Occupation Annotations (manual)**
Validation corpus	Personal history	77 (+256 documents used in training)	405
Testing corpus 1: with vs without machine-learning comparison	Personal history + other CRIS documents	200	521
Testing corpus 2: gold-standard annotated documents	Personal history	666	3,429
Testing corpus 4: Unannotated documents	Personal history	200	442
**Application Implementation Dataset**			
	Type of document	**Patient count**	**No. Of Occupation Extractions (application)**
CRIS case register of patient records aged >=16	Attachments	341,720	21,321,757 (all relations)
	Events		
	Correspondence		
	Discharge Notification Summaries		
	History		

For peer review only

	Mental State Formulations		
	Presenting Circumstances		
	Risk Events		
	Social Situation		
	Ward Progress Notes		

Table 1: Descriptions of the datasets used in the development, testing and implementation of the occupation application

For peer review only

OCCUPATION ANNOTATION GUIDELINES

Authors:

Background – Natasha Chilman & Esther Tolani

Annotation rules – Esther Tolani, Angus Roberts, Zoe Chui, Karen Birnie, Lisa Harber-Aschan, Billy Gazard, Anna Kolliakou & Jayati Das-Munshi

General Tips – Esther Tolani

Appendices – Natasha Chilman & Anna Kolliakou

With thanks to Shirlee MaCrimmon for annotation support.

Supplementary File 2 to accompany the BMJ Open paper, "Text-mining occupations from the mental health electronic health record: a natural language processing approach using the Clinical Record Interactive Search (CRIS) platform to identify patient occupations in south London, UK."

Background 2
Setting 2
Development 2
Annotation rules 5
Annotation rules 6
Occupation 8
Subject of Occupation 13
Exclusion Criteria 15
General Tips 16
References 17
Appendix 1 17
A timeline of actions leading to guideline and application development 17
Appendix 2 22
Annotation Guidelines Version 1 22

For peer review only

Background

The CRIS-occupation-application has been developed to enable researchers to extract occupations from the Clinical Record Interactive Search (CRIS) case register. When using the occupation application, it is important to consider how it has been trained and tested to allow for appropriate use of the application and accurate interpretation of results. These guidelines provide clear and transparent rules which specify how occupations should be annotated manually in free-text EHRs, which then informed the development of the occupation application, and a gold-standard against which the application was evaluated against.

Setting

These occupation annotation guidelines were developed over the years 2017-2020 for use on psychiatric clinical texted accessed through the Clinical Record Interactive Search (CRIS) application. CRIS is a large de-identified case register of electronic health records, comprising of the Electronic Patient Health Journal notes used in South London and Maudsley NHS Trust (SLaM). SLaM is the largest unit mental health provider of secondary services in Europe, serving 1.3 million people across the London boroughs of Lambeth, Southwark, Lewisham and Croydon. The SLaM CRIS case register stores over 350,000 patient records to date, and encompasses a range of secondary mental health services (including inpatient and community mental health services) [1]. Whilst this annotation guideline was written following the exploration of CRIS text extracts, we also recommend that the guidelines can be used as a starting point when extracting occupations from other CRIS systems and psychiatric Electronic Health Records (EHRs) in the UK.

It is important to remember that EHRs are a secondary routine data resource in research, they are used primarily for a practical purpose by clinicians to document patient-level information. This context should be kept in mind when considering the complexity of annotating occupations.

Development

Here we summarise the actions taken to develop the guidelines and describe how the guidelines have changed over time. This is also detailed further in the development timeline (Appendix 1).

These guidelines were based on the 'personal history' sections of the free-text entries. When clinicians use 'personal history' as a header in the free-text fields in CRIS, the text which follows typically includes information on the patient's upbringing and family life,

For peer review only - http://bmjopen.bmj.com/site/about/guidelines.xhtml

education and – most importantly for our interests – occupation. Personal history sections were chosen as the best place to start when examining how occupation is described in the free-text fields in CRIS. An application previously developed by Dr David Chandran in the Biomedical Research Centre was used to extract personal history documents from CRIS to develop and test the annotation guidelines. A 'document' is a single section of a free-text field in CRIS, for example a letter attachment or event progress note. One patient may have more than one personal history section in their record.

Initial guidelines were drawn from the exploration of 100 personal history documents and team discussions. From the first draft, the occupation annotation guidelines were developed based on the premise that when an occupation is annotated in the free-text, two components must be specified: the occupation (feature) and subject of the occupation (relation). Occupation is a complex concept and can be written as a job title (e.g. a waiter) or a description of a work activity (e.g. serving tables).

The guidelines were developed through an iterative process of document annotation, team discussions and rule development (Appendix 1). 600 personal history documents were annotated throughout this process which informed and tested the sufficiency of the guidelines to instruct occupation annotation. Out of these 600 documents, 250 personal history documents were double annotated. Inter-annotator agreements were calculated throughout the guideline development stages to assess whether the guidelines were sufficient for occupation to be annotated consistently (Appendix 1). By November 2017, 200 further personal history documents were double-annotated with good inter-rater reliability between two manual annotators, with a Cohen's Kappa statistic of 77% for occupation and 72% for relation. This is considered a good level of agreement. 800 documents were then annotated by a single annotator using the latest guideline and together these formed the 1000 document gold-standard annotated document corpus. This corpus was later used for application development (forming the training corpus).

To demonstrate how the guidelines have changed over time, please see Appendix 2 which shows the Guidelines Version 1 (GV1). When compared to the current guidelines, a significant level of detail has been added since the initial draft. For example, there is now a section the beginning of the guidelines stating which parts of a sentence describing occupation should be annotated. Whilst re-drafting the guidelines, an 'additional information' column was added to give further detail on how the annotation rules work, which researchers found helpful when completing annotations. The later drafts of the guidelines also add a 'blank' annotation rule: if the occupation title can be inferred by the text itself then the occupation feature should be left empty, and the relation was determined by the sentence structure. This was important when later evaluating the application, as a bespoke GATE evaluation package was used to take this rule into account. All changes that were made to the annotation guideline throughout the development process were agreed within the research team.

For peer review only - http://bmjopen.bmj.com/site/about/guidelines.xhtml

The following guideline is the final annotation guideline document. Whilst there were some small formatting changes made during application development (Appendix 1), the rules in this guideline were used when annotating the 1000 gold-standard training and testing corpus for the application.

For peer review only

For peer review only - http://bmjopen.bmj.com/site/about/guidelines.xhtml

Annotation rules

Esther Tolani, Zoe Chui, Karen Birnie, Angus Roberts, Anna Kolliakou, Jayati Das-Munshi, Robert Stewart

These guidelines outline the process for annotating occupation status in GATE. The term(s) highlighted should be the word(s) in the free text which indicate(s) the occupation of an individual. After reading the free text, annotations should be made on the word(s) which is (are) related to an employment status or an occupation: job or profession. For all cases, each annotation will have the following features: **occupation and subject of occupation (relation).**

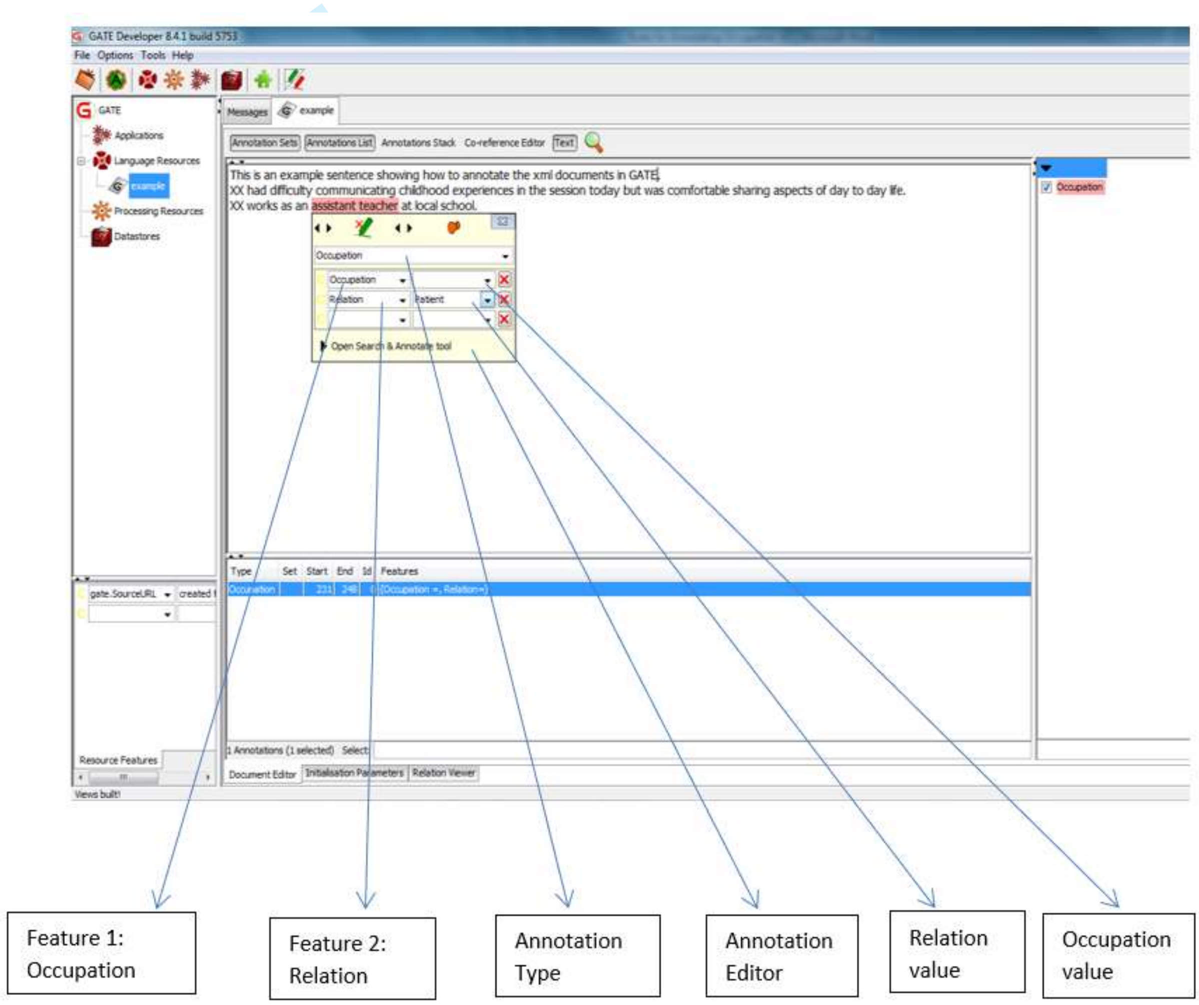

Figure 1: A labelled example illustrating how occupation is annotated in GATE software

Sentence Structure of Annotation

The annotation should be made on adjectives, nouns and verbs in the sentence.

- Title of Occupation

Titles of occupations are always nouns.

Adjectives should only be annotated when they are part of the occupation type or necessary for describing the occupation e.g. assistant manager, senior consultant. The annotation value is left empty when occupation can be inferred from the exact annotated text.

Example:

XX worked as an assistant teacher – occupation value: empty.

She is a mental health nurse – occupation value: empty

Annotate the adjective and noun.

- **Description of Occupation**

  A) Description of occupation consists of verbs referring to work activities.

Annotate text following:

1) Works for/in/as/at…

Works for real estate - occupation value: estate agent

Works for British Gas - occupation value: British Gas worker

Works for investment bank – occupation value: investment bank worker

2) Job/Role involves, has to do with, includes…

Job involves cleaning houses – occupation value: house cleaner

Role involves writing, teaching – occupation value: writer, teacher

3) Verbs indicating membership

Joined the navy – occupation value: navy officer

Example:

XX worked joined the army after moved to the UK – occupation value: army officer.

Annotate the verb and noun because the noun or verb alone does not describe the occupation sufficiently.

Annotation rules

An occupation or description of work should be annotated regardless of whether it is current or past. However, text indicating whether occupation or description of work is current or past is not required for the annotation unless it offers information on the stability/transience of the occupation.

Examples:

XX is not working at the moment – occupation value: unemployed

XXX has been working as a chef for 3 years- occupation value: chef

For peer review only - http://bmjopen.bmj.com/site/about/guidelines.xhtml

XXX worked briefly or worked for a few months or worked every summer – occupation value: other

Do not annotate:

- Punctuation
  - e.g. full stops, semi-colons…
- Adverbs
  - e.g. happily, works hard…
- Articles in front of occupation
  - e.g. the, as, an, a…
- Conjunctions
  - e.g. and, but, if…

*[**UNLESS** these are articles and conjunctions in a double annotation as further below]*

- Adjectives when describing a quality assigned to a job
  - e.g. experienced teacher, qualified electrician
- Verbs that precede title of occupation
  - e.g. became, moved to, promoted to, went to, decided to, etc.
- Text around title of occupation describing place of work **unless** text around title of occupation refers to a field or sector
  - e.g. assistant manager for a phone company – value empty
  - e.g. assistant manager in sales – value fill Sales Assistant Manager
- Time frames or duration of work
  - e.g. worked for 5 years, was a chef in 1995, has worked, is not working

*[**UNLESS** it offers information on the stability/transience of the occupation ie worked briefly or worked for a few months or worked every summer]*

Double annotation:

In the case of two joint occupation descriptions, annotate the same text twice and give a different value each time.

Examples:
Annotate once: he worked in a clothes shop and a kitchen – occupation value: retail worker
Annotate twice: he worked in a clothes shop and a kitchen – occupation value: other - kitchen

Please use this double annotation as sparingly as possible and not when clearly stated occupations or different occupations/work descriptions are joined as below.

Examples:
He worked as a chef and cleaner – two annotations with blank values

For peer review only - http://bmjopen.bmj.com/site/about/guidelines.xhtml

He worked on building sites and roofing - two annotations with first value 'labourer' and second 'labourer' or 'other'.

Long job descriptions:
Sometimes clinical record notes are written in a rich and speech-like manner. In cases like this, it is best to annotate a longer piece of text then risk leaving out valuable information.

Examples:
She has worked for only 1 and half year in her life in a wine bar 28yrs ago – occupation value: other- bar

He used to work every summer with his brother at a car wash – occupation value: other-car wash

Occupation

For the occupation value, a title for the work described should be entered. If no title can be created from a work description, 'other' should be entered in the occupation value. In addition, if the title is identical to the work described (job can be inferred from the annotated text), the occupation should be left empty.

Rules for annotating Employment Status				
Rule	Rule Description	Example	Occupation Value	Additional Information
Description of job is given, without job title	Annotate with closely related description	Daily role involves operating the machines	Machine operator	
Multiple occupations	All occupations mentioned in the free text (personal history) should be annotated even if they are or appear to be repetitions of an occupation already mentioned within the same history	Chef, 7.5-tonne truck driver<br><br>Worked as kitchen assistant…he helped in a kitchen for 6 months<br><br>She was a teacher…enjoyed her work as a teacher	[blank]<br>[blank]<br><br>Kitchen assistant<br>Other-kitchen<br><br>[blank]<br>[blank]	For chef, truck driver, kitchen assistant and teacher the occupation value should be left empty because the work descriptions are identical to the title that should be given. For 'helped in a kitchen' the occupation value should be 'other'
Related occupations	Annotate all occupations which are mentioned which are	Worked as a social worker and later became a manager	[blank]<br>[blank]	The occupation value should be left empty because the work

For peer review only - http://bmjopen.bmj.com/site/about/guidelines.xhtml

	associated with the progress of the same job			descriptions social worker and manager identical to the titles that should be given
Place, sector or employer is mentioned without occupation	Annotate the company or sector	XX works for the council<br>He has been with his present boss for a while	Council worker<br><br>Other	Annotate the company, sector or employer
Loose description of job role which cannot be titled	Annotate the reference to odd jobs which have relevance	XX does various jobs which include, tiling, plumbing…	Tiler, plumber	Annotate the word referring to the odd job

The section below outlines how to annotate the alternative employment statuses: student, retired, self-employed, unemployed, carer, homemaker and other.

**Rules for annotating Student Status**				
Rule	Rule Description	Example	Occupation Value	Additional Information
Student (full time/part time)	Annotate term student or a description of full time/ part time study. Include training/vocational courses.	XX is currently studying XX at university<br><br>He trained as a bricklayer<br><br>He trained in art<br><br>She attended university<br><br>Has a degree in Physics<br><br>He did a Masters in Psychology<br><br>Left University in 1995	Student<br><br>Student<br>[blank]<br><br>Student<br><br>Student<br><br>Student<br><br>Student<br><br>Student<br><br>Student	Two annotations are made to capture student status and occupation value of empty for bricklayer<br><br>'Trained' is annotated by itself whereas 'attended', 'did' 'degree' or 'undertook' need extra information annotated because out of context they wouldn't be sufficient by themselves

For peer review only

		Graduated with a degree in maths<br><br>He undertook the early career researcher training scheme	Student	
**Rules for annotating Retired Status**				
Retirement	Annotate the term retired or description of retirement	Worked until retirement	Retired	
**Rules for annotating Self-Employed Status**				
Self-employed without job description	Annotate the term or description of self-employed	Patient is self-employed	[blank]	The occupation value should be left empty because the job title self-employed is stated
		He owns a number of properties and shops	Self-employed	The occupation value should be self-employed. One annotation.
Self-employed with job description or business/property owner	Annotate the term or description of self-employed and job description	Patient is a self-employed builder	[blank]<br>[blank]	These should be two separate annotations (self-employed and builder). Occupation values should be left empty because the job titles self-employed and builder are stated
		He owns a number of properties and shops	Self-employed	One annotation

**Rules for annotating Other Employment Status**	

For peer review only - http://bmjopen.bmj.com/site/about/guidelines.xhtml

Difficult to define or job/ job role not stated<br>Simple reference to work	Annotate the verb 'work' or the noun 'job'	Works occasionally on weekends<br>Has had a few other jobs<br>He worked there for 4 years and then left<br>He worked in 1995<br>He worked hard all his life<br>He worked briefly when younger<br>He had a satisfactory job<br>She had numerous jobs<br>He has a creative job<br>She has had three other jobs<br>He did about 8 jobs	Other	Annotate the verb work by itself unless followed by an adverb providing more information about the work itself ie occasionally, the number of jobs ie numerous or the quality ie hard, creative
Sector is not mentioned	Statements not referring to a specific sector or industry should not be annotated	XX moved to the private sector	Other-private	
Army/Navy occupations	Annotate relevant word/ phrase	XX joined the army	Army officer	Always annotate as army or navy officer
Job or occupation relating to shops	Annotate relevant word/ phrase	XX worked part-time in WHSmith	Retail worker	Always annotate as retail worker
Sector or place of work is mentioned but unclear what job the subject undertook	Annotate relevant word/phrase	XX joined his brother in construction<br><br>XX worked in a kitchen	Other-construction	It is not clear what job in construction the patient did so occupation value is given 'other'
**Rules for annotating Unemployed Status**				
Unemployment	Annotate the term unemployed or the description of unemployment	XX has not worked for several years<br>XXX does not work anymore	Unemployed	Unemployment is usually stated in various ways. If the word unemployed is

For peer review only - http://bmjopen.bmj.com/site/about/guidelines.xhtml

For peer review only

		XXX lost his job<br>XXX ran out of work a year ago<br>XXX is currently not working<br>XXX got sacked<br>XXX was made redundant<br>XXX stopped working<br>XXX cannot remember the last job she had<br>Last job was about 5 years ago<br><br>XXX is unemployed	[blank]	annotated, the annotation value should be blank
**Rules for annotating Homemaker Status**				
Housewife househusband	Annotate the term that states that an individual is a homemaker	Mother was a housewife…	[blank]	The occupation value should be left empty because housewife status is stated
**Rules for annotating Carer Status**				
Carer	Annotate the term carer or the description of care role	XX is a carer for elderly mother<br><br>XXX was a carer	Carer<br><br>[blank]	Annotation value of carer should be entered if text annotated includes who the person cared for is. In the second case, where this is not stated, the occupation value is left empty because carer status is stated
**Rules for annotating Volunteer**				
Volunteer	Annotate the noun volunteer or the verb volunteering	XX volunteered with the	Volunteer	

For peer review only - http://bmjopen.bmj.com/site/about/guidelines.xhtml

		council once a week		
**Rules for annotating National Service**				
National Service	Annotate the noun national service and the verb preceding	He joined national service<br><br>He did his national service<br><br>He finished national service	Other – national service	
**Rules for annotating illegal activities**				
Prostitution	Annotate relevant word/phrase	XX was working as a prostitute	Sex-worker	Always annotate as sex-worker
Jobs of questionable status/legality	Text referring to income generating jobs that might not be legal	He was a brothel owner<br><br>She made money from dealing drugs	Other- brothel<br><br>Other – drug dealing	Other plus an indication of place or type of work

Subject of Occupation

The relation value should state who the occupation refers to/who carries out the job described. In most cases, the occupation belongs to the patient. The occupation can also belong to the parent/carer of the patient, spouse, relative or other.

**Rules for annotating Subject of Occupation**				
Rule	Rule Description	Example	Relation Value	Additional Information
Patient	The occupation annotated should belong to the patient	Patient was a butcher for XX years	Patient	
Parent/ Carer / Guardian	The occupation annotated should belong to the father or mother of the patient.	Father works as a mechanic	Father	
Spouse	The occupation annotated should belong to the spouse	Husband works for the government doing research	Spouse	The occupation of the spouse should still be recorded even if the text suggests they are no longer together
Relative	The occupation annotated belongs to a	XX's brother discussed the issues	Brother	Relations include: sibling,

For peer review only - http://bmjopen.bmj.com/site/about/guidelines.xhtml

	family member of the patient who is not the parent/carer or spouse	faced being XX's carer and working as a shop assistant…		cousin, aunt, uncle, niece, child, nephew, and grandchild
Girlfriend/Boyfriend/ Partner	The occupation annotated should belong to the patient's girlfriend/boyfriend	XX's girlfriend was a carer for the elderly	Girlfriend	
Other	The occupation annotated does not belong to the patient or patient's relative	The nurse came round to see XX	Other	

For peer review only

Exclusion Criteria

Rule	Rule Description	Example	Value	Additional information
Future Plans	Future plans to work should not be annotated	XX plans to start role	No annotation	
Hypothetical statements	Text referring to hypothetical scenarios or worries about losing job	XX said he would have left his job if he thought he couldn't cope<br><br>XX was worried he was going to get sacked<br><br>She would have quit if they hadn't given her a raise	No annotation<br><br>No annotation<br><br>No annotation	
When 'work' is used as an adjective<br><br>Describing quality of work without explicitly stating having one		She had great work ethics<br><br>Her work performance deteriorated<br><br>She didn't like her work colleague<br><br>Her job was really good<br><br>He didn't enjoy working there	No annotation	

For peer review only

For peer review only - http://bmjopen.bmj.com/site/about/guidelines.xhtml

General Tips: THINK LIKE AN OCCUPATION MACHINE!

1) The machine doesn't have any context

We annotate personal history segments which, if rich, give us a good idea of an individual's story. The machine does not have that reference and if, for example, we annotate 'stopped' in "he worked for 5 years and then stopped" as 'unemployed' we are essentially teaching it to recognise the word 'stopped' as referring to unemployment. Imagine what will happen when we run this application all over CRIS! Ask, if unsure - does the machine understand the annotation I have assigned regardless of context? What will happen if it learns to recognise it as such in another context?

2) The machine loves more of the same

You come across a personal history segment that has 'worked' 3 times, 'labourer' 2 times, 'jobs' 4 times and 2 'sacked'. The machine doesn't know that these have been repeated as it has no context. Also, the more 'labourers' it gets fed, the more it will learn to unequivocally recognise them automatically in any context. Annotate them all!

3) The machine is as smart as you

If you feel you are spending too long making annotation decisions or find a rule that is making your annotations inconsistent, the machine will think the same. Ask questions no matter how silly they seem!

For peer review only

References

1. Perera, G., et al., *Cohort profile of the South London and Maudsley NHS Foundation Trust Biomedical Research Centre (SLaM BRC) case register: current status and recent enhancement of an electronic mental health record-derived data resource.* BMJ open, 2016. **6**(3): p. e008721.

Appendix 1

A timeline of actions leading to guideline and application development

Natasha Chilman, Anna Kolliakou

Date	Action	Outcome
July 2017	Preliminary meeting with research team<br><br>Esther annotated 100 personal history extracts with no guidelines<br><br>Feedback on annotations and guideline ideas discussed by research team	Development of GV1 (Guidelines Version 1)
August 2017	Anna M annotated 50 of the above 100 extracts using GV1, recommended changes	Development of GV2
August 2017	Comments given by research team on GV2	Development of…<br>GV2.1<br>GV2.2<br>GV2.3<br>GV2.4
September 2017	1,262 personal history documents extracted:<br>- Esther annotated 500 using GV2.4<br>- Shirlee double-annotated 200 of these using GV2.	Inter-annotator agreement for 200 double-annotated documents: Cohen's Kappa calculated by GATE = 72% for occupation, 87% for relation<br><br>Development of GV2.5
October-November 2017	40 case examples were written by Anna K and annotated by Anna K, Lisa, Billy, Shirlee and Angus.	Collective agreements made on rules<br><br>Started development of GV2.6
November 2017	Above case examples were given to Karen and Zoe who annotated according to GV2.5	Collective agreements made on rules

For peer review only - http://bmjopen.bmj.com/site/about/guidelines.xhtml

		Finished development of GV2.6
November-December 2017	1000 new personal history documents extracted:<br>- Karen annotated all 1000 using GV2.6<br>- Zoe double-annotated 200 of these using GV2.6	Inter-annotator agreement for 200 double-annotated documents:<br>Cohen's Kappa =<br>77% for occupation<br>72% for relation<br><br>In total, 1000 documents = 'gold-standard' annotated corpus
March 2018	GV2.6 was finalised and so was re-named GV3. The 1000 annotated documents were stratified by gender, length of extract, and occupation feature type (labelled as 'other' vs 'non-other' – see guideline for more detail).	334/1000 stratified annotated extracts were sent to Xingyi (University of Sheffield) to develop the application: 257 were used as a training set, 77 were used as a validation set.
April 2018	Application version 1 (AV1) created by Xingyi, sent to Anna K who manually checked application output on the 77 test corpus and precision, recall and F-measures were calculated by GATE evaluation package, feedback provided to Xingyi on application areas for improvement.	Development of AV2
April 2018	As above: AV2 ran on 77 documents, manually checked and measures calculated by GATE evaluation package, feedback provided.	Development of AV3
June 2018	As above: AV3 ran on 77 documents, manually checked and measures calculated by GATE evaluation package, feedback provided.	Development of AV3.1
August 2018	AV3.1 included three different application versions, all ran on 77 documents, manually checked and measures calculated by GATE evaluation package, feedback provided.	Development of AV3.2
November 2018	AV3.2 (one version) was run on 77 documents. The GATE evaluation package was under-estimating the performance of the application, as it classified that if an occupation feature was 'blank' then it was not labelled correctly. Please see guideline for instructions on use of 'blank' feature annotations. These type of annotations came up often in the text.	Development of AV3.3

For peer review only - http://bmjopen.bmj.com/site/about/guidelines.xhtml

	A new evaluation package ('revised' GATE evaluation) was created which correctly identified 'blank' annotations as a hit. This increased the F-measure and was felt to more accurately reflect the application performance when checking the output manually.<br><br>A small formatting change was made to the guideline, creating GV4, but there was no change in rule content.<br><br>Further feedback sent to Xingyi.	
November-December 2018	AV3.3 was run on the 77 documents, manually checked and F-measures calculated by revised GATE evaluation package, feedback sent to Xingyi.<br><br>A decision was made that the 77 documents needed to be re-annotated which was completed by Anna K in December 2018.	AV3.3 was updated
January-February 2019	Updated AV3.3 was run on both newly annotated 77 documents and previously annotated 77 documents. Barely any difference found in impact on F measure (a very small increase: old annotations F=0.890, new annotations F=0.896).<br><br>Updated AV3.3 run on newly annotated 77 documents, manually checked and F measures calculated by revised GATE evaluation package, feedback sent to Xingyi.	Development of AV3.4
April 2019	As the application was performing reasonably well on the 77 personal history documents, AV3.4 was run on the whole of CRIS. Anna K eyeballed the output and sent feedback to Xingyi for areas for improvement.	AV3.4 was updated to two versions: AV3.4(with machine learning) and AV3.4Revised (without machine learning)
June-July 2019	Both AV3.4 and AV3.4Revised were run on whole CRIS. Anna and Natasha manually checked 200 random personal-history-only documents, and 100 random CRIS documents. Areas for application improvement were sent to Xingyi.	Development of AV4
August 2019	AV4(ML) and AV4(Revised) were run on the whole CRIS. Training corpus of 77 documents was used to evaluate application on GATE. Anna and Natasha manually checked 200 random personal history-only documents, and 100 random CRIS documents (test corpus).	Results from performance of both applications on training corpus and test corpus is available in Supplementary File 3. Application reached good levels of performance

For peer review only - http://bmjopen.bmj.com/site/about/guidelines.xhtml

		(precision and recall all >0.79 on a test corpus). The machine learning application performed slightly better so this was chosen over the rule-based approach. However occupation ownership remained an issue, where many of the occupations retrieved belonged to people other than the patient e.g. clinicians. The application did not consistently annotate the relation of the occupation correctly, for example often 'psychiatrist' was annotated as belonging to the patient.
September-November 2019	Following occupation ownership issues identified in the manual evaluation, team meetings were held and it was decided to add an occupation 'filter' to the application. This is a list of occupations which have the most common incorrect relations (e.g. psychiatrist, social worker) – where the application incorrectly annotates the occupation as belonging to the patient. The occupations included in the filter will be assigned a 'other' relation, rather than 'patient' relation. This will mean that we can be more confident that the occupation extracted belongs to the patient. The team reflected that we may miss a small number of true positives this way (e.g. psychiatrists who are patients), but the risk of retrieving incorrect patient occupations is greater, plus healthcare professionals often go to different occupational services for mental health support so are less likely to be included in this sample of electronic health records.<br><br>Method:<br>- Natasha extracted occupations with ≥100 annotations across CRIS. She then sorted these occupations into 3 categories: those which should definitely be added to the filter (e.g. psychiatrist), those which she was not sure about (e.g. interpreter) and those not to add to the filter (e.g. construction).<br>- Out of those which she was not sure about, Natasha checked between 10-40 documents for the number of true positives retrieved by the application	AV4 with machine learning was updated by Xingyi to include the occupation filter, where the occupations on the filter list were assigned the relation 'other' rather than patient.

For peer review only

	(where the occupation was annotated correctly as belonging to the patient). During this process Natasha checked a total of 2,390 documents.<br>- Jay and Anna then went through this list to make collective decisions with Natasha on the unsure occupations. The filter list of occupations was then sent to research team for approval, then sent to Xingyi to add to the app.	
January-February 2020	The application was run over the whole of CRIS with the health/social care occupation filter applied.	Natasha firstly checked accuracy of 400 annotations made by the application: 200 from personal history documents only (precision all annotations = 96.00%, precision patient annotations only = 97%), and 200 annotations over other CRIS document types (precision all annotations = 93.00%; precision patient annotations only = 66%). Of the last estimate, many false positives were for occupation annotations for 'other'.
February 2020	Natasha checked 200 'other' occupation annotations to test the accuracy of this annotation and whether it should be excluded.	Precision for 'other' annotations only reached 23.5%. The false positives for this annotation seemed to fit 3 categories: text about job-seeking (e.g. looking for work), text about working on health/personal goals (e.g. working on his anxiety) or other incorrect annotations (e.g. blood work).
March 2020	Natasha looked at recall and precision more closely. Jyoti ran the application over the personal history table in gate (with extracts accessed via Dave Chandran's personal history app). Natasha selected 200 random documents from this personal history table, annotated them according to this occupation annotation guideline (excluding 'other' annotations), and then checking to see whether the app had identified these occupations (recall) or had identified any false positives (precision). As patient occupations are only mentioned rarely in the clinical record, it was not feasible to do a recall/precision check on all other types CRIS documents, therefore personal history	When looking at all occupation relation annotations, the app had a precision level of 90.04 and recall level of 85.77. When looking at patient relation only annotations, the application reached precision of 77.33 and recall of 79.37.

For peer review only - http://bmjopen.bmj.com/site/about/guidelines.xhtml

	documents are chosen as a targeted and feasible document to check.	

Appendix 2

Annotation Guidelines Version 1

Date: 04/08/2017

This guideline outlines the process for annotating occupation status in GATE. The term highlighted should be the word(s) in the free text which indicates the occupation of an individual, as described in the personal history of the patient. After reading the free text, annotations should be made on the word(s) which are related to an employment status or an occupation: job or profession. For all cases, each annotation will have the following features: **occupation and subject of occupation.** The exclusion criteria outline when no annotations should be made.

**Rules for annotating Occupation Status**			
Rule	Rule Description	Example	Actual Annotation
Multiple occupations	All occupations mentioned in the free text (personal history) should be annotated	Chef, 7.5-tonne truck driver	Occupation: chef, truck driver
Working role is given, without occupation mentioned	Annotate with closely related description	Daily role involves operating the machines	Occupation: production worker/machine operator
Related occupations	Annotate all occupations which are mentioned which are associated with the progress of the same job	Worked as a social worker and later became a manager	Occupation: social worker, social work manager
Place or sector is mentioned without occupation	Annotate the company or sector	XX works for the council	Occupation: council worker
Loose description of job role which cannot be titled	Annotate the reference to odd jobs which have relevance	XX does various jobs which include, tiling, plumbing…	Occupation: Tiler and Plumber

For peer review only - http://bmjopen.bmj.com/site/about/guidelines.xhtml

Rules for annotating Student Status			
Student (full time/part time)	Annotate term student or a description of full time/ part time study	XX is currently studying XX at university	Occupation: student
**Rules for annotating Retired Status**			
Retirement	Annotate the term retired or description of retirement	Worked until retirement	Occupation: retired
**Rules for annotating Self-Employed Status**			
Self-employed without job description	Annotate the term or description of self-employed	Patient is self-employed	Occupation: self-employed
Self-employed with job description	Annotate the term or description of self-employed and job description	Patient is a self-employed builder	Occupation: self-employed, builder
**Rules for annotating Other Occupation Status**			
Difficult to define or job/role not stated	Annotate relevant phrase	Works occasionally on weekends	Occupation: other
**Rules for annotating Unemployed Status**			
Unemployment	Annotate the term unemployed or the description of unemployment	XX has not worked for several years	Occupation: unemployed

For peer review only - http://bmjopen.bmj.com/site/about/guidelines.xhtml

Supplementary File 3: Machine learning and rule-based approaches to text-mine occupations from the electronic health record

The Occupation Application Pipeline

The occupation extraction application works by implementing 5 steps: 1) Text pre-processing, 2) Occupation mention detection, 3) Occupation title assignment, 4) Occupation relation extraction and 5) Occupation filtering. The pipeline of the application is demonstrated in Figure 1.

For a free-text input, we pre-process the input document through: (1) an English Tokeniser, (2) GATE's Morphological Analyser (lemmatise and tokens), (3) a sentence splitter (as the occupation extraction is conducted at sentence level), (4) a POS tagger (where we obtain part-of-speech for each token, and the part-of-speeches are used as features in later rule and machine-learning modules), and (5) ANNIE Name Entity Transducer (the default Name Entity Transducer embedded in the GATE system; these entities are used as features in later rule and machine learning modules).

After text pre-processing, we detect occupation mentions in the free-text by using: (1) a Conditional Random Field algorithm-based machine learning approach, and (2) a JAPE rule based approach. We combine the results from both approaches to increase the recall level. A rule-based title assignment module is applied to assign the occupation titles (e.g. builder, doctor, etc) for extracted occupation mentions.

When identifying who the occupation belongs to ('relation' extraction), we first extract the relation phrases (e.g. patient, mother, etc) from the surrounding context of the occupation mention. We then use a rule-based and machine-learning (support vector machine)-based classifier to classify the occupation relation. In this application we prefer rule-based relation classifier output to the machine-learning output when available – the machine-learning relationship is only used when there is no output from the rules.

The final step of the pipeline is occupation filtering, which is a rule-based approach to filter out common false positives and health/social care occupations.

For peer review only

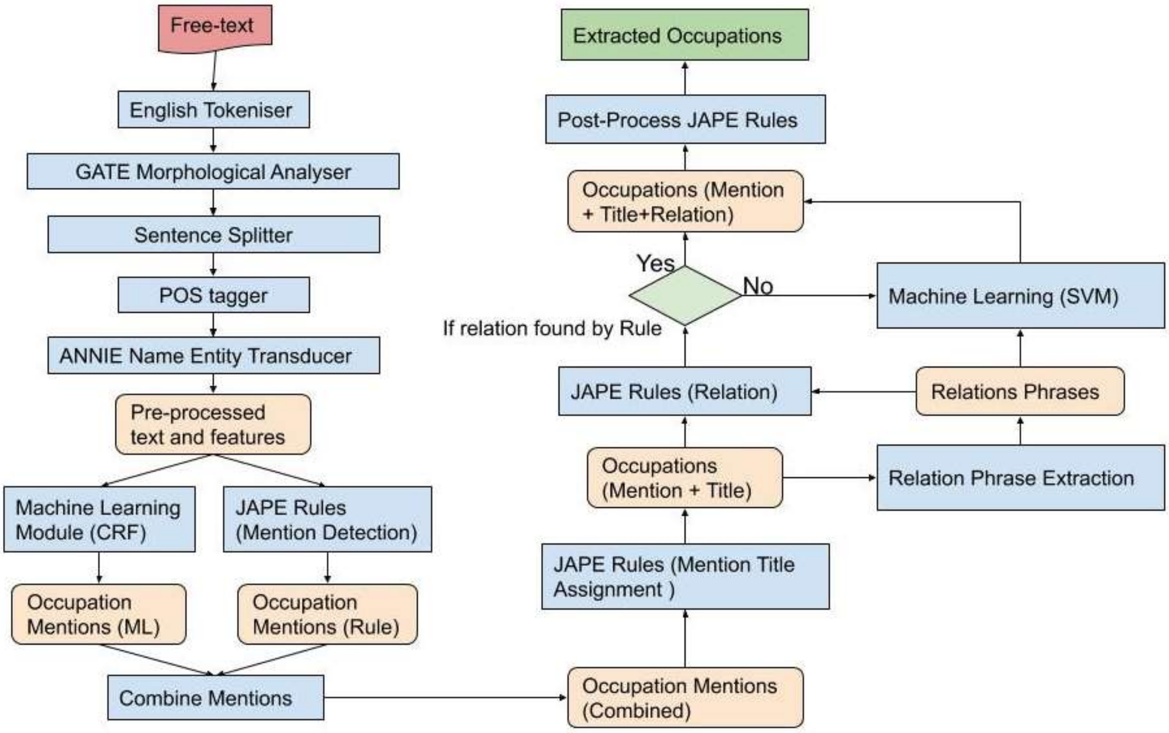

Figure 1: The pipeline of the occupation application. [1]

[1] The red box represents the input text, blue boxes represent NLP modules, light orange boxes represent the intermediate output from the NLP modules and the green box represents the extracted occupation.

Comparing combined machine-learning and rule-based approaches with rule-based only approaches

During testing we evaluated two versions of the application: one with machine-learning and rule-based combined approaches, and one with rule-based approaches only (without machine-learning). In the application version with rule-based approaches only, all machine-learning components in the occupation application pipeline (Figure 1) were removed.

The two versions of the applications were run over free-text documents in the case register of electronic health records. Where an occupation was identified by at least one of the application versions, we extracted 100 documents which included sections of text entitled 'personal history' and 100 documents which did not include a 'personal history' section (e.g. other 'Events' or 'Attachments'). One document may have multiple occupation annotations – all of which were evaluated. Where an occupation was annotated correctly this was counted as a true positive for occupation precision; where who the occupation belonged to was annotated correctly this was counted as a true positive for occupation relation; and where both were correct this was counted as an overall true positive for precision (table 1).

Both applications performed similarly, however the application with machine learning performed best on both personal history and other document types when assigning the occupation 'relation' (relation precision=0.91 on personal history documents). As the authors wanted to maximise precision regarding who the occupation belonged to (particularly for the patient), this application version was chosen for further developments.

**Documents**	**Application version**	**Precision**	**Occupation precision**	**Relation precision**
100 personal history	With Machine-Learning	0.92	0.96	0.91
	Without Machine-Learning	0.95	0.96	0.85
100 other CRIS document types	With Machine-Learning	0.79	1	0.68
	Without Machine-Learning	0.94	1	0.58

Table 1: Evaluation of occupation applications on the test corpus of documents where the applications had identified an occupation, calculated manually.
**Precision = true positive annotations/all annotations*
*** Occupation precision = true positive occupation titles/all occupation titles*
****Relation precision = true positive relation assignments/all relation assignments*

The RECORD statement – checklist of items, extended from the STROBE statement, that should be reported in observational studies using routinely collected health data.

	Item No.	STROBE items	Location in manuscript where items are reported	RECORD items	Location in manuscript where items are reported
**Title and abstract**					
	1	(a) Indicate the study's design with a commonly used term in the title or the abstract (b) Provide in the abstract an informative and balanced summary of what was done and what was found	Page 1, title<br><br>Page 3, abstract	RECORD 1.1: The type of data used should be specified in the title or abstract. When possible, the name of the databases used should be included.<br><br>RECORD 1.2: If applicable, the geographic region and timeframe within which the study took place should be reported in the title or abstract.<br><br>RECORD 1.3: If linkage between databases was conducted for the study, this should be clearly stated in the title or abstract.	Page 1, title<br><br>Page 1, title<br>Page 3, abstract<br><br>N/A
**Introduction**					
Background rationale	2	Explain the scientific background and rationale for the investigation being reported	Page 5, introduction		
Objectives	3	State specific objectives, including any prespecified hypotheses	Page 5, introduction, paragraph 3		
**Methods**					
Study Design	4	Present key elements of study design early in the paper	Page 5, introduction, paragraph 3<br><br>Figure 1		
Setting	5	Describe the setting, locations, and relevant dates, including	Page 6, materials & methods, paragraph 1		

For peer review only

		periods of recruitment, exposure, follow-up, and data collection			
Participants	6	*(a) Cohort study* - Give the eligibility criteria, and the sources and methods of selection of participants. Describe methods of follow-up<br>*Case-control study* - Give the eligibility criteria, and the sources and methods of case ascertainment and control selection. Give the rationale for the choice of cases and controls<br>*Cross-sectional study* - Give the eligibility criteria, and the sources and methods of selection of participants<br><br>*(b) Cohort study* - For matched studies, give matching criteria and number of exposed and unexposed<br>*Case-control study* - For matched studies, give matching criteria and the number of controls per case	N/A	RECORD 6.1: The methods of study population selection (such as codes or algorithms used to identify subjects) should be listed in detail. If this is not possible, an explanation should be provided.<br><br>RECORD 6.2: Any validation studies of the codes or algorithms used to select the population should be referenced. If validation was conducted for this study and not published elsewhere, detailed methods and results should be provided.<br><br>RECORD 6.3: If the study involved linkage of databases, consider use of a flow diagram or other graphical display to demonstrate the data linkage process, including the number of individuals with linked data at each stage.	Page 6, materials & methods, paragraph 2<br><br>Figure 1<br><br>Page 6, materials & methods, paragraph 3<br><br>N/A
Variables	7	Clearly define all outcomes, exposures, predictors, potential confounders, and effect modifiers. Give diagnostic criteria, if applicable.	Page 7, materials & methods, paragraph 1<br><br>Page 9, materials & methods, paragraphs 2 and 3	RECORD 7.1: A complete list of codes and algorithms used to classify exposures, outcomes, confounders, and effect modifiers should be provided. If these cannot be reported, an explanation should be provided.	Page 17, data sharing
Data sources/ measurement	8	For each variable of interest, give sources of data and details of methods of assessment (measurement).	Page 6, materials & methods, paragraph 2		

For peer review only

		Describe comparability of assessment methods if there is more than one group	Page 8, materials & methods, paragraph 4<br><br>Page 9, materials & methods, paragraphs 2 and 3		
Bias	9	Describe any efforts to address potential sources of bias	Page 9, materials & methods, paragraph 3		
Study size	10	Explain how the study size was arrived at	Page 9, materials & methods, paragraph 2		
Quantitative variables	11	Explain how quantitative variables were handled in the analyses. If applicable, describe which groupings were chosen, and why	Page 9, materials & methods, paragraphs 2 and 3		
Statistical methods	12	(a) Describe all statistical methods, including those used to control for confounding<br>(b) Describe any methods used to examine subgroups and interactions<br>(c) Explain how missing data were addressed<br>(d) *Cohort study* - If applicable, explain how loss to follow-up was addressed<br>*Case-control study* - If applicable, explain how matching of cases and controls was addressed<br>*Cross-sectional study* - If applicable, describe analytical methods taking account of sampling strategy	(a) Page 7, materials & methods, paragraph 2<br><br>Page 9, materials & methods, paragraphs 2 and 3<br><br>(b) N/A<br><br>(c) Page 9, materials & methods, paragraph 3<br><br>(d) N/A<br><br>(e) N/A		

For peer review only

		(e) Describe any sensitivity analyses			
Data access and cleaning methods		..		RECORD 12.1: Authors should describe the extent to which the investigators had access to the database population used to create the study population.<br><br>RECORD 12.2: Authors should provide information on the data cleaning methods used in the study.	Page 6, materials & methods, paragraphs 1 and 2<br><br>Figure 2 (text pre-processing)<br><br>Page 9, materials & methods, paragraph 2
Linkage		..		RECORD 12.3: State whether the study included person-level, institutional-level, or other data linkage across two or more databases. The methods of linkage and methods of linkage quality evaluation should be provided.	N/A
**Results**					
Participants	13	(a) Report the numbers of individuals at each stage of the study (*e.g.*, numbers potentially eligible, examined for eligibility, confirmed eligible, included in the study, completing follow-up, and analysed)<br>(b) Give reasons for non-participation at each stage.<br>(c) Consider use of a flow diagram	(a) Figure 1<br><br>Page 10, results, paragraph 4<br><br>Table 1<br><br>(b) Figure 1<br><br>(c) Figure 3 (flow diagram)	RECORD 13.1: Describe in detail the selection of the persons included in the study (*i.e.*, study population selection) including filtering based on data quality, data availability and linkage. The selection of included persons can be described in the text and/or by means of the study flow diagram.	Figure 1<br><br>Figure 3
Descriptive data	14	(a) Give characteristics of study participants (*e.g.*, demographic, clinical, social) and information on exposures and potential confounders	(a) Page 10, results, paragraph 5<br><br>Table 1		

For peer review only

		(b) Indicate the number of participants with missing data for each variable of interest<br>(c) *Cohort study* - summarise follow-up time (*e.g.*, average and total amount)	(b) Figure 3<br><br>Page 11, results, paragraph 1<br><br>Table 2		
Outcome data	15	*Cohort study* - Report numbers of outcome events or summary measures over time<br>*Case-control study* - Report numbers in each exposure category, or summary measures of exposure<br>*Cross-sectional study* - Report numbers of outcome events or summary measures	Page 11, results, paragraphs 1, 2 and 3		
Main results	16	(a) Give unadjusted estimates and, if applicable, confounder-adjusted estimates and their precision (e.g., 95% confidence interval). Make clear which confounders were adjusted for and why they were included<br>(b) Report category boundaries when continuous variables were categorized<br>(c) If relevant, consider translating estimates of relative risk into absolute risk for a meaningful time period	(a) Page 10, results, paragraph 3<br><br>Page 11, results, paragraph 5<br><br>Table 2<br><br>(b) Table 2<br><br>(c) N/A		
Other analyses	17	Report other analyses done—e.g., analyses of subgroups and interactions, and sensitivity analyses	N/A		
**Discussion**					
Key results	18	Summarise key results with reference to study objectives	Page 13, discussion, paragraph 1		

For peer review only

Limitations	19	Discuss limitations of the study, taking into account sources of potential bias or imprecision. Discuss both direction and magnitude of any potential bias	Page 13, discussion, paragraph 1<br><br>Page 14, discussion, paragraph 2	RECORD 19.1: Discuss the implications of using data that were not created or collected to answer the specific research question(s). Include discussion of misclassification bias, unmeasured confounding, missing data, and changing eligibility over time, as they pertain to the study being reported.	Page 14, discussion, paragraph 2
Interpretation	20	Give a cautious overall interpretation of results considering objectives, limitations, multiplicity of analyses, results from similar studies, and other relevant evidence	Page 13, discussion, paragraphs 1 and 2		
Generalisability	21	Discuss the generalisability (external validity) of the study results	Page 14, discussion, paragraph 2		
**Other Information**					
Funding	22	Give the source of funding and the role of the funders for the present study and, if applicable, for the original study on which the present article is based	Page 15, funding		
Accessibility of protocol, raw data, and programming code		..		RECORD 22.1: Authors should provide information on how to access any supplemental information such as the study protocol, raw data, or programming code.	Supplementary materials 1, 2 and 3<br><br>Page 17, data sharing

*Reference: Benchimol EI, Smeeth L, Guttmann A, Harron K, Moher D, Petersen I, Sørensen HT, von Elm E, Langan SM, the RECORD Working Committee. The REporting of studies Conducted using Observational Routinely-collected health Data (RECORD) Statement. *PLoS Medicine* 2015; in press.

*Checklist is protected under Creative Commons Attribution (CC BY) license.
